# Impaired AMPK control of alveolar epithelial cell metabolism promotes pulmonary fibrosis

**DOI:** 10.1172/jci.insight.182578

**Published:** 2025-07-01

**Authors:** Luis R. Rodríguez, Konstantinos-Dionysios Alysandratos, Jeremy Katzen, Aditi Murthy, Willy Roque Barboza, Yaniv Tomer, Sarah Bui, Rebeca Acín-Pérez, Anton Petcherski, Kasey Minakin, Paige Carson, Swati Iyer, Katrina Chavez, Charlotte H. Cooper, Apoorva Babu, Aaron I. Weiner, Andrew E. Vaughan, Zoltan Arany, Orian S. Shirihai, Darrell N. Kotton, Michael F. Beers

**Affiliations:** 1 Pulmonary, Allergy, and Critical Care Division, Department of Medicine, and; 2 PENN-CHOP Lung Biology Institute, Perelman School of Medicine at the University of Pennsylvania, Philadelphia, Pennsylvania, USA.; 3 Center for Regenerative Medicine, Boston University and Boston Medical Center, Boston, Massachusetts, USA.; 4 The Pulmonary Center and Department of Medicine, Boston University Chobanian & Avedisian School of Medicine, Boston, Massachusetts, USA.; 5 Departments of Medicine, Endocrinology, and Molecular and Medical Pharmacology, David Geffen School of Medicine at UCLA, Los Angeles, California, USA.; 6 Department of Biomedical Sciences, School of Veterinary Medicine, and; 7 Institute for Regenerative Medicine, University of Pennsylvania, Philadelphia, Pennsylvania, USA.; 8 Cardiovascular Institute, Perelman School of Medicine at University of Pennsylvania, Philadelphia, Pennsylvania, USA.

**Keywords:** Metabolism, Pulmonology, Adult stem cells, Fibrosis, Mitochondria

## Abstract

Alveolar epithelial type II (AT2) cell dysfunction is implicated in the pathogenesis of familial and sporadic idiopathic pulmonary fibrosis (IPF). We previously demonstrated that expression of an AT2 cell–exclusive disease-associated protein isoform (SP-C^I73T^) in murine and patient-specific induced pluripotent stem cell–derived (iPSC-derived) AT2 cells leads to a block in late macroautophagy and promotes time-dependent mitochondrial impairments; however, how a metabolically dysfunctional AT2 cell results in fibrosis remains elusive. Here, using murine and human iPSC-derived AT2 cell models expressing SP-C^I73T^, we characterize the molecular mechanisms governing alterations in AT2 cell metabolism that lead to increased glycolysis, decreased mitochondrial biogenesis, disrupted fatty acid oxidation, accumulation of impaired mitochondria, and diminished AT2 cell progenitor capacity manifesting as reduced AT2 cell self-renewal and accumulation of transitional epithelial cells. We identify deficient AMPK signaling as a critical component of AT2 cell dysfunction and demonstrate that targeting this druggable signaling hub can rescue the aberrant AT2 cell metabolic phenotype and mitigate lung fibrosis in vivo.

## Abstract 2

The study explores the role of AMP-kinase in the metabolic dysfunction of alveolar epithelial type II cells using murine and iPSC models of lung fibrosis.

## Introduction

Idiopathic pulmonary fibrosis (IPF), an enigmatic chronic interstitial lung disease, claims the lives of more than 40,000 Americans every year (1). In the early 2000s, the understanding of IPF shifted from an inflammation-driven paradigm to one of aberrant epithelial wound healing resulting in progressive lung fibrosis (2–5). Among the various types of lung epithelia, alveolar epithelial type II (AT2) cells were implicated in IPF due to histopathological findings of AT2 cell hyperplasia (6) and apoptosis (7). Within the alveolar niche, AT2 cells play a critical role in alveolar repair, acting as progenitors that self-renew and differentiate into AT1 cells to restore alveolar epithelial integrity after injury (8–13). This understanding, coupled with genetic studies demonstrating heritable interstitial lung disease–related variants in AT2 cell–specific surfactant protein genes (14), led to the concept of repetitive microinjuries in the alveolar space causing AT2 cell dysfunction and initiating the disease process. Recent innovative preclinical models have further demonstrated that pulmonary fibrosis stems from dysfunctional AT2 cell endophenotypes characterized by senescence (15), ER stress (16, 17), disrupted macroautophagy (18–20), telomere dysfunction (21, 22), and pro-inflammatory/pro-fibrotic signaling (18, 23). Consequently, AT2 cell dysfunction has emerged as a driver of sustained fibrosis, rather than AT2 cell loss (11), which results in a resolving fibrotic response (24, 25).

Understanding the pathobiology underlying IPF initiation and progression has been facilitated by the characterization of high effect size variants in the surfactant protein C (*SFTPC*) gene found in patients with familial pulmonary fibrosis, sporadic IPF, and childhood interstitial lung disease. Of particular relevance is the missense variant in the linker domain of the surfactant protein C (SP-C) pro-protein (*Sftpc*^I73T^) resulting in a mutant protein with altered intracellular trafficking patterns, including accumulation of misprocessed isoforms at the plasma membrane and within endosomal compartments (18, 26). In vitro studies using cell lines expressing SP-C^I73T^ revealed a dysfunctional epithelial phenotype marked by a late block in macroautophagy, impaired mitophagy, and defective cellular proteostasis (26). In addition, AT2 cells from *Sftpc*^I73T^ mice demonstrate a similar phenotype accompanied by an early multiphasic injury/alveolitis (7–14 days) followed by a transition to fibrosis (14–42 days) (18). In keeping with these observations, patient-specific *SFTPC*^I73T^ mutant iPSC-derived AT2 cells (iAT2s) display diminished AT2 cell progenitor capacity, proteostasis defects, mitochondrial impairments, and inflammatory activation (27). Notably, these alterations in cellular metabolism and mitochondrial homeostasis are consistent across species and various in vitro and in vivo platforms. Thus, we hypothesized that a conserved epithelial metabolic phenotype underlies the pathobiology of fibrotic lung disease.

Disruption to homeostatic metabolic pathways, including oxidative phosphorylation and glycolysis, has been recognized as a component of the fibrotic milieu for more than a decade (28–31). Early studies focused on metabolic changes in mesenchymal lineages (29, 30, 32–34), where increased glycolysis and lactate production were observed in activated fibroblasts and targeting glycolytic enzymes inhibited fibrogenesis in vitro and in mice in vivo (33, 35, 36). Alveolar epithelial mitochondrial impairments in IPF were first reported with findings of enlarged, dysmorphic mitochondria with impaired electron transport chain complex I and IV activity in AT2 cells from patients with IPF, a phenotype that was associated with decreased expression of the mitophagy regulator PTEN-induced kinase 1 (PINK1) (37). Concurrent defects in autophagy were evident, with increased levels of autophagy markers LC3B and p62 in hyperplastic AT2 cells within fibrotic regions of IPF lungs (37). Subsequently, alterations in mitochondrial homeostasis or activity have each been shown to affect AT2 cell function and lung fibrogenesis. For example, genetic disruption of mitochondrial networks in AT2 cells altered phospholipid and cholesterol metabolism (38), resulting in spontaneous lung fibrosis (38), while genetic manipulation of mitochondrial complex I activity altered AT2 cell progenitor function in vivo (39). Notably, defects in mitochondrial homeostasis of AT2 cells from IPF lungs and bleomycin-injured mouse lungs responded to thyroid hormone mimetics (40).

Despite these advances, questions remain about how AT2 cell metabolic dysfunction is mediated, how it leads to fibrosis, and whether restoring AT2 cell function can ameliorate established disease. Using murine and iPSC-derived AT2 cell models, we herein delineate the altered mitochondrial dynamics, respiration, and proglycolytic state associated with SP-C^I73T^ expression and identify AMPK as a druggable signaling hub able to rescue the aberrant AT2 cell metabolic phenotype and mitigate lung fibrosis.

## Results

### Murine AT2 cells expressing Sftpc^I73T^ exhibit a metabolic shift toward glycolysis.

**Figure 1:**
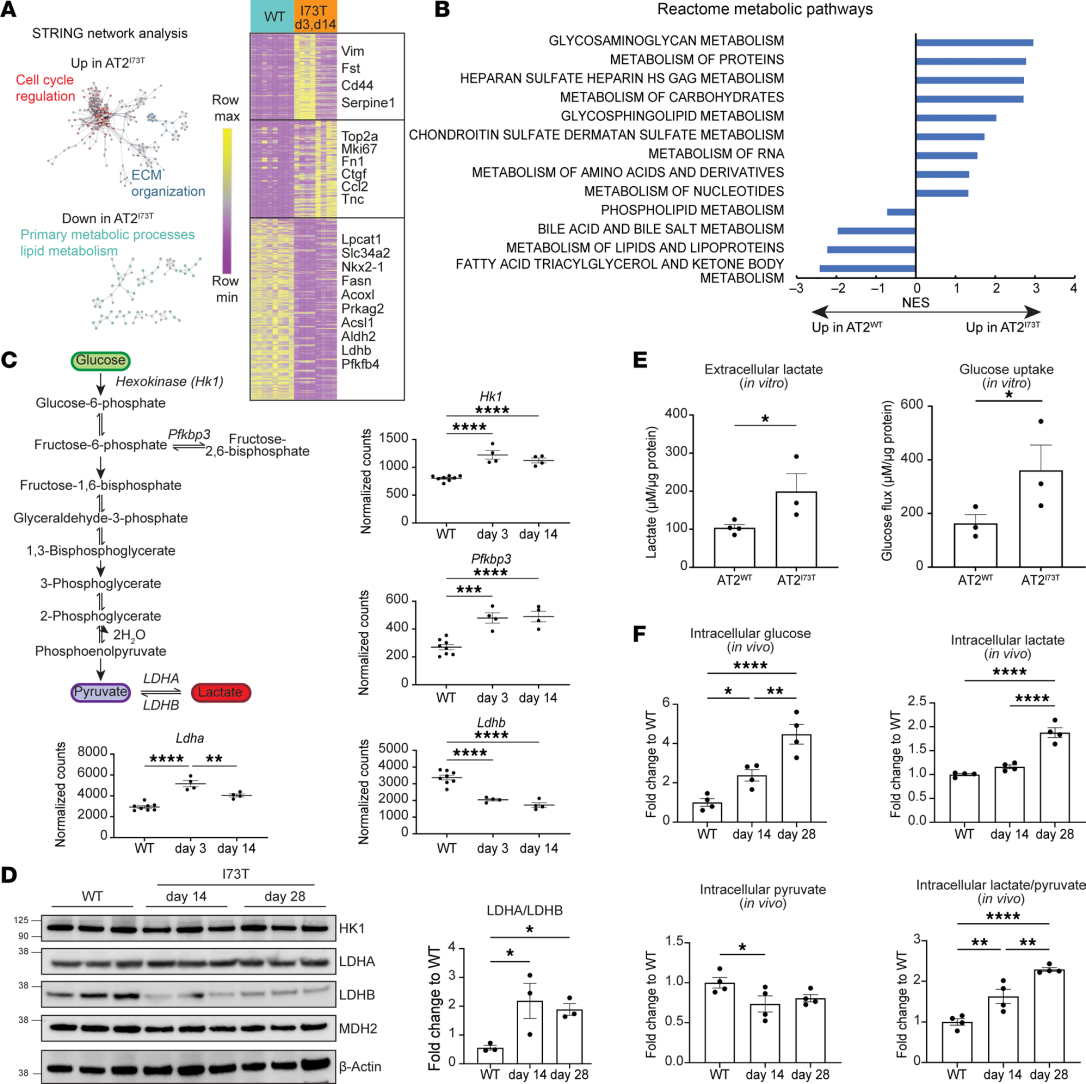
Increased glycolysis in murine AT2 cells expressing *Sftpc^I73T^*. (**A**) Unsupervised hierarchical clustering (Euclidean) heatmap of differentially expressed genes (DEGs; fold-change [FC] > 1.5; false discovery rate [FDR] < 0.05) in AT2^I73T^ cells 3 days and 14 days after in vivo tamoxifen induction (*n* = 4 per group) versus AT2^WT^ cells (age-matched C57B6/J mice, *n* = 8 by popRNA-Seq). A subset of DEGs is highlighted. STRING network analysis shows downregulation of genes associated with primary metabolic processes and lipid metabolism and upregulation of genes associated with proliferation and ECM organization in AT2^I73T^ cells. (**B**) Reactome pathway analysis of DEGs in AT2^I73T^ cells at 3 days and 14 days after tamoxifen induction demonstrates differential regulation of multiple metabolic pathways. (**C**) Schematic of rate-limiting enzymes in glycolysis pathway and individual graphs of normalized popRNA-Seq counts for highlighted genes in 3-day and 14-day AT2^I73T^ and AT2^WT^ cells. (**D**) Western blot of AT2^WT^ and AT2^I73T^ cells at peak of inflammation (14 days) and fibrosis (28 days) with densitometric quantification (mean±SEM; *n* = 3 biological replicates) showing differential LDHA and LDHB protein abundance. (**E**) Extracellular lactate and glucose concentrations (μM) in 48-hour ex vivo cultures of AT2^I73T^ cells (28 days after in vivo tamoxifen) and AT2^WT^ cells, measured by YSI biochemistry analyzer and normalized to total protein content (mean±SEM; *n* = 3 or 4 biological replicates). (**F**) Intracellular glucose, lactate, and pyruvate concentrations from 40,000 flow-sorted AT2 cells, reported as ratios to WT mean concentration (mean±SEM; *n* = 4 biological replicates). *P < 0.05, **P < 0.005, ***P < 0.0005, ****P < 0.00005 by ordinary 1-way ANOVA.

The ontogeny of lung fibrosis in *Sftpc*^I73T^ mice following tamoxifen induction of mutant protein expression is marked by 3 distinct phases: initiation (days 0–3), inflammation (days 3–14), and fibrogenesis (days 14–28) (18, 23). To delineate the sequence of AT2 cell changes during these epochs, we performed population RNA sequencing (popRNA-Seq) of AT2^I73T^ cells isolated at 2 key time points following in vivo tamoxifen administration, day 3 (“early” inflammation) and day 14 (transition from inflammation to fibrogenesis), and AT2^WT^ cells. We identified 1,921 and 2,627 differentially expressed genes (DEGs) in day 3 and day 14 AT2^I73T^ cells, respectively, compared with AT2^WT^ cells (for a total of 3,595 combined DEGs; Supplemental Figure 1; supplemental material available online with this article; https://doi.org/10.1172/jci.insight.182578DS1). Based on STRINGDB (41) and Enrichr (42), genes associated with cell cycle regulation and extracellular matrix organization were differentially upregulated in AT2^I73T^ cells, whereas genes associated with multiple metabolic processes were differentially downregulated (Figure 1A and Supplemental Figure 1). Reactome pathway analysis identified differential regulation of multiple metabolic pathways, including upregulation of carbohydrate and protein metabolism and downregulation of phospholipid and fatty acid metabolism pathways in AT2^I73T^ cells (Figure 1B).

In addition, transcripts regulating commitment steps in the glycolytic pathway, *Hk1* and *Pfkbp3*, were upregulated in AT2^I73T^ cells, and shifts in the lactate dehydrogenase A/lactate dehydrogenase B (*Ldha/Ldhb*) ratio (at both the transcript and protein levels; Figure 1, C and D, respectively), further indicated enhanced conversion of pyruvate to lactate. Functionally, AT2^I73T^ cells exhibited increased glucose uptake and lactate production in short-term in vitro cultures (Figure 1E), and intracellular measurements of isolated AT2 cells further demonstrated elevated glucose levels and higher lactate-to-pyruvate ratios over time (Figure 1F). Collectively, these findings suggest that murine AT2^I73T^ cells exhibit time-dependent metabolic alterations marked by an increased glycolytic state.

### Disrupted mitochondrial biogenesis and altered mitochondrial functions in murine AT2^I73T^ cells.

**Figure 2:**
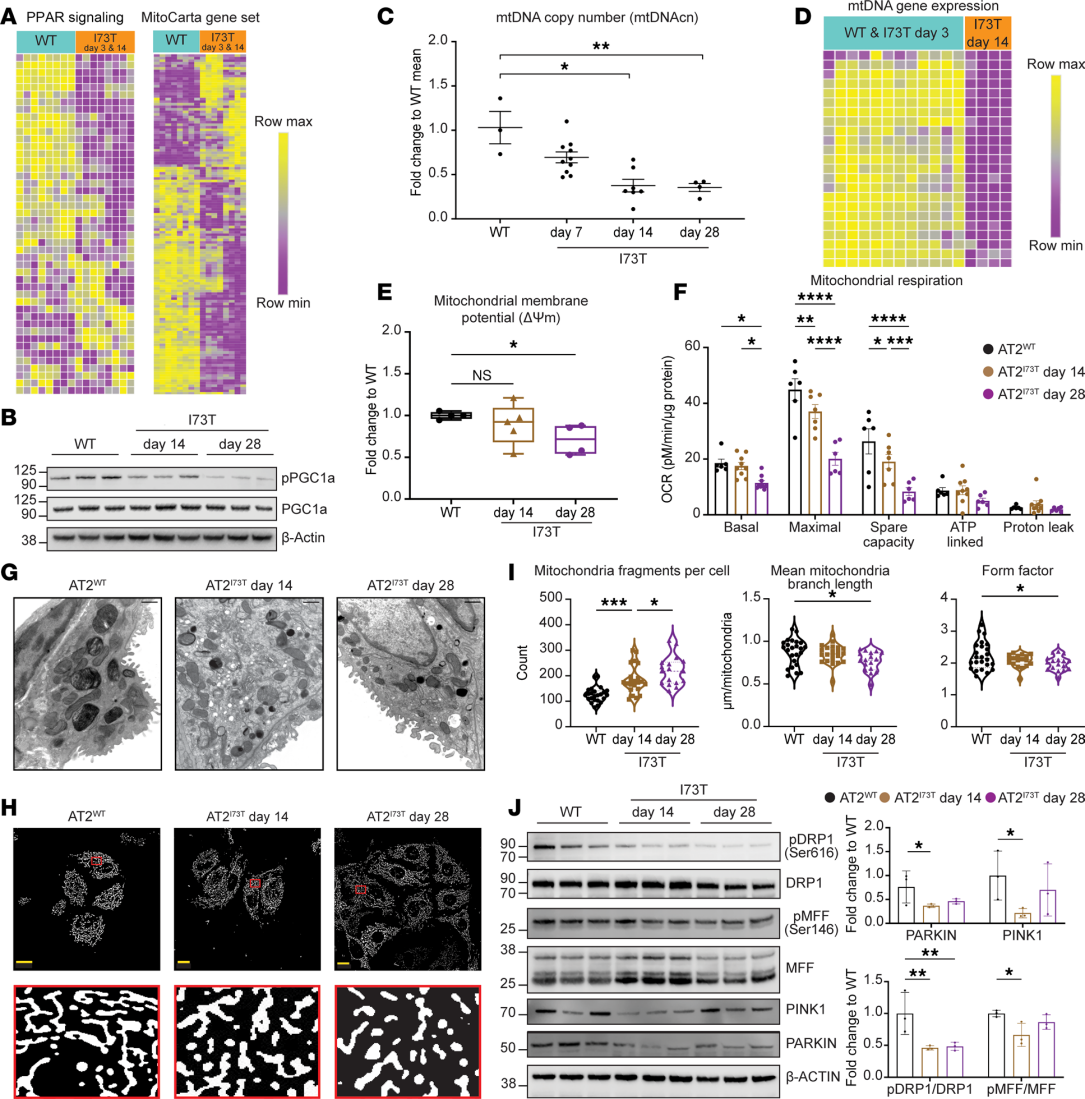
Defects in mitochondrial biogenesis and mitochondrial dynamics. (**A**) Unsupervised hierarchical clustering (Euclidean) heatmap of DEGs (FDR < 0.05) in the Hallmark PPAR signaling pathway and MitoCarta gene sets (row-normalized *z*-score) by popRNA-Seq demonstrates downregulation in AT2^I73T^ cells at days 3 & 14 after tamoxifen induction (*n* = 4 mice per I73T time point, *n* = 8 WT mice). (**B**) Western blot of phosphorylated and total PGC1α (*n* = 3 biological replicates). (**C**) Time-dependent reduction in mtDNA copy number in AT2^I73T^ (*n* = 3 mice for WT, *n* = 10 mice for day 7, *n* = 7 mice for day 14, *n* = 4 mice for day 28). (**D**) Unsupervised hierarchical clustering (Euclidean) heatmap of differentially expressed mtDNA genes by popRNA-Seq shows decreased expression in AT2^I73T^ cells at day 14 (*n* = 4 mice per I73T time point and *n* = 8 WT mice). (**E**) Flow cytometry analysis of mitochondrial membrane potential (ΔΨm) demonstrates a time-dependent reduction in tetramethylrhodamine, methylester, fluorescence in AT2^I73T^ cells (*n* = 4–5 mice per condition). Box plots show the interquartile range, median (line), and minimum and maximum (whiskers). (**F**) Measurement of oxygen consumption rate (OCR) shows a time-dependent reduction in basal and maximal uncoupled mitochondrial respiration and spare respiratory capacity in AT2^I73T^ cells (*n* = 4–9 mice per condition). (**G**) Representative transmission electron microscopy (TEM) images of murine AT2 cells from whole lung mounts. Scale bars: 600 nm. (**H**) MitoTracker staining of murine AT2 cells after 18 hours in culture shows altered mitochondrial network morphology in AT2^I73T^ versus AT2^WT^ cells. Scale bars: 10 nm. (**I**) Quantification of mitochondrial structure in AT2 cells using ImageJ (NIH) Mitochondrial Analyzer shows increased fragmentation, decreased branch length, and altered shape in AT2^I73T^ cells (each point is an average of 1 field of view containing a minimum of 10 cells; cells were isolated and cultured from *n* = 4 mice per condition and 5–6 fields were quantified). (**J**) Western blot and densitometric quantification (*n* = 3 biological replicates) of mitochondrial dynamics proteins demonstrates altered regulation of dynamics and mitophagy in AT2^I73T^ cells. All mean± SEM. *P < 0.05, **P < 0.005, ***P < 0.0005, ****P < 0.00005 by ordinary 1-way ANOVA.

The shift toward glycolysis that emerged following induction of *Sftpc^I73T^* was accompanied by significant alterations to the mitochondrial phenotype. Gene set enrichment analysis (GSEA) of Hallmark gene signatures (43), based on the popRNA-Seq analysis, identified a significant downregulation of the PPAR signaling pathway in AT2^I73T^ cells (Figure 2A), a pathway critical in the regulation of mitochondrial biogenesis. Furthermore, the majority of genes encoding mitochondrially localized proteins (MitoCarta3.0 dataset) (44) were downregulated in AT2^I73T^ cells (Figure 2A). In support of this transcriptional signature, we observed a reduction in activated phosphorylated PGC1α (p-PGC1α) protein (Figure 2B) as well as a significant time-dependent reduction in mitochondrial DNA (mtDNA) copy number (Figure 2C), a marker of mitochondrial biogenesis (45, 46). Similarly, mtDNA gene expression was downregulated (Figure 2D) in AT2^I73T^ cells. In addition, we observed time-dependent collapse of mitochondrial membrane potential (ΔΨm) and reduced respiration (basal, maximal, and spare respiratory capacity). These changes were observed as early as 14 days after in vivo induction of mutant SP-C expression and coincided with increased extracellular acidification (Figure 2, E and F, and Supplemental Figure 2C).

Given that functional impairments in mitochondria (specifically ΔΨm alterations) are often matched by compensatory increases in mitochondrial clearance through mitophagy and that we have previously observed defects in autophagy and mitophagy in vitro (26, 27), we hypothesized that mitochondrial turnover could be compromised, resulting in a reorganized AT2 cell mitochondrial network. Electron microscopy demonstrated significant ultrastructural alterations to the mitochondrial network of AT2^I73T^ cells (Figure 2G) manifested as an accumulation of smaller mitochondria, reminiscent of prior observations made in AT2 cells derived from IPF lungs (37). Similarly, immunofluorescence (IF) microscopy of labeled mitochondria in isolated AT2 cells showed the presence of a disrupted mitochondrial network with more mitochondrial fragments per cell, decreased branch lengths, and altered fragment shape in AT2^I73T^ cells (Figure 2, H and I). Next, we tested whether there was a link between the accumulation of fragmented dysfunctional mitochondria and disrupted mitochondrial turnover (Figure 2J). We observed decreased protein levels of the mitophagy regulators PINK1 and Parkin that in tandem with the loss of p-DRP1 (Ser616) and MFF1 (Ser146) suggested a DRP1-independent fission mechanism (47). Of note, both DRP1 and MFF1 are targets of AMPK, with MFF1 (Ser146) recognized as a canonical AMPK phosphorylation motif (48, 49). Taken together, these findings suggest diminished mitochondrial biogenesis and disruptions in ΔΨm, mitochondrial respiration, and mitochondrial fission/fusion dynamics in AT2^I73T^ cells.

### Impaired AMPK signaling in murine AT2^I73T^ cells.

**Figure 3:**
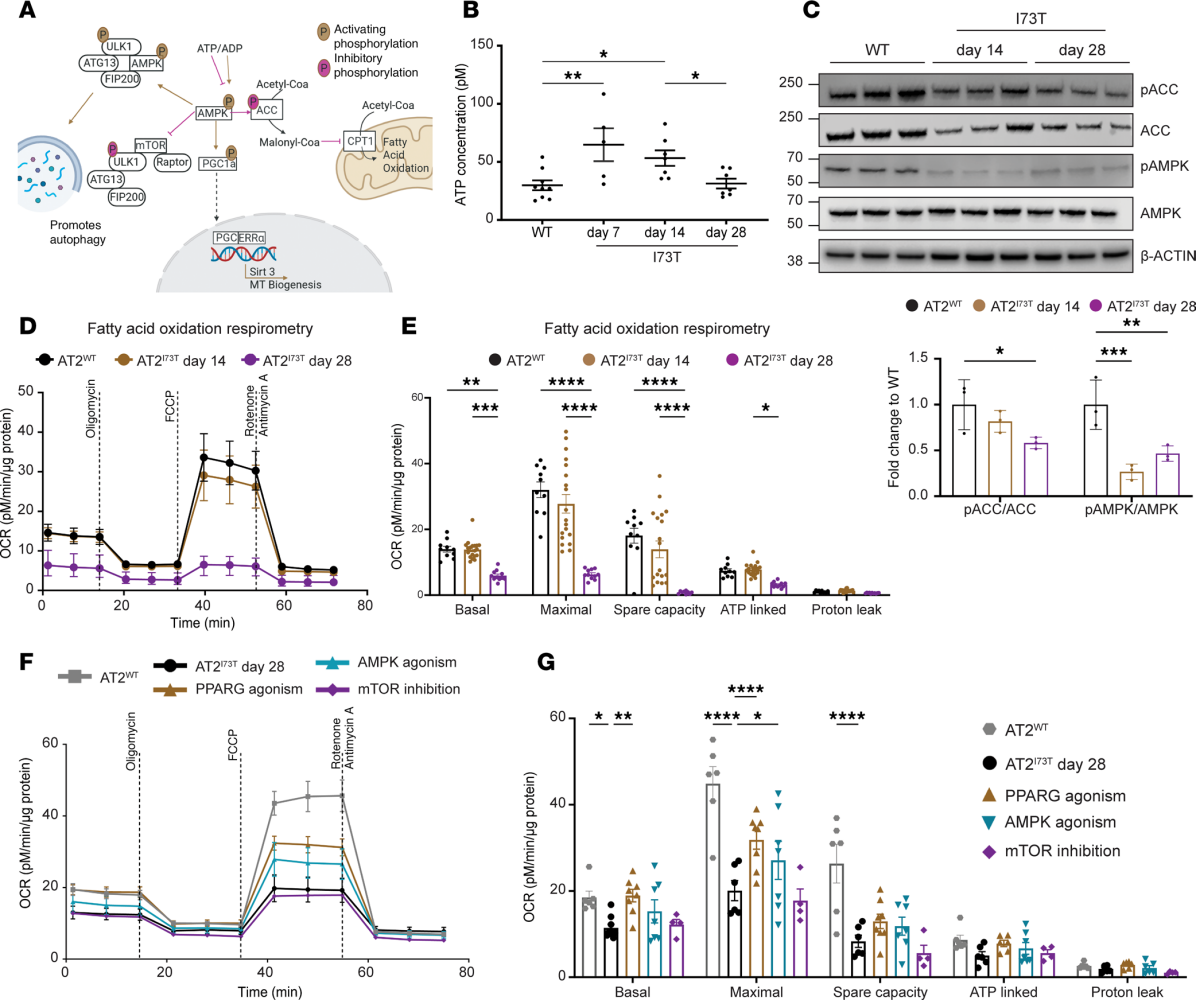
Impaired fatty acid oxidation and mitochondrial respiration are linked to AMPK signaling. (**A**) Graphical representation of AMPK as a key regulator of many cellular processes, including mitochondrial biogenesis, autophagy, and fatty acid oxidation (FAO). (**B**) Increased ATP accumulation in freshly isolated AT2^I73T^ cells at 7 days and 14 days after in vivo tamoxifen induction (mean± SEM; 25,000 cells, *n* = 5–9 mice per condition). (**C**) Western blot and densitometric quantification (mean± SEM; *n* = 3 biological replicates) of key enzymes in AMPK signaling pathway demonstrates reduced AMPK signaling and FAO in AT2^I73T^ cells. (**D** and **E**) Reduced OCR in AT2^I73T^ cells isolated at 14 days and 28 days after tamoxifen induction and cultured overnight in media supplied with endogenous fatty acids (mean± SEM; *n* = 9–19 mice per condition). (**F** and **G**) OCR in AT2^I73T^ cells isolated at 28 days after in vivo tamoxifen induction and cultured for 48 hours in the presence of the following small molecules: rosiglitazone (25 μM, *n* = 8) to stimulate PPAR-γ, PF-06409577 (100 nM, *n* = 7) to activate AMPK, and Torin 1 (100 nM, *n* = 4) to inhibit mTOR (mean±SEM). *P < 0.05, **P < 0.005, ***P < 0.0005, ****P < 0.00005 by ordinary 1-way ANOVA.

To gain a mechanistic understanding of the relationship between disrupted AT2 cell metabolism, diminished mitochondrial biogenesis, and impaired macroautophagy, we focused on ATP levels and AMPK activity, a well-characterized signaling hub regulating several cellular processes, including autophagy and mitochondrial homeostasis (Figure 3A) (50–53). In line with the well-documented Warburg effect, initially observed in tumor cells and subsequently identified in some progenitor cell populations (53, 54), AT2^I73T^ cells displayed an early surge in ATP levels sustained through day 14 postinduction, followed by a return to baseline levels at the fibrotic 28-day time point (Figure 3B). Next, we performed immunoblot analysis of AT2 cell lysates to interrogate AMPK signaling and its downstream regulation of FAO through the acetyl-CoA carboxylase (ACC) and observed decreased phosphorylation of AMPK with a concurrent decrease in ACC phosphorylation in AT2^I73T^ cells at 14 and 28 days after in vivo induction of mutant SP-C (Figure 3C).

To functionally assess the downstream consequences of disrupted AMPK signaling, particularly its influence on FAO, we analyzed the oxygen consumption of AT2 cells under minimal media conditions containing the long-chain fatty acid palmitate. We found significant time-dependent reductions in basal, maximal, and spare oxygen consumption rates (OCRs) in AT2^I73T^ cells when limited to substrates promoting oxygen consumption through FAO (Figure 3, D and E). These findings suggest that disrupted AMPK signaling not only stems from the altered metabolic phenotype but also may contribute to the dysfunction through regulation of downstream pathways, including fatty acid metabolism.

We next tested potential approaches for reversing the bioenergetic deficiencies in AT2^I73T^ cells by modulating AMPK or its downstream pathways (Figure 3, F and G). We isolated AT2 cells from wild-type and *Sftpc^I73T^* mice 28 days after in vivo tamoxifen induction (fibrotic time point). AT2^I73T^ and AT2^WT^ cells were cultured in 2D and treated with rosiglitazone to stimulate PPAR-γ, PF-06409577 to activate AMPK, or Torin 1 to inhibit mTOR. Cultured AT2^I73T^ cells accumulated processing intermediates of mutant pro–SP-C, retained increased levels of LC3B, and demonstrated decreased levels of p-PGC1α compared with AT2^WT^ cells (Supplemental Figure 2, A and B). Forty-eight hours of either direct PPAR-γ or AMPK activation in AT2^I73T^ cells rescued defective mitochondrial respiration with significant increases in maximal OCR compared with vehicle controls, whereas mTOR inhibition had no effect on OCR (Figure 3, F and G). Taken together, these findings suggest that the impaired AMPK signaling in AT2^I73T^ cells, associated with elevated ATP levels, results in defective FAO and mitochondrial respiration and that PPAR-γ activation or AMPK agonism effectively ameliorates the mitochondrial respiration defects.

### Diminished progenitor function and emergence of a transitional cell state resulting from Sftpc^I73T^ expression.

**Figure 4:**
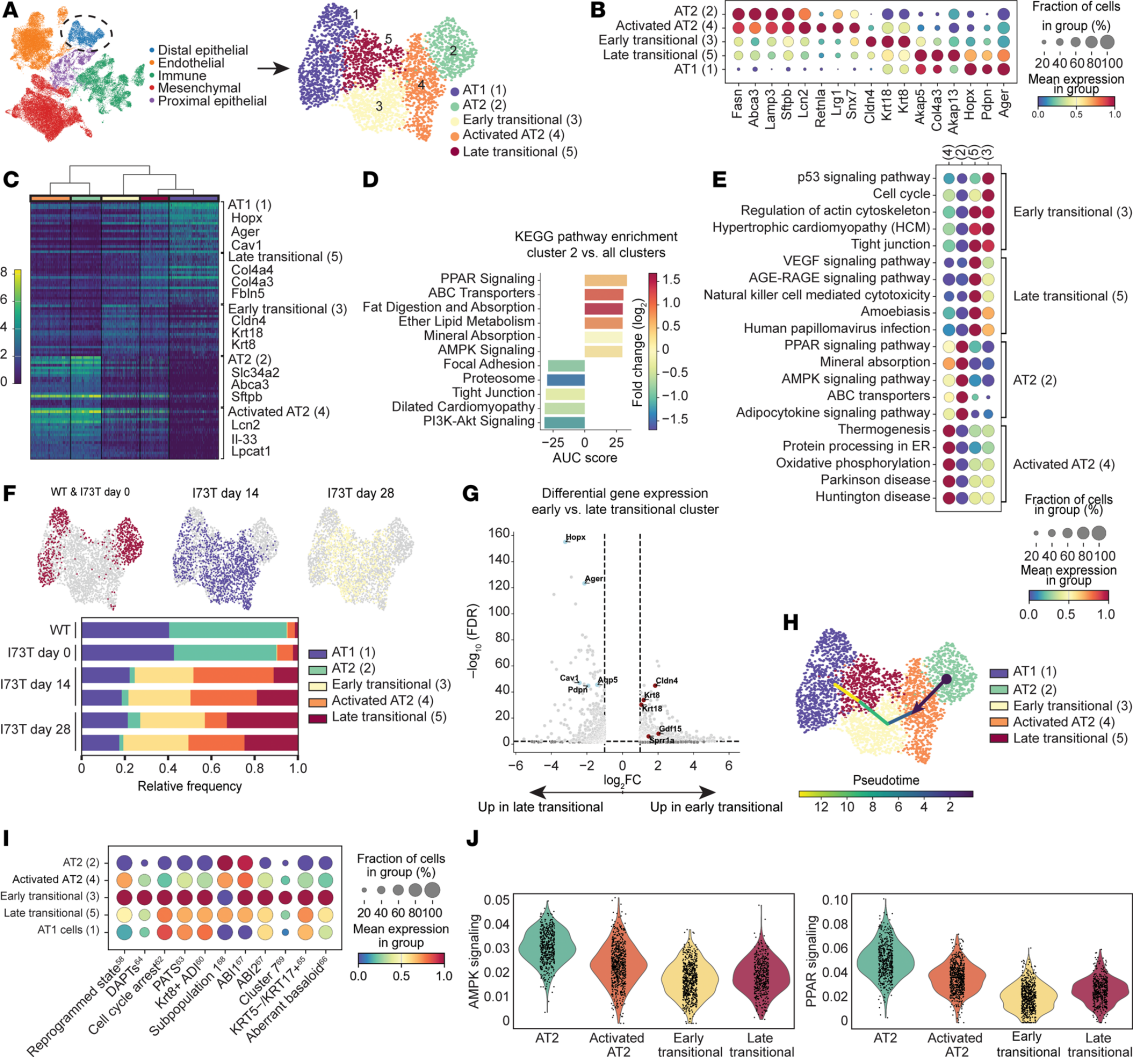
Metabolic alterations and emergence of an epithelial transitional state in response to in vivo *Sftpc^I73T^* expression. (**A**) Uniform manifold approximation and projection (UMAP) visualization of 35,002 lung cells profiled by scRNA-Seq in GSE234604 (56) color-coded by cell lineage with subset analysis of 2,500 distal epithelial cells. (**B**) Gradient dot plot of key distal epithelial genes used to annotate AT1 (cluster 1), AT2 (clusters 2, 4), and transitional subclusters (clusters 3, 5). (**C**) Dendrogram of top 20 DEGs and their associated log_2_FC for each cluster as determined by FDR. A subset of cluster-defining genes is highlighted. (**D**) Kyoto Encyclopedia of Genes and Genomes (KEGG) pathway enrichment analysis of DEGs (FDR < 0.05, log_2_FC > 1 & <–1) in cluster 2 compared with other clusters. (**E**) Gradient dot plot of KEGG pathway enrichment analysis of upregulated DEGs (FDR < 0.05, log_2_FC > 1) across clusters. (**F**) Color-coded UMAPs by genotype and time point and frequency table denoting cluster distributions within biological samples. (**G**) Volcano plot of differential expression analysis (FDR < 0.05, log_2_FC > 1 or <–1) comparing the early (cluster 3) and late (cluster 5) transitional clusters highlighting decreased expression of AT1 cell marker genes (*Hopx*, *Ager*, *Cav1*, *Pdpn*, *Aqp5*) and increased expression of transitional cell marker genes (*Cldn4*, *Krt8*, *Krt18*, *Gdf15*, *Sppr1a*) in the early transitional cell cluster. (**H**) Pseudotime trajectory analysis with starting node set in the AT2 cell cluster. (**I**) Gradient dot plot of indicated transitional state gene modules (Supplemental Table 1) (58, 60, 62–69) across distal epithelial clusters. (**J**) KEGG pathway module scores for AMPK and PPAR signaling demonstrate progressive downregulation from the AT2 cell cluster to transitional cell clusters.

In the intestinal and lung epithelium, metabolic alterations have been associated with a defect in epithelial progenitor cell function (27, 55). To further assess potential alterations in AT2^I73T^ progenitor cell function (self-renewal and differentiation to AT1 cells) due to the observed bioenergetic changes, we reanalyzed our recently published single-cell RNA-sequencing (scRNA-Seq) time series profiles (National Center for Biotechnology Information [NCBI] Gene Expression Omnibus [GEO] GSE234604) (56) of *Sftpc*^I73T^ mouse lungs (14 and 28 days after tamoxifen induction; Supplemental Figure 3). Within this dataset composed of 35,002 cells, 2,500 alveolar epithelial cells were identified and reclustered for further analysis (Figure 4A and Supplemental Figure 4). Louvain clustering identified 5 alveolar cell clusters. Based on top DEGs and published canonical markers (Figure 4, B–E, and Supplemental Figure 4B), 2 of these were annotated as AT2 cells (57–59) (cluster 2, which was the predominant AT2 cluster present in control mice, and cluster 4, which was the predominant AT2 cluster following tamoxifen induction; Figure 4F), 1 as AT1 cells (cluster 1), and 2 as putative transitional cells (clusters 3 and 5; detailed below). KEGG pathway analysis of the DEGs defining each cluster (Figure 4, C and D, and Supplemental Figure 4) identified PPAR signaling as the most upregulated pathway in cluster 2 AT2 cells (hereafter referred to as “AT2”), whereas cluster 4 AT2 cells, hereafter referred to as “activated AT2,” had lower PPAR signaling and higher expression of published AT2 activation markers, *Lcn2* and *Il-33* (60, 61). AT2 cells (cluster 2) were also distinguished from activated AT2 cells (cluster 4) by significantly higher AMPK signaling and mitophagy (Supplemental Figure 4, H and I).

In lung injury models, disrupted AT2-to-AT1 differentiation was recently linked to the emergence of a putative “transitional state,” characterized by a gene signature that in mice includes *Krt8* and *Cldn4* (58, 60, 62). Given that clusters 3 and 5 were enriched in transitional state markers, including *Krt8*, *Krt18*, *Sox4*, *Fn1*, and *Cldn4* (Figure 4B), we annotated these as transitional cells. The relative abundance of cluster 3 remained unchanged between day 14 and day 28, whereas cluster 5 was more abundant in day 28 (Figure 4F). Comparing cluster 3 with cluster 5, we observed differential upregulation of canonical AT1 cell marker genes (*Hopx*, *Pdpn*, *Ager*, *Cav1*, *Aqp5*) in cluster 5 (Figure 4G). Furthermore, pseudotime analysis using a starting node in the AT2 cluster suggested that cluster 3 serves as an early precursor state to cluster 5 (Figure 4H and Supplemental Figure 4). Based on these observations, we chose to refer to cluster 3 as “early” and cluster 5 as “late” transitional cluster.

To further contextualize the 2 putative transitional states, we next defined transitional cell gene modules based on recently published datasets of diseased mouse or human lungs that included scRNA-Seq profiles of these cells, variably referred to as pre-alveolar type-1 transitional cell state (PATS) (63), cell cycle arrest (62), damage-associated transient progenitors (DATPs) (64), KRT5^−^KRT17^+^ (65), aberrant basaloid (66), Krt8^+^ alveolar differentiation intermediate (ADI) (60), alveolar-basal intermediate (ABI1/2) (67), subpopulation 1 (68), cluster 7 (69), or reprogrammed state (58) (Supplemental Table 1). Of all our annotated cell clusters, we found early transitional cells (cluster 3) had the highest expression of all transitional cell modules defined from prior reports, irrespective of the injury model or species (Figure 4I). Furthermore, using AMPK signaling and PPAR signaling pathway gene modules, we observed progressively decreased expression of these pathways as AT2^I73T^ cells entered the transitional state (Figure 4J).

**Figure 5:**
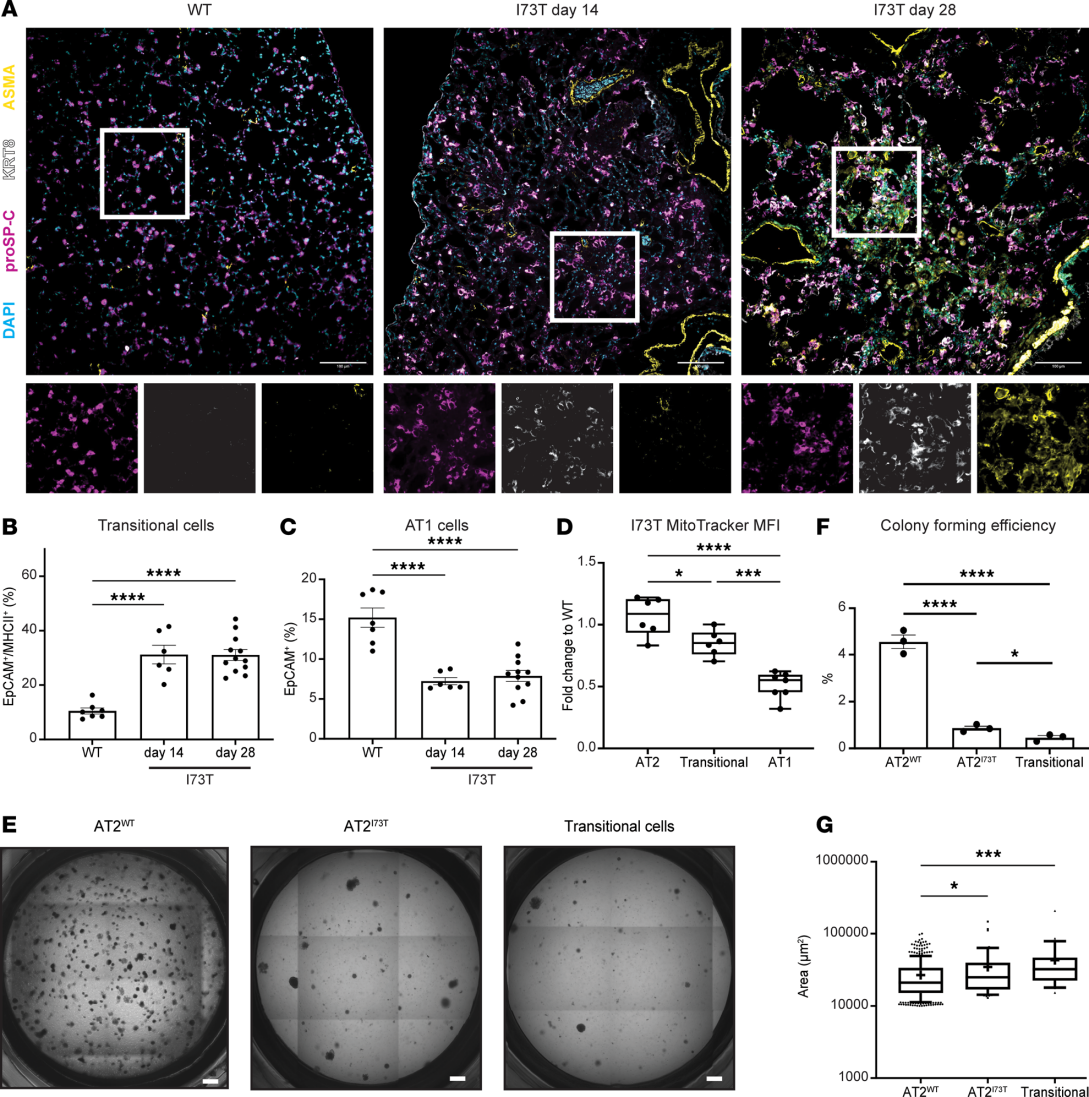
Persistence of an epithelial transitional state with diminished progenitor capacity in *Sftpc^I73T^* lungs. (**A**) Representative immunofluorescence staining of murine lung sections from WT or *Sftpc^I73T^* mice 14 days and 28 days after tamoxifen induction stained with antibodies against pro–SP-C, KRT8, ASMA, and DAPI for nuclei counterstaining. Scale bars: 100 μm. (**B**) Flow cytometry quantification of CD51^+^ transitional cells shows a sustained increase of transitional cells after in vivo tamoxifen induction (mean±SEM; *n* = 6–12 mice per time point). (**C**) Flow cytometry quantification of EpCAM^+^CD51^–^CD104^–^ AT1 cells demonstrates a sustained loss of AT1 cells after in vivo tamoxifen induction (mean±SEM; *n* = 6–12 mice per time point). (**D**) MFI of MitoTracker dye accumulating in AT2, transitional, and AT1 cells isolated from *Sftpc^I73T^* mice at 14 days after tamoxifen induction (mean± SEM; *n* = 6–9 mice per time point). Box plots show the interquartile range, median (line), and minimum and maximum (whiskers). (**E**) Representative light microscopy images of 21-day organoid cultures derived using WT PDGFRα^+^ fibroblasts and 1) AT2 ^WT^ cells, 2) AT2^I73T^ cells, and 3) transitional cells isolated from *Sftpc^I73T^* mice at 14 days after tamoxifen induction. Scale bars: 500 μm. (**F**) CFE of organoids with surface area > 10,000 μm^2^ (mean±SD; *n* = 3 biological replicates per condition). (**G**) Surface area quantification of organoids with surface area > 10,000 μm^2^ (each point represents an individual organoid mean±SEM; *n* = 3 biological replicates per condition). *P < 0.05, ***P < 0.0005, ****P < 0.00005 by ordinary 1-way ANOVA.

Next, we used IF microscopy on paraffin-embedded sections from *Sftpc*^I73T^ mouse lungs at 14 and 28 days after tamoxifen induction of mutant SP-C^I73T^, as well as WT mouse lungs, to localize KRT8^hi^ cells. Consistent with a recent study in a model of bleomycin lung injury (69), we found the emergence of KRT8^hi^ pro–SP-C coexpressing cells *prior to* widespread fibrogenesis (day 14) and the persistence of that cell population through the fibrotic stage (day 28), where KRT8^hi^pro–SP-C^+^ cells localized to areas of alpha–smooth muscle actin–positive (ASMA^+^) injury (Figure 5A). To verify and quantify the increase in transitional cells, we applied our recently developed flow cytometry approach employing CD51 (ITGAV) to sort transitional cells (70) (Supplemental Figure 5, A and B). Since this method was initially applied in an infection model, we further validated its applicability in this context by performing RNA-Seq on CD51^+^ and CD51^–^ cells from *Sftpc*^I73T^ mouse lungs and WT AT2 cells. The transcriptomic profiles verified that CD51^+^ cells were enriched for transitional marker genes (Supplemental Figure 5C) previously identified in our single-cell analysis (Figure 4). These findings were consistent with our IF staining results and demonstrated a progressive increase in transitional cells during fibrosis development (Figure 5B). Furthermore, flow cytometry analysis verified the loss of AT1 cells (Figure 5C) suggested by the scRNA-Seq quantification (Figure 4F). Single-cell suspensions of AT2, transitional, and AT1 cells isolated from *Sftpc*^I73T^ mouse lungs 14 days after in vivo tamoxifen induction revealed a graded reduction in MitoTracker fluorescence from AT2 to transitional to AT1 cells, indicating loss of mitochondrial mass during the AT2-to-AT1 differentiation (Figure 5D), consistent with AT2 gene expression profiles and altered mitochondrial content (Figure 2).

We assessed the capacity of AT2^I73T^ cells to self-renew, a measure of progenitor potential. We established 3D organoid cultures using WT PDGFRα^+^ fibroblasts and 1) AT2 cells isolated from WT mice, 2) AT2^I73T^ cells, and 3) transitional cells (Supplemental Figure 5) isolated from *Sftpc^I73T^* mice 14 days after in vivo tamoxifen induction. After 14 days in culture, colony-forming efficiency (CFE) was determined (Figure 5E). We observed a significant decrease in CFE for AT2^I73T^ and transitional cells compared with AT2^WT^ cells, with transitional cells exhibiting the lowest CFE (Figure 5F). This was consistent with our previous findings in human patient-specific *SFTPC^I73T^*-expressing iAT2s (27). Although AT2^I73T^ and transitional cells formed fewer organoids, these were larger than AT2^WT^ cell–derived organoids (Figure 5G). Collectively these data support diminished AT2 cell progenitor capacity because of mutant SP-C^I73T^ expression, evident as reduced AT2 self-renewal potential coincident with reduced presence of differentiated AT1 cells and accumulation of transitional cells.

### AMPK agonism ameliorates metabolic alterations observed in human iPSC-derived AT2^I73T^ cells.

**Figure 6:**
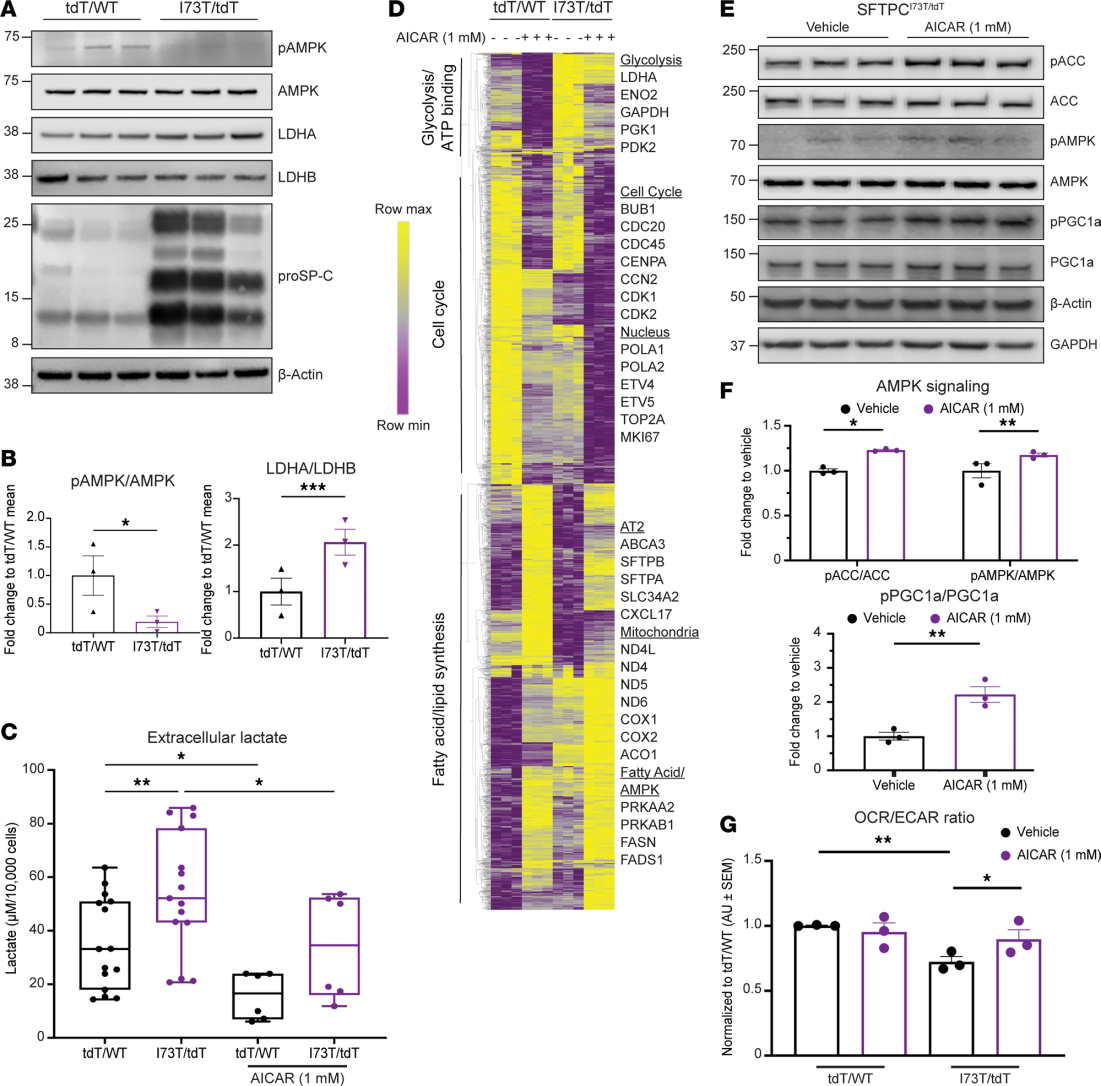
AMPK agonism ameliorates the metabolic alterations observed in human iAT2^I73T^ cells. (**A** and **B**) Western blot and densitometry quantification (normalized to loading control and presented as FC over iAT2^WT^ of serially cultured human iAT2 cells (135–182 days) demonstrate increased LDHA/B ratio and decreased p-AMPK/AMPK ratio in iAT2^I73T^ (*SFTPC^I73T/tdTomato^*) cells compared with syngeneic corrected iAT2^WT^ cells (*SFTPC^WT/tdTomato^*) (mean±SEM; *n* = 3 biological replicates). (**C**) Measurement of extracellular lactate by YSI biochemistry analyzer shows increased extracellular lactate in iAT2^I73T^ compared with iAT2^WT^ cells. This increase is significantly reduced by treatment with AICAR (1 mM, 24 hours) (mean±SEM; *n* = 3 biological replicates for AICAR-treated and *n* = 5 biological replicates for vehicle-treated, each with 2 experimental replicates of independent wells). (**D**) Unsupervised hierarchical clustering (Euclidean) heatmap of all DEGs (FDR < 0.05) in iAT2^WT^ and iAT2^I73T^ cells treated with AICAR treatment (1 mM, 24 hours) or vehicle (row-normalized *z*-score). Gene ontology (GO) analysis of each subgroup using Database for Annotation, Visualization, and Integrated Discovery (DAVID) identifies increased expression of genes associated with fatty acid and lipid synthesis and reduced expression of transcripts linked to cell cycle regulation and glycolysis after AICAR treatment. A subset of sample genes from each GO term is highlighted. (**E** and **F**) Western blot of AMPK pathway targets in iAT2^I73T^ cell lysates verifies AMPK signaling activation and increased PGC1α phosphorylation following AICAR treatment (1 mM, 24 hours). Bar graphs depict densitometric quantification (mean±SEM; *n* = 3 biological replicates). (**G**) Respirometry quantification depicted as OCR/ECAR ratio in iAT2^I73T^ and iAT2^WT^ cells following AICAR (1 mM, 24 hours) treatment (mean±SEM; *n* = 3 biological replicates). *P < 0.05, **P < 0.005, ***P < 0.0005, by ordinary 1-way ANOVA (**C** and **G**) and 1-tailed unpaired *t* test (**B** and **F**).

To test whether human AT2 cells expressing SP-C^I73T^ exhibit similar perturbations, we used iAT2s (71), a preclinical in vitro platform of human AT2 cell dysfunction that enables testing of epithelial intrinsic effects without the secondary effects arising from other cell lineages. We previously reported the generation of iPSCs from patients with *SFTPC^I73T^* variants and lung disease (27) and identified that expression of mutant SP-C^I73T^ in iAT2s results in diminished AT2 cell progenitor function, autophagy perturbations, altered bioenergetic programs, time-dependent increased glycolysis, and activated canonical NF-κB signaling (27). Consistent with our previous work (27), popRNA-Seq analysis of iAT2^I73T^ cells versus syngeneic gene-edited iAT2^WT^ cells expressing normal (WT) *SFTPC* paralleled the changes identified in murine AT2^I73T^ cells (Figure 1, A–D) with significant changes to multiple metabolic pathways (Supplemental Figure 6, A–C). Further reinforcing the previous findings and supporting the reproducibility of this preclinical model, we observed changes to cytokine signaling, cell cycle, and extracellular matrix–associated transcripts (Supplemental Figure 6, D–G). Immunoblot analysis of iAT2^WT^ versus iAT2^I73T^ cells revealed a quantitative increase in LDHA/LDHB and a decrease in AMPK phosphorylation in iAT2^I73T^ cells (Figure 6, A and B). Similar to mouse AT2^I73T^ cells (Figure 1) and again consistent with our recently reported increase in extracellular acidification rate (ECAR) (27), human iAT2^I73T^ cells demonstrated increased lactate production (Figure 6C).

Given that AMPK activation in mouse AT2^I73T^ cells in vitro alleviated the mitochondrial respiration defects (Figure 3, F and G), we sought to determine whether the increased glycolysis and reduced mitochondrial biogenesis observed in human iAT2^I73T^ cells could be similarly ameliorated through direct AMPK stimulation (Supplemental Figure 7A). We treated iAT2^I73T^ cells with AMPK agonist acadesine (AICAR) 1 mM for 24 hours, a dose and duration verified to stimulate mitochondrial biogenesis and autophagy, 2 downstream targets of AMPK signaling (Supplemental Figure 7, A–D). AICAR treatment led to a reduction in extracellular lactate levels in both mutant and corrected iAT2s (Figure 6C), consistent with a reduction in glycolytic shift while correcting the previously described defect in NF-κB signaling (27) (Supplemental Figure 7E). RNA-Seq analysis of iAT2^WT^ and iAT2^I73T^ cells treated with AICAR or vehicle (Supplemental Figure 8, A and B) identified 2,351 differentially expressed transcripts. Despite differences in specific genes between mutant and corrected cells (Supplemental Figure 8C), pathway enrichment analysis revealed genotype-independent effects of AICAR, including upregulation of autophagy and lipid metabolism pathways and downregulation of mitotic cell cycle and glycolytic processes (Supplemental Figure 8, D–G). Unbiased heatmap analysis of DEGs highlighted increased expression of transcripts involved in fatty acid and lipid synthesis, including those related to surfactant homeostasis, such as *ABCA3* and *SFTPB*, following AICAR treatment (Figure 6D and Supplemental Figure 8H). Conversely, transcripts related to cell cycle and glycolysis, including *LDHA*, were decreased (Figure 6D and Supplemental Figure 8H). Immunoblot analysis further verified that AMPK agonism via AICAR increased PGC1α phosphorylation and enhanced mitochondrial biogenesis, evidenced by the upregulation of multiple PGC1α target genes (Figure 6, D–F). Functionally, the improved mitochondrial biogenesis observed in human iAT2^I73T^ cells following AICAR treatment was accompanied by an enhanced OCR/ECR ratio driven largely by increased maximal mitochondrial respiration (Figure 6G and Supplemental Figure 7, F–H). Collectively, these findings suggest that direct AMPK agonism in human iAT2^I73T^ cells through AICAR enhances mitochondrial biogenesis and ameliorates the observed mitochondrial respiration defects.

### AMPK agonism promotes AT2 cell respiration in vivo and rescues the fibrotic Sftpc^I73T^ lung phenotype.

**Figure 7:**
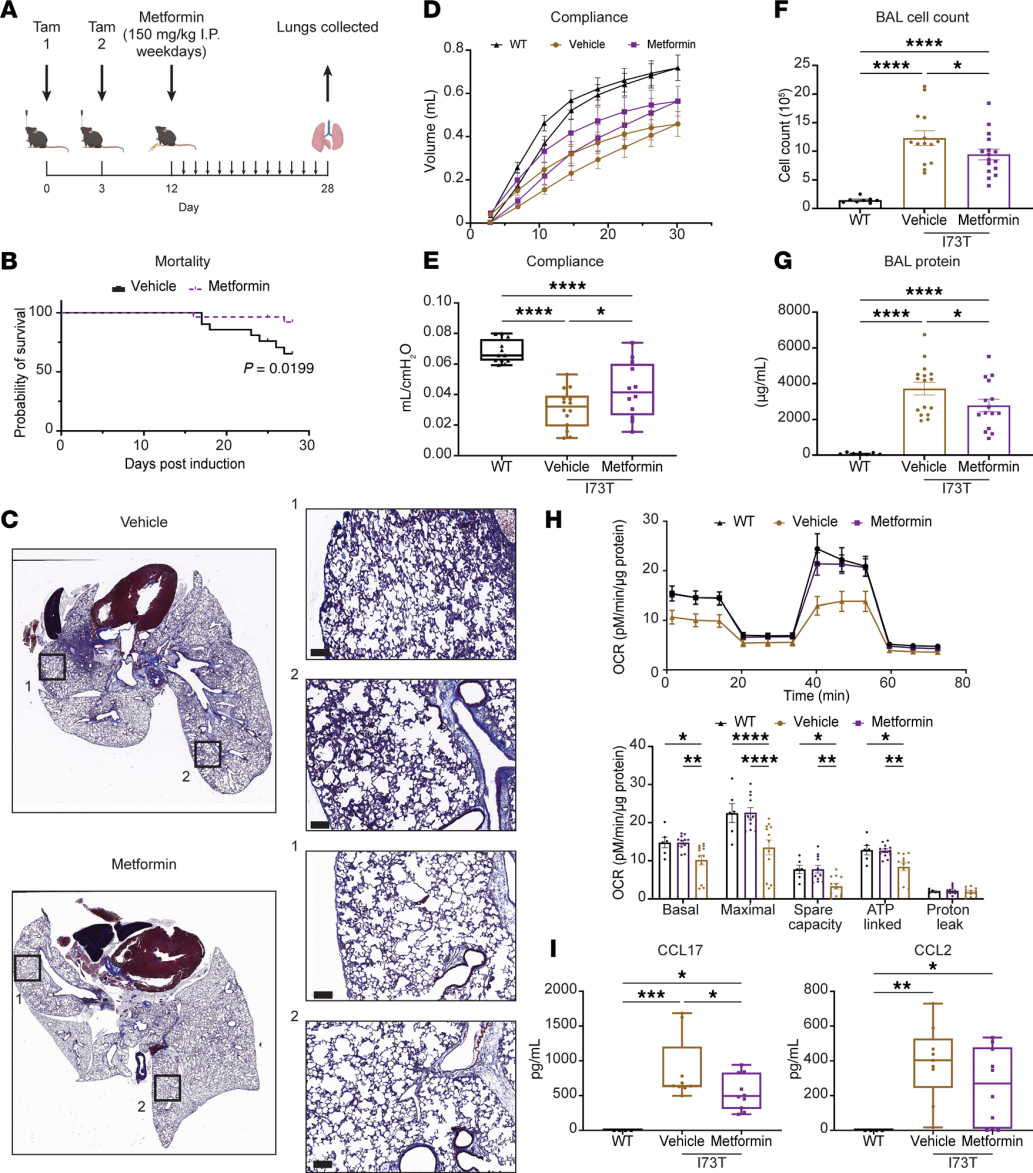
In vivo rescue of the *Sftpc^I73T^* fibrotic phenotype via metformin intervention. (**A**) Study design for metformin intervention in the *Sftpc^I73T^* fibrosis mouse model. Metformin (150 mg/kg) or vehicle control was administered intraperitoneal (i.p.) daily on weekdays starting at 12 days after tamoxifen induction (*n* = 20 mice per group). (**B**) At 28 days, mortality was significantly (*P* = 0.0199) reduced in metformin-treated mice. (**C**) Representative Masson’s trichrome staining of *Sftpc^I73T^* mouse lungs treated with either metformin or vehicle control and collected at 28 days after tamoxifen induction. (**D** and **E**) Static lung compliance, measured by SCIREQ Flexivent, increased significantly in metformin-treated mice compared with vehicle control (mean±SEM; *n* = 14 mice for vehicle group and *n* = 12 for WT and metformin groups). (**F** and **G**) Markers of inflammation and lung injury in the bronchoalveolar lavage fluid (BALF) collected from mice at 28 days after tamoxifen were significantly reduced in metformin-treated mice (mean±SEM; *n* = 12 mice per group). (**H**) OCR measurement and quantification in AT2 cells isolated from WT and *Sftpc^I73T^* mice at 28 days after in vivo tamoxifen induction. AT2 cells were seeded overnight before respirometry was performed (mean±SEM; *n* = 8 mice per group). (**I**) ELISA quantification of CCL17 and CCL2 concentrations in BALF of WT and *Sftpc^I73T^* mice at 28 days after in vivo tamoxifen induction (mean±SEM; *n* = 4 WT, 9 vehicle, and 10 I73T mice). *P < 0.05, **P < 0.005, ***P < 0.0005, ****P < 0.00005 by ordinary 1-way ANOVA.

Given that AMPK agonism reversed the AT2^I73T^ cell metabolic phenotype in both mouse and human cells in vitro, we next sought to test whether a similar strategy could reverse the observed metabolic changes in vivo as a therapeutic approach. In a clinically relevant interventional dosing strategy (Figure 7A), metformin administration (150 mg/kg given intraperitoneally, 5 days per week) initiated on day 12 after tamoxifen induction of mutant SP-C^I73T^ expression significantly improved survival from less than 70% in the vehicle-treated mice to 90% in the intervention arm (*P* = 0.0199, Figure 7B). Gross histological examination of lung structure in surviving mice at 28 days revealed a decreased cellular infiltrate and improved alveolar architecture in metformin-treated mice (Figure 7C). Quantitatively, these changes were reflected in improved static lung compliance (Figure 7, D and E) and reduced total cell counts and protein levels in the bronchoalveolar lavage fluid (BALF) (Figure 7, F and G). The effect size of these metrics is comparable to our previously reported efficacy of nintedanib (BIBF1120) in the *Sftpc*^I73T^ mouse model (56).

To test whether the observed improvement in the fibrotic phenotype was linked to restored AT2 cell metabolic function, we isolated AT2^I73T^ cells from *Sftpc^I73T^* mice treated with either vehicle or metformin at day 28 after in vivo tamoxifen induction and evaluated OCR. Consistent with the in vitro findings (Figure 3, F and G), in vivo AMPK agonism enhanced AT2 cell mitochondrial respiration, improving basal, maximal, ATP-linked, and spare respiratory capacity (Figure 7H). Metformin treatment also reduced BALF levels of CCL17 and CCL2, cytokines we previously reported as being upregulated in AT2^I73T^ cells and increased in the *Sftpc^I73T^* mouse model of lung fibrosis (18) (Figure 7I). These findings indicate that metformin treatment in vivo enhances AT2 cell mitochondrial function and ameliorates lung fibrosis in the *Sftpc^I73T^* mouse model, potentially through the AMPK agonism that is known to be induced by metformin in other systems (72, 73).

**Figure 8:**
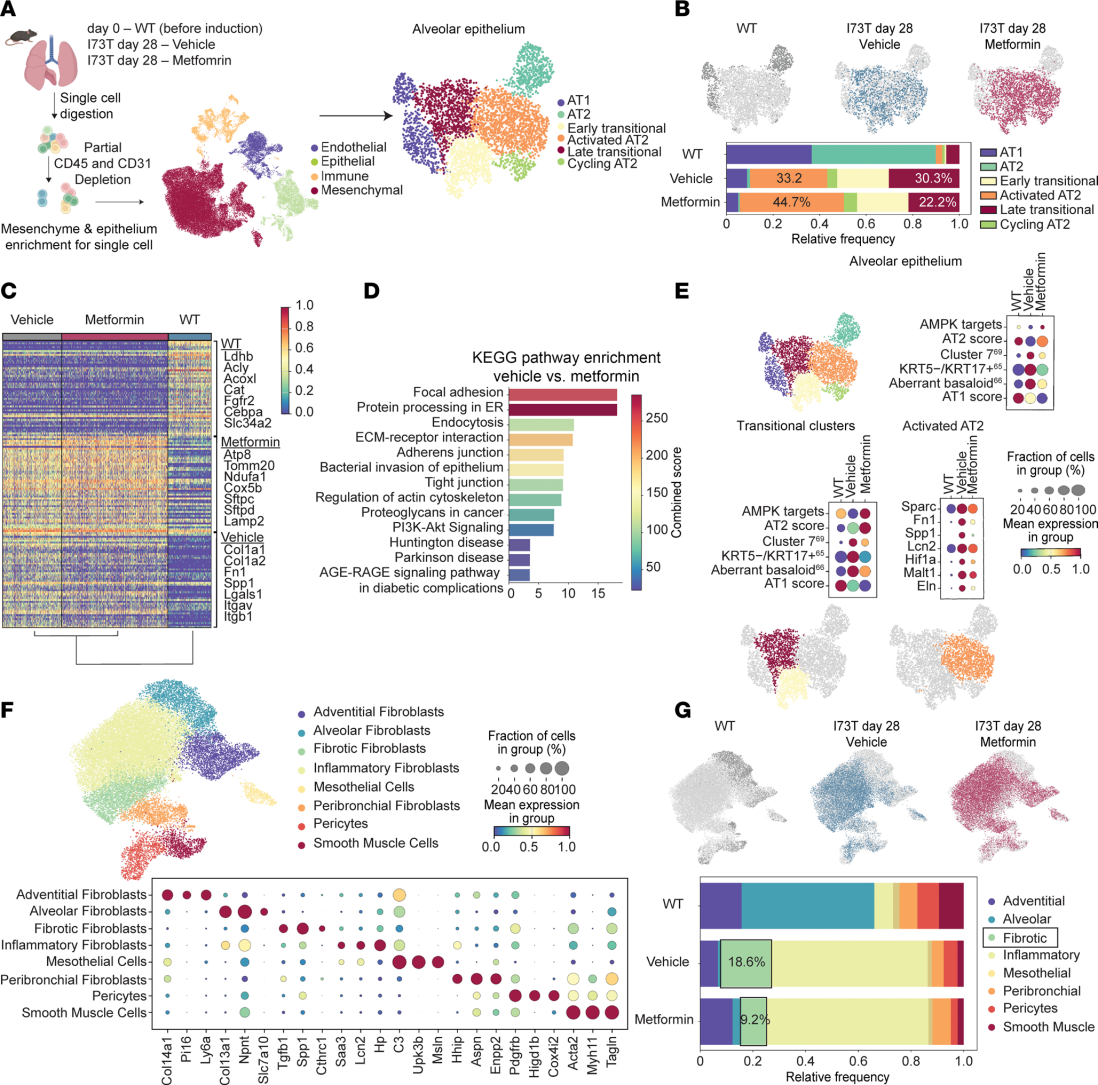
Metformin enhances AT2 cell transcriptional program and reduces fibrotic fibroblast population. (**A**) Schematic of experimental design and UMAP visualization of 39,327 lung cells from WT (age-matched C57B6/J; *n* = 2) and *Sftpc^I73T^* mice, treated with either metformin (*n* = 1) or vehicle control (*n* = 1), collected at 28 days after tamoxifen induction, profiled by scRNA-Seq, and color-coded by cell lineage. Subset analysis of 4,040 distal epithelial cells is shown. (**B**) UMAPs color-coded by genotype and time point and frequency table showing the distribution of each cluster within treatment groups. (**C**) Dendrogram of top 50 DEGs and their associated log_2_FC for each cluster as determined by FDR. A subset of cluster-defining genes is highlighted. (**D**) KEGG pathway enrichment analysis of DEGs (FDR < 0.05, log_2_FC > 1 and <–1) comparing vehicle versus metformin treatment. (**E**) Gradient dot plots demonstrating expression of select gene modules (Supplemental Table 1) in alveolar epithelium and transitional cell clusters and fibrosis-associated genes in activated AT2 cells. (**F**) Subclustering of the mesenchymal compartment (24,853 cells) identifies 8 mesenchymal clusters, defined by marker genes, depicted in a gradient dot plot. (**G**) UMAPs color-coded by genotype and time point, with a frequency table highlighting a decrease in the fibrotic fibroblast population after metformin treatment.

To investigate metformin’s impact on the transcriptomic profile of the alveolar epithelium, we performed scRNA-Seq on *Sftpc^I73T^* mice at 28 days after tamoxifen induction, treated with either vehicle or metformin, comparing them with WT controls. Given our specific interest in the alveolar epithelium and the previously reported effects of metformin on fibroblasts (32), we partially depleted endothelial and immune cells prior to profiling 39,327 cells via scRNA-Seq (Figure 8A). As before, we identified all 4 major cellular compartments (Supplemental Figure 9, A–C). Focusing on the alveolar epithelium and using the same marker genes to annotate epithelial populations (Figure 4B), we observed an increased frequency of activated AT2 cells and a decreased frequency of late transitional cells following metformin treatment (Figure 8B). Analysis of the top 50 DEGs in the alveolar epithelial compartment across the 3 conditions (WT, vehicle, and metformin) revealed upregulation of FAO-related genes (*Acly* and *Acoxl*) and *Ldhb* in WT epithelium (Figure 8C), reinforcing the role of FAO in AT2 cell homeostasis and the importance of its disruption after *Sftpc*^I73T^ expression. Metformin treatment increased alveolar epithelial expression of AT2-specific genes (*Sftpc*, *Sftpd*) and mitochondrial genes (*Tomm20*, *Ndufa1*), while reducing the expression of transitional cell markers (*Fn1*, *Spp1*, *Lgals1*, *Itgav*) (Figure 8C). KEGG pathway enrichment analysis of alveolar epithelial gene expression identified focal adhesion, extracellular matrix receptor interaction, and tight junction signaling among the top downregulated pathways in response to metformin (Figure 8D). The changes in cell frequencies induced by metformin were accompanied by expected transcriptional changes across the alveolar epithelial compartment, including decreased expression of transitional cell module scores and increased expression of AT2 cell and AMPK target gene modules (Figure 8E). To further examine the effect of metformin on specific clusters with altered frequencies — transitional cells and activated AT2 cells — we analyzed these subsets individually, pooling the 2 transitional clusters (Figure 8E). In the transitional clusters, metformin treatment increased AT2 cell and AMPK target gene module expression while decreasing transitional cell module expression (Figure 8E). Similarly, activated AT2 cells displayed reduced expression of pro-fibrotic genes, including *Spp1* and *Fn1*. Finally, the antifibrotic effect of metformin was further supported by a reduction in *Cthrc1^+^* fibrotic fibroblasts within the mesenchyme of metformin-treated mice (Figure 8, F and G). Taken together, these findings suggest that metformin exerts its antifibrotic effects by promoting AT2 cell identity, restoring the AT2 cell metabolic phenotype, suppressing aberrant epithelial populations, and reducing the fibrotic fibroblast population.

## Discussion

Through in vitro and in vivo models, we identified 4 key metabolic alterations in pulmonary fibrosis–associated mutant AT2^I73T^ cells: increased glycolysis, impaired mitochondrial biogenesis, disrupted mitochondrial respiration, and decreased FAO. We further identified AMPK as a central metabolic signaling hub that is deficient in AT2^I73T^ cells and found AMPK activation not only restored these metabolic alterations but also mitigated lung fibrosis in *Stfpc^I73T^* mice. Collectively, our results highlight a role of epithelial metabolism in distal lung homeostasis and its potential contribution to pulmonary fibrosis pathogenesis.

Early studies on metabolic dysfunction in IPF focused primarily on the mesenchymal compartment, particularly the role of lactate in fibroblast activation (29, 74). Inhibiting LDHA was shown to limit fibrotic endpoints in vitro and in preclinical in vivo models (74, 75). In our study, we observed increased lactate production in murine AT2^I73T^ and human iAT2^I73T^ cells, along with changes in LDH subunit composition, findings consistent with prior ex vivo observations in AT2 cells from end-stage IPF lungs (76). The generation of lactate observed in our models under aerobic conditions (Figure 1E and Figure 6C), coupled with altered transcription of glycolytic enzymes (Figure 1C), increased glucose flux (Figure 1E), changes in FAO (Figure 3), impaired oxidative phosphorylation (Figure 2F), and intracellular ATP accumulation (Figure 3B), collectively support the concept of a noncancerous Warburg effect as an important regulator of cell state (54, 77). The maintenance of the alveolar epithelial integrity, whether during homeostasis or in response to injury, relies on effective AT2 cell progenitor functions of self-renewal and differentiation into AT1 cells. Our findings indicate that AT2^I73T^ cells exhibit impaired mitochondrial respiration (Figure 2F and Figure 6G) and increased glycolysis (Figure 1, C–E, and Figure 6, A–C) consistent with a pathological metabolic state and diminished progenitor function, both in vivo (Figure 4F and Figure 5, A–C) and in vitro (Figure 5, E and F, and Alysandratos et al., ref. 27). A link between disrupted epithelial cell respiration and pulmonary fibrosis has been previously reported in *Pink1^–/–^* mice exhibiting impaired bioenergetics and age-related spontaneous fibrosis (37, 40). In 2 mouse models of pulmonary fibrosis, thyroid hormone administration improved mitochondrial biogenesis and bioenergetics, leading to amelioration of pulmonary fibrosis, an effect mediated through PGC1α and PINK1 (40). Similarly, genetic disruption of mitochondrial networks has been associated with impaired epithelial energy production and the promotion of spontaneous fibrosis (38). However, the precise mechanisms by which disruptions in cellular bioenergetics and metabolism affect AT2 cell function, ultimately leading to pulmonary fibrosis, remained elusive.

Our findings provide a mechanistic link between an aberrant AT2 cell metabolic phenotype and fibrogenesis as the impaired AMPK signaling in AT2^I73T^ cells (Figure 3) correlated with diminished AT2^I73T^ cell progenitor capacity, evident as reduced AT2 cell self-renewal in organoid models coincident with reduced presence of AT1 cells and accumulation of epithelial transitional cells in areas of fibrosis in the *Sftpc^I73T^* mouse model (Figures 4 and 5). The identification of a transitional cell state in multiple lung injury models (58, 60, 63–66, 68, 78) has raised fundamental questions regarding its role in disease pathogenesis, and our findings suggest that metabolic dysfunction may play a role in the emergence of this aberrant epithelial state.

Our scRNA-Seq analysis further reinforced the role of mitochondrial biogenesis and AMPK signaling (Figure 4J) in regulating epithelial homeostasis, prompting us to explore the therapeutic potential of AMPK agonism in pulmonary fibrosis. Previous studies have recognized AMPK as a druggable target for metabolic intervention in fibroblasts (32, 79–81). Furthermore, stimulating mitochondrial biogenesis and enhancing mitochondrial respiration in AT2 cells has been shown to improve outcomes in mouse models of lung fibrosis (34, 40, 82, 83). To explore this therapeutic potential in IPF, we harnessed the iAT2^I73T^ cell platform (27) for both target identification and validation. Leveraging the reductionist nature of this model, we first validated the altered glycolytic phenotype and consequent increased lactate production (Figure 6, A–C). Given that the inherent defect in this model stems from the autonomous expression of mutant SP-C^I73T^, we infer that the observed metabolic phenotype is a direct consequence of endogenous mutant protein expression rather than a secondary response to “outside-in” signaling. Utilizing this model, we also identified that AMPK agonism promoted key aspects of AT2 cell homeostasis, including fatty acid/lipid synthesis and mitochondrial biogenesis while stimulating mitochondrial respiration (Figure 6, D–G). As FAO has come to light as an important regulator of AT2 cell progenitor capacity (84), our findings underscore AMPK’s central role as a metabolic regulator of AT2 cell function and highlight the therapeutic potential of targeting multiple metabolic pathways simultaneously, laying the foundation for the in vivo application of AMPK agonism.

To translate these findings in vivo, we chose metformin, an indirect AMPK activator with reliable pharmacokinetics in mice. Metformin is FDA approved for the treatment of metabolic disorders (85), and emerging epidemiological evidence suggests clinical benefits in IPF (86). Consistent with prior data generated in a bleomycin model of pulmonary fibrosis (32), we observed significant improvements in survival, lung physiology, and markers of injury and immune infiltration following metformin treatment (Figure 7, B–G). A deeper analysis of lung epithelial and mesenchymal populations using epithelial/mesenchymal-enriched scRNA-Seq revealed that metformin exerts dual compartment effects (Figure 8). Mirroring observations in the iAT2 cell platform, metformin treatment increased the expression of AT2 homeostatic cell transcripts expanding the size of the activated AT2 cluster (Figure 8, B–E), while downregulating transcripts associated with the aberrant transitional epithelial cell state (Figure 8, D and E). Moreover, within the activated AT2 cluster, we observed reduced expression of transitional cell markers and pro-fibrotic genes following metformin treatment (Figure 8E), suggesting that although these cells may not fully revert to a homeostatic transcriptomic program, metformin ameliorates their dysfunctional phenotype. Supporting the notion that activated AT2 cells are less aberrant than transitional cells, they demonstrate superior progenitor capacity compared with transitional cells (Figure 5E). Although AT2^I73T^ are unable to adopt a fully homeostatic state due to persistent mutant isoform expression after tamoxifen induction and given that additional pathways beyond AMPK are affected, the expansion of less aberrant activated AT2 cells likely represents a beneficial outcome of metformin intervention. Concurrently, metformin reduced the size of the *Cthrc1*^+^ fibrotic fibroblast cluster, validating antifibrotic effects in at least 2 lung compartments. Taken together, our findings affirm the efficacy of AMPK agonism in rescuing AT2 cell metabolism and mitigating aberrant fibrogenesis, linking these events to resolving AT2 cell dysfunction in *Sftpc^I73T^* mice in vivo.

This study has certain limitations that raise important questions and opportunities for future investigation. First, while AMPK signaling is clearly diminished in both our mouse and human models — and its activation improves aspects of AT2 metabolism and promotes a more homeostatic phenotype — we recognize that reduced AMPK activity alone may not be sufficient to drive the dysfunctional AT2 phenotype, given that other pathways are also perturbed in I73T mutant AT2 cells. Additional work including genetic AMPK inhibition in an AT2 cell–specific fashion is required to address this. Also, given our use of metformin, it is important to consider its mechanism of action, which remains incompletely understood in vivo and is often explored in vitro using “suprapharmacological” doses shown to inhibit mitochondrial complex I (72, 73). However, metformin is a poor inhibitor of complex I at physiologically active concentrations (73, 87, 88), and its in vivo biology is incompletely understood. The current understanding of this mechanism is well reviewed by Foretz et al. (89), who highlight the diverse and context-dependent effects of metformin. In our in vivo studies, we present single-cell data showing increased AMPK activity in AT2 cells (Figure 8E), along with improved respiratory capacity (Figure 7H) after metformin treatment. While other targets including partial inhibition of complex I remain possible, further work using improved in vivo reporter systems is needed to delineate metformin’s precise cellular effects. Furthermore, while we cannot determine if metformin’s effect on the mesenchyme is direct or secondary to the epithelial changes, the rescue of AT2 cell respiration and reduction in cytokines produced by AT2 cells during fibrosis in the *Sftpc^I73T^* mouse model suggest that metformin’s beneficial effects are, at least in part, mediated through the amelioration of AT2 cell metabolic dysfunction. We acknowledge that the most direct approach to answering this question would be to employ an AT2 cell–specific genetic mouse. However, given that the *Sftpc* locus drives the AT2 cell dysfunction, we are technically limited by the lack of non-*Sftpc* AT2-specific recombinase models. Additionally, although we demonstrate that glycolytic reprogramming coincides with impaired AT2 cell progenitor function (self-renewal and AT1 differentiation) following *Sftpc^I73T^* expression, future mechanistic studies to establish a causal relationship may elucidate additional therapeutic targets. Finally, while our data strongly suggest a defect in mitochondrial FAO in AT2^I73T^ cells, a complete functional characterization would require additional substrate- and enzyme-specific assays, which were beyond the scope of this study.

In conclusion, our data support a role for epithelial metabolic dysfunction in IPF and contribute to the growing body of evidence that metabolic intervention in this hub stands as a viable approach for the treatment of pulmonary fibrosis.

## Methods

### Sex as a biological variable.

All mouse experiments utilized both male and female mice.

### Sftpc^I73T^ mouse model of pulmonary fibrosis.

Tamoxifen-inducible *Sftpc*^I73T/I73T^ Rosa26ERT2FlpO^+/+^ (a.k.a. I^ER^-*Sftpc^I73T^*) mice expressing an NH_2_-terminal HA-tagged murine *Sftpc^I73T^* mutant allele into the endogenous mouse *Sftpc* locus were previously generated as reported (18); expanded details are in the Supplemental Methods.

### iPSC line generation and maintenance.

The SPC2 iPSC line clones SPC2-ST-C11 and SPC2-ST-B2 were used in this study. Previously detailed (27), and fully described in Supplemental Methods, these clones carry a SFTPC^tdTomato^ reporter. iPSCs used in this study demonstrated a normal karyotype when analyzed by G-banding and/or array Comparative Genomic Hybridization (Cell Line Genetics). Details of iPSC derivation, characterization, and culture are available for free download at https://crem.bu.edu/cores-protocols/#protocols

### iPSC-directed differentiation into iAT2s and maintenance.

To generate iAT2s, we performed PSC-directed differentiation via definitive endoderm into NKX2-1 lung progenitors using methods we previously described (27, 71, 90, 91) and further detailed in the Supplemental Methods. iAT2s were maintained through serial passaging as self-renewing monolayered epithelial spheres (“alveolospheres”) by plating in 3D-Matrigel (Corning) droplets at a density of 400 cells/μL with refeeding every other day with CK+DCI medium, according to our published protocol (90). iAT2 culture quality and purity were monitored at each passage by flow cytometry, with >90% of cells expressing SFTPC^tdTomato^ over time, as we have previously detailed (71, 90).

### Multichannel flow cytometry for identification of lung cell populations.

Previously validated sorting strategies (18, 23, 56, 58, 70) were applied in the following manner. Cells were incubated with antibody mixtures (see Supplemental Table 2). Stained cells were analyzed with an LSR Fortessa (BD Biosciences). AT2 cells were identified as EpCAM^+^CD45^–^ CD31^–^MHCII^hi^CD104^–^. Transitional cells were defined as EpCAM^+^CD45^–^CD31^–^MHCII^hi^CD104^–^CD51^hi^. Fibroblasts were identified as EpCAM^–^CD45^–^CD31^–^CD140a^+^. Quantification of mitochondrial mass was performed using MitoTracker dye, and ΔΨm was quantified using MitoProbe TMRM Assay Kit for Flow Cytometry according to manufacturer instructions. Cell populations were defined, gated, and analyzed with FlowJo software (FlowJo, LLC).

### Isolation of mouse AT2 cells.

Two protocols were used to prepare primary murine AT2 cells based on application. Flow cytometry–based assays and luciferase-based assays used AT2 cells prepared as described above. All other assays requiring larger numbers of AT2 cells applied our previously reported protocol (18, 58, 92). Expanded protocol is in the Supplemental Methods.

### Lung histology.

Whole lungs were fixed by tracheal instillation of 10% neutral buffered formalin (MilliporeSigma) at a constant pressure of 25 cm H_2_O. Sections (6 mm) were stained with hematoxylin and eosin or Masson’s trichrome stains by the Pathology Core Laboratory of Children’s Hospital of Philadelphia. Slides were scanned using an Aperio ScanScope Model: CS2 (Leica) at 10× original magnification; representative areas were captured, exported as TIF files, and processed in Adobe Illustrator.

### BALF collection, processing, and cytokine measurement.

BALF collected from mice using sequential lavages of lungs with 5 × 1 mL aliquots of sterile saline was processed for analysis as previously described (18) and detailed in the Supplemental Methods. Total CCL2 and CCL17 concentration in the enriched cell-free BALF was calculated using mouse CCL2 and CCL17 DuoSet ELISA (R&D Systems) according to the manufacturer’s instructions.

### Measurement of pulmonary function.

At takedown, mice underwent Flexivent (SCIREQ, Inc.) analysis for assessment of lung physiology as previously described (17, 93). Expanded protocol is included in the Supplemental Methods.

### Mitochondrial immunofluorescence, confocal microscopy, and quantitation.

AT2 cells were isolated as described above and seeded on coverslips via an adapted previously published protocol (94). Briefly, coverslips were coated with 20% Matrigel (Corning) and 80% rat tail collagen (Gibco) for 2 hours at 37°C prior to seeding with freshly isolated murine AT2 cells in 10% DMEM overnight. Cells were then stained using Mitotracker red according to manufacturer instructions. After staining, cells were washed and incubated for an additional 4 hours in 10% DMEM to allow for recovery of mitochondrial networks. Cells were then fixed using ice-cold methanol followed by counterstaining using DAPI. Coverslips were imaged under confocal microscopy at 60× original magnification using a Leica Stellaris 5 confocal. Images were quantified in ImageJ using the mitochondrial analyzer suite (95).

### Electron microscopy.

Preparation of lung tissue and acquisition of TEM images of lung sections was performed in the Electron Microscopy Resource Laboratory in the Perelman School of Medicine based on the method of Hayat that includes postfixation in 2.0% osmium tetroxide with 1.5% potassium ferricyanide, as previously published (18). Cut thin sections (60–80 nm) were stained in situ on copper grids with uranyl acetate and lead citrate and examined with a JEOL 1010 electron microscope fitted with a Hamamatsu digital camera and AMT Advantage image capture software.

### RNA isolation and reverse transcriptase quantitative PCR.

RNA was extracted by first lysing cells in QIAzol (QIAGEN) and subsequently using RNeasy Mini Kit (QIAGEN) according to the manufacturer’s protocol and as previously described (27, 56). Detailed protocols are in the Supplemental Methods. Primer sequences for mouse and human genes are listed in Supplemental Table 2.

### Cellular ATP quantification.

AT2 cells were isolated via flow cytometry as described above. ATP was measured using the Firefly Luciferase ATP Assay (MilliporeSigma) according to manufacturer instructions. Briefly, immediately after isolation 100,000 AT2 cells were mixed with d-luciferin, luciferin, and lysis solution in a 96-well plate. Luminescence was then measured using a TECAN Spark multimode plate reader.

### In vitro culture of AT2 cells and measurement of metabolites.

AT2 cells were isolated as described above and seeded in 10% DMEM for up to 48 hours. Validation of mutant SP-C expression was performed via immunoblot analysis. Supernatants were collected after 24 hours in culture followed by measurement of glucose and lactate concentrations using YSI 2500 biochemical analyzer and according to manufacturer instructions.

### Immunoblot analysis.

Total protein was isolated from frozen cell suspensions and processed for immunoblot analysis as previously described (27, 70). Detailed protocols are in the Supplemental Methods. Antibodies are in Supplemental Table 2. Visualization was performed on the Odyssey Imaging System (LICOR Biosciences).

### popRNA-Seq.

Isolated RNA from either primary murine AT2 cells or iAT2s was sent for library preparation by GENEWIZ, LLC. FASTQ files were processed as previously described (70) and detailed in the Supplemental Methods. Heatmaps were generated using Morpheus (https://software.broadinstitute.org/morpheus). Protein-protein interactions were obtained using STRING database. GO analysis was performed using the DAVID based on DEGs (FC > 1.5; *P* < 0.05). Key pathway analyses were performed on gene lists identified from the GSEA molecular signature database (43, 96, 97).

### Analysis of scRNA-Seq.

Distal epithelial cell–specific analysis of our previously published data set (GSE234604) is included in this manuscript. New samples from a naive C57B6 mouse and 2 *Sftpc*^I73T^ mice 28 days after tamoxifen treated with either 150 mg/kg i.p metformin or vehicle were collected as single-cell suspensions prepared by physical and enzymatic dissociation followed by magnetic activated cell sorting by LS columns (Miltenyi Biotec 130-042-401) with CD45^+^ cell removal using CD45 microbeads (Miltenyi Biotec 130-052-301). Library generation and sequencing were performed by the University of Pennsylvania Perelman School of Medicine Next Generation Sequencing Core Facility (RRID:SCR_022382). scRNA-Seq reads were aligned and processed as previously described and detailed in the Supplemental Methods (56, 59).

### Cellular respirometry assays.

Primary mouse AT2 cells (100,000 cells/well) were isolated and plated as described above with measurement of mitochondrial respiration performed using the Seahorse XF Cell Mito Stress Test Kit (Agilent) or the Seahorse XF Palmitate Oxidation Stress Test Kit (Agilent) according to the manufacturer’s instructions. Human iPSC-derived alveolospheres were harvested by incubating with 2 mg/mL dispase (Thermo Fisher Scientific) for 30–60 minutes at 37°C, washed, and resuspended in 150–300 μL of Seahorse XF Base Media Minimal DMEM (Agilent) containing 2.8 mM glucose and 0.1% FBS (pH 7.4). Alveolospheres were seeded in an XF96e Seahorse plate as described (27, 98, 99). This protocol is fully described in the Supplemental Methods.

### Primary mouse organoid culture.

Organoid cultures were performed as previously described (58). Expanded protocol is in the Supplemental Methods.

### Quantification of lactate, pyruvate, and glucose in murine AT2 cells.

A total of 400,000 flow-sorted AT2 cells (EpCAM^+^CD45^–^CD31^–^MHCII^hi^CD104^–^) were collected from *Sftpc^I73T^* and WT mice and evenly divided into 3 aliquots before being flash-frozen. Four biological replicates from each time point were assayed in technical triplicates of about 40,000 cells per replicate using Promega kits Pyruvate-Glo, Glucose-Glo, or Lactate-Glo according to manufacturer instructions.

### Statistics.

All data are presented with dot plots and group mean ± SEM unless otherwise indicated. Statistical analyses were performed with GraphPad Prism. Two-tailed *t* test was used for 2 groups as indicated; multiple comparisons were performed by ordinary 1-way ANOVA with post hoc testing as indicated; survival analyses was performed using Kaplan-Meier with Mantel-Cox correction. In all cases statistical significance was considered at *P* ≤ 0.05.

### Data availability.

The sequencing data generated in this study are deposited in NCBI GEO under accession number GSE296513. Analysis of previously published data was performed on GSE234604 (56).

### Study approval.

Mice housed in pathogen-free facilities were subjected to experimental protocols and studies approved by the Institutional Animal Care and Use Committee at the Perelman School of Medicine at the University of Pennsylvania.

## Author contributions

DNK and MFB developed the concept. LRR, KDA, JK, DNK, and MFB designed the experiments. YT, PC, SI, KC, and CHC performed in vivo animal experiments. LRR, KDA, AM, SB, RAP, AP, KM, PC, SI, KC, and CHC conducted experiments and analyzed data. WRB and AB performed bioinformatic analysis. AIW, AEV, JK, AM, and LRR conceived, validated, and optimized flow cytometry strategies. LRR, KDA, JK, RAP, AP, OSS, DNK, and MFB interpreted data and generated figures. LRR and KDA drafted the original manuscript. LRR, KDA, JK, WRB, RAP, AP, AIW, AEV, ZA, OSS, DNK, and MFB edited the manuscript. All authors reviewed and approved the final version prior to submission.

## Supplementary Material

Supplemental data

Unedited blot and gel images

Supporting data values

## Version 1 07/01/2025

In-Press Preview

## Version 2 08/08/2025

Electronic publication
