# Supplementary material for: Impaired AMPK control of alveolar epithelial cell metabolism promotes pulmonary fibrosis
Source: JCI Insight. 2025 Jul 1;10(15):e182578. doi: 10.1172/jci.insight.182578 (PMC12333953; doi:10.1172/jci.insight.182578)
Supplement: Supplemental data [file jciinsight-10-182578-s055.pdf]

SUPPLEMENTAL METHODS and DATA

Impaired AMPK Control of Alveolar Epithelial Cell Metabolism Promotes Pulmonary Fibrosis

Luis R. Rodríguez[1,2 *], Konstantinos-Dionysios Alysandratos[3,4 *], Jeremy Katzen[1,2], Aditi Murthy[1, 2], Willy Roque Barboza[1,2], Yaniv Tomer[1, 2], Sarah Bui[1,2], Rebeca Acín-Pérez[5], Anton Petcherski[5], Kasey Minakin[3,4], Paige Carson[1,2], Swati Iyer[1,2], Katrina Chavez[1,2], Charlotte H. Cooper[1,2], Apoorva Babu[2], Aaron I. Weiner[2,6,7], Andrew E. Vaughan[2,6,7], Zoltan Arany[8], Orian S. Shirihai[5], Darrell N. Kotton[3,4 ¶], and Michael F. Beers[1, 2 ¶]

[1] Pulmonary, Allergy, and Critical Care Division, Department of Medicine, Perelman School of Medicine at the University of Pennsylvania, Philadelphia, PA 19104, USA

[2] PENN-CHOP Lung Biology Institute, Perelman School of Medicine at the University of Pennsylvania, Philadelphia, PA 19104, USA

[3] Center for Regenerative Medicine, Boston University and Boston Medical Center, Boston, MA 02118, USA

[4]The Pulmonary Center and Department of Medicine, Boston University Chobanian & Avedisian School of Medicine, Boston, MA 02118, USA

[5] Departments of Medicine, Endocrinology and Molecular and Medical Pharmacology, David Geffen School of Medicine at UCLA, Los Angeles, CA 90095, USA

[6] Department of Biomedical Sciences, School of Veterinary Medicine, University of Pennsylvania, Philadelphia, PA 19104, USA

[7] Institute for Regenerative Medicine, University of Pennsylvania, Philadelphia, PA 19104, USA

[8] Cardiovascular Institute, Perelman School of Medicine at University of Pennsylvania 19104 USA

[*] **These authors provided equal contribution to this manuscript**

¶ D.N. Kotton and M.F. Beers are co–senior authors

Running Title: *Epithelial AMPK Signaling in Lung Fibrosis*

Lead Contact Author:

Michael F. Beers[†], M.D.
Pulmonary and Critical Care Division
Perelman School of Medicine at The University of Pennsylvania
Edward J Stemmler Hall Suite 216
3450 Hamilton Walk
Philadelphia, Pennsylvania 19104-6118
e-mail: mfbeers@pennmedicine.upenn.edu

[†] Albert M. Rose Established Investigator of the Pulmonary Fibrosis Foundation

Conflict of Interest: The authors declare that no conflicts of interest exist.

SUPPLEMENTAL METHODS

$Sftpc^{I73T}$ Mouse Model of Pulmonary Fibrosis

Tamoxifen inducible $Sftpc^{I73T/I73T}$ Rosa26ERT2FlpO$^{+/+}$ (a.k.a. IER-$Sftpc^{I73T}$) mice expressing an NH_2-terminal HA-tagged murine $Sftpc^{I73T}$ mutant allele into the endogenous mouse *Sftpc* locus were previously generated as reported(1) and are summarily detailed. Tamoxifen treatment (360 mg/kg in males and 520 mg/kg in females) of adult IER-$Sftpc^{I73T}$ mice was initiated at 12-14 weeks of age by oral gavage (OG) split evenly on day 0 and day 3. All mouse strains and genotypes generated for these studies were congenic with C57/B6/J. Both male and female animals (aged 8-14 weeks) were utilized in tamoxifen induction protocols. All mice were housed under pathogen free conditions in an AALAC approved barrier facility at the Perelman School of Medicine, University of Pennsylvania. All experiments were approved by the Institutional Animal Care and Use Committee at the University of Pennsylvania.

Generation of Single Cell Suspensions from Mouse Lungs

Blood free perfused lungs were digested in Phosphate Buffered Saline (Mg and Ca free) with Collagenase Type I (Gibco Cat# 17100017), DNase (Millipore Sigma Cat# D5025), and Dispase (BD Biosciences). Resulting product was passed through 70 µm nylon mesh to obtain single-cell suspensions and then processed with ACK Lysis Buffer (Thermo Fisher).

iPSC line generation and maintenance

The SPC2 iPSC line clones SPC2-ST-C11 and SPC2-ST-B2 were used in this study. As previously detailed(2), we used TALENs to insert a tdTomato fluorescent reporter at the translation initiation (ATG) site of the endogenous *SFTPC* locus of the parental SPC2 iPSC line, resulting in the generation of either corrected (SPC2-ST-B2 clone; $SFTPC^{tdT/WT}$) or mutant (SPC2-ST-C11 clone; $SFTPC^{I73T/tdT}$) iPSC clones as the tdTomato cassette is followed by a stop/polyA cassette, preventing expression of the subsequent *SFTPC* coding sequence from the targeted allele. iPSCs

used in this study demonstrated a normal karyotype when analyzed by G-banding and/or array Comparative Genomic Hybridization (aCGH, Cell Line Genetics). iPSCs were maintained in feeder-free conditions, on growth factor-reduced Matrigel (Corning) in 6-well tissue culture dishes (Corning), in mTeSR1 media (StemCell Technologies) using gentle cell dissociation reagent for passaging. Further details of iPSC derivation, characterization, and culture are available for free download at https://crem.bu.edu/cores-protocols/#protocols.

iPSC-directed Differentiation into Alveolar Epithelial Type 2 Cells (iAT2s) and Maintenance

To generate iAT2s, we performed PSC-directed differentiation via definitive endoderm into NKX2-1 lung progenitors using methods we have previously described(2–5). On day 15 or 16 of differentiation, live cells were sorted on a high-speed cell sorter (MoFlo Astrios EQ) to isolate NKX2-1+ lung progenitors based on $CD47^{hi}CD26^{-}$ gating(5). Sorted lung progenitors were resuspended in undiluted growth factor-reduced 3D Matrigel (Corning) at a density of 400 cells/µl, and distal/alveolar differentiation of cells was performed in CK+DCI medium, consisting of complete serum-free differentiation medium base supplemented with 3 µM CHIR99021, 10 ng/mL recombinant human KGF (CK), and 50 nM dexamethasone (Sigma), 0.1 mM 8-Bromoadenosine 3′,5′-cyclic monophosphate sodium salt (Sigma), and 0.1 mM IBMX (Sigma) (DCI). The resulting epithelial spheres were passaged without further sorting on approximate day 30 of differentiation followed by a brief period (4-5 days) of CHIR99021 withdrawal to achieve iAT2 maturation, as previously shown(3). After this 2-week period, $SFTPC^{tdTomato+}$ cells were purified by fluorescence activated cell sorting (FACS) to establish pure cultures of iAT2s. iAT2s were then maintained through serial passaging as self-renewing monolayered epithelial spheres (“alveolospheres”) by plating in 3D Matrigel (Corning) droplets at a density of 400 cells/µl with refeeding every other day with CK+DCI medium, according to our published protocol(4). iAT2 culture quality and purity were monitored at each passage by flow cytometry, with > 90% of cells expressing $SFTPC^{tdTomato}$ over time, as we have previously detailed(3, 4).

Bronchoalveolar Lavage Fluid (BALF) Collection, Processing, and Cytokine Measurement

BALF collected from mice using sequential lavages of lungs with five X 1 ml aliquots of sterile saline was processed for analysis as previously described(1). First ml of saline wash is labeled as enriched cell free BALF used to measure total protein and cytokines. Cell pellets obtained by centrifuging BALF samples at 400 g for 6 minutes were re-suspended in 1 ml of PBS, and total cell counts determined using a NucleoCounter (New Brunswick Scientific, Edison, NJ). Differential cell counts were determined manually from BALF cytospins stained with modified Giemsa (Sigma Aldrich, #GS500) to identify macrophages, lymphocytes, eosinophils and neutrophils. Total protein content of cell free BALF was determined using the DC Protein Assay Kit (Cat # 5000111; BioRAD, Inc, Hercules CA) with bovine serum albumin as a standard according to the manufacturer's instructions.

Measurement of Pulmonary Function

At takedown, mice underwent Flexivent (SCIREQ, Inc. Toronto Canada) analysis for assessment of lung physiology as previously described(6, 7). Briefly, invasive measurement of static lung compliance was performed with mice anesthetized with intraperitoneal pentobarbital. The mouse tracheas were cannulated with a 20-gauge metal stub adapter and then placed on a small-animal ventilator at 150 breaths per min and a tidal volume of 10 ml/kg of body weight. Static lung compliance was determined with the manufacturer's software using a 2 second breath pause maneuver.

RNA Isolation and Reverse Transcriptase Quantitative Polymerase Chain Reaction (RT-qPCR)

RNA was extracted by first lysing cells in Qiazol (Qiagen) and subsequently using RNAeasy mini kit (Qiagen) according to the manufacturer's protocol. The concentration and quality of extracted RNA from the lung tissues were measured using NanoDrop® One (Thermo Scientific, Wilmington, DE) and reverse-transcribed into cDNA using Verso cDNA Synthesis Kit (ThermoFisher). RT-qPCR was performed on a QuantStudio 7 Flex Real-Time PCR System with

results normalized to *18S* and *Actb* gene expression. Primer sequences for all mouse and human genes are listed in **Supplemental Table 2**.

Immunoblot Analysis

iAT2s were harvested by incubating with 2 mg/ml dispase (Thermo Fisher Scientific) for 30-60 minutes at 37°C, treated with lysis buffer (RIPA buffer, 1x Roche Complete Protease Inhibitor cocktail, 1X Sigma Phosphatase Inhibitor Cocktail 2, and 1X Sigma Phosphatase Inhibitor Cocktail 3), and incubated on ice for 30 minutes. AT2 cells were either collected immediately after isolation or 48 hours after culture and treated with lysis buffer. Cellular debris was cleared by centrifugation at 15,000 g for 20 minutes and supernatants were harvested. Protein concentration was measured using Bio-Rad DC Protein Assay. 5-30 µg of lysates were resolved on pre-cast 10% or 12% Bis-Tris NuPAGE gels (Invitrogen), transferred to PVDF membranes (Bio-Rad), and blotted with primary antibodies overnight followed by 1 hour species specific secondary antibody incubation. All antibodies are listed in **Supplemental Table 2**. Visualization was performed on the Odyssey Imaging System (LiCOR Biosciences).

Population RNA Sequencing Data Processing

Fastq files from iAT2s and primary murine AT2s were evaluated for quality control with the FastQC program and then aligned against the mouse reference genome (mm10) using the STAR aligner(8). Duplicate reads were flagged with the MarkDuplicates program from Picard tools and excluded from analysis. Per gene read counts for Ensembl (v67) gene annotations were computed using the R package Rsubread. Gene counts, represented as counts per million (CPM), were nominalized using TMM method in the edgeR R package, and genes with 25% of samples with a CPM < 1 were considered low expressed and removed. The data were transformed with the VOOM function from the limma R package to generate a linear model and perform differential gene expression analysis(9). We employed the empirical Bayes procedure as implemented in

limma to adjust the linear fit and to calculate P values given the small sample size of the experiment. We adjusted P values for multiple comparisons using the Benjamini-Hochberg procedure. Heatmaps were generated using the Broad Institute's online tool Morpheus (https://software.broadinstitute.org/morpheus). Protein-protein interactions were obtained using STRING (Search Tool for Retrieval of Interacting Genes/Proteins) database. Gene ontogeny analysis was performed using the Database for Annotation, Visualization, and Integrated Discovery (https://david.ncifcrf.gov/home.jsp) based on differentially enriched genes (FC >1.5 P-value <0.05). Key pathway analyses were performed on gene lists identified from the GSEA molecular signature database(10–12).

Single Cell RNA Sequencing Data Processing

Single cell RNA-Seq reads were aligned to mouse genome (mm10/GRCm38) using STARSolo (version 2.7.5b). After initial quality control and processing, we analyzed the scRNA-seq data using the Scanpy pipeline(13). Genes expressed in fewer than 3 cells were removed, and cells with fewer than 200 genes and a mitochondrial fraction of more than 20% were excluded. Counts were log-normalized using scanpy.pp.normalize_per_cell (counts_per_cell_after=$1x10^4$), followed by scanpy.pp.log1p. To integrate data from multiple samples, we used Scvi-tool(14). We applied scvi.model.SCVI.setup_anndata() to establish the model parameters for integration, including: layer, categorical_covariate_keys, and continuous_covariate_keys. We then performed a principal component analysis (PCA) and generated a K-nearest neighbor (KNN) graph using scanpy.pp.neighbors with n_neighbors=15. The resulting KNN graph was used to perform Uniform Manifold Approximation and Projection (UMAP) dimension reduction to visualize the cells in two dimensions using scanpy.tl.umap(). Clustering was performed using the Leiden algorithm with scanpy.tl.leiden(15). We identified cell populations using known canonical marker genes or by assessing cluster-defining genes based on differential expressions. Epithelial cells were clustered into proximal and distal clusters as reported in Supplemental Figures 3 and 9. Additionally, we

performed linear trajectory inference on the UMAP reduction using scFates(16) with the AT2 cluster as the starting point and without assigned endpoints. Finally, we performed gene ontology analysis for enriched biological processes using GSEApy(17) based on differentially enriched genes between the groups.

Isolation of Mouse AT2 Cells

Assays requiring larger numbers of AT2 cells applied our previously reported protocol(1, 18, 19). Briefly, perfused mouse lungs were digested as described for flow cytometry to obtain a single cell suspension. Negative selection of mesenchymal cells via differential adherence on plastic culture dishes was followed by CD45 depletion using simultaneous incubation with Dynabeads untouched mouse T cell kit (Thermo Fisher #11413D) and Dynabeads mouse DC enrichment kit (#11429D) (Thermo Fisher Scientific). Subaliquots were analyzed for purity (greater than 90%) by flow cytometry using EpCAM+, proSP-C+ staining.

Primary Mouse Organoid Culture

Organoid culture assays were performed as previously described(18). AT2 cells and fibroblasts were flow sorted as described above. In each technical replicate, 5000 AT2 cells were combined with 50,000 *Pdgfra*$^{+}$ lung fibroblasts in 50% Matrigel (Corning) and 50% SAGM (Lonza) in a Falcon Cell Culture Insert. Cell/matrigel suspension solidified and SAGM medium was then added into the bottom of the well. SAGM was prepared according the BulletKit (Lonza) manufacturer instructions with some modification (Hydrocortisone, BSA, Triiodothyronine, and Epinephrine were not included). Medium was changed every other day. 10 µM rock inhibitor (Y-27632 dihydrochloride, Millpore Sigma, catalog # Y0503) was added to the medium for the first two days of culture. Organoids were imaged on an EVOS FL Microscope and were quantified via ImageJ using the “analyze particles” macro.

Cellular Respirometry assays

Human iPSC-derived alveolospheres were harvested by incubating with 2 mg/ml dispase (Thermo Fisher Scientific) for 30-60 minutes at 37°C, washed, and resuspended in 150-300 µl of Seahorse XF Base Media Minimal DMEM (Agilent Technologies) containing 2.8 mM glucose and 0.1 % FBS (pH 7.4). Alveolospheres were seeded in an XF96e Seahorse plate as described(2, 20, 21). Briefly, growth factor reduced Matrigel (1.5 µl/well; Corning) was first pipetted in the center measurement zone of each well. The alveolosphere suspension (5-10 µl) was deposited in the Matrigel-coated measurement zone using a pipette. The plate was then incubated in a non-CO_2 incubator for 3.5 minutes to let the Matrigel solidify. Next, 150 µl of pre-warmed Seahorse media was slowly added to each well to avoid dislodging the organoids from the Matrigel. The plate containing the organoids was centrifuged at 500 g for 5 minutes with no brake and subsequently incubated in a non-CO_2 incubator for 30-45 minutes prior to running the assay. Oxygen consumption was determined as described(20, 21). Port injections were as follows: port A, oligomycin (final concentration 4.5 µM/l); port B, FCCP diluted in a mixture of 80% sodium pyruvate and 20% of 1:1 l-leucine/l-glutamine (final concentration 1 µM/l FCCP in 11.4 mM/l sodium pyruvate and 2.9 mM/l each of leucine/glutamine); and port C, antimycin A (final concentration 2.5 µM/l). Once the Seahorse assay was completed, the plate was kept for mitochondrial content determination. Protein concentration was determined by BCA assay (Thermo Fisher Scientific).

Primary mouse AT2 cells (100,000 cells/well) were isolated and plated as described above with measurement of mitochondrial respiration performed using the Seahorse XF Cell Mito Stress Test Kit (Agilent) or the Seahorse XF Palmitate Oxidation Stress Test Kit (Agilent) according to the manufacturer’s instructions. Prior to the initiation of the assay cells were maintained in either DMEM or, for small molecule challenge, under the various small molecules described in the results and figure legends. As with alveolospheres, stress test conditions included 2.5 µg/ml

oligomycin, 2 μM FCCP, and 0.5 μM rotenone/antimycin A. Protein concentration was determined using DC protein assay (Bio-Rad).

Quantification of NF-κB signaling

To track the kinetics of the NF-κB pathway in iAEC2s, we utilized a lentiviral vector (lenti-NF-κB-luc-GFP) we have previously published[27] that allows independent simultaneous tracking of transduced (GFP^+) cells by flow cytometry and assessment of NF-κB activation levels by luciferase expression. This dual transgenesis vector contains four tandem copies of the canonical NF-κB p50/p65 heterodimer consensus binding sequence located upstream of the minimal TA promoter (TAp), the TATA box of the herpes simplex virus thymidine kinase (HSV-TK) promoter. Located downstream from TAp is the firefly luciferase reporter coding sequence, followed by a constitutively active mammalian ubiquitin C (UBC) promoter driving GFP expression. Mutant ($SFTPC^{I73T/tdT}$) or corrected ($SFTPC^{tdT/WT}$) iAEC2s were transduced with the lentiviral vector at an MOI of 20 in CK+DCI media containing polybrene (5 mg/ml). iAEC2s were then replated in growth factor reduced 3D Matrigel (Corning) at a concentration of approximately 400 cells/μl in CK+DCI media. Thirteen days later, mutant and corrected iAEC2s (n=3 independent droplets) were dissociated into single cells and sorted for transduced (GFP^+) $SFTPC^{tdTomato+}$ cells which were subsequently resuspended in 75 μl FACS buffer and plated in a 96-well flat bottom black polystyrene microplate (Corning). Cells were then processed according to the Dual-Glo Luciferase Assay System Protocol (Promega). Firefly luminescence was measured using the Tecan Infinite M200 Pro Micro Plate Reader (Tecan, Männedorf, Switzerland).

Supplemental Table 1: Transitional Cell Gene Modules

PATS[63]	Cell Cycle Arrest[62]	DATPs[64]	KRT5-/KRT17+[65]	Aberrant Basaloid[66]	Krt8+ ADI[60]	Subpopulation 1[68]	Cluster 7[69]	ABI1[67]	ABI2[67]
S100a6	Cdk4	Cldn4	Krt17	Cdh1	Sprr1a	Cldn4	Fermt1	Prss2	Vim
Sfn	Trp53	Krt8	Prss2	Cdh2	Cldn4	Sprr1a	Fn1	Slc34A2	Lgals1
Tmsb10	Cdk2b	Cdkn1a	Krt7	Spink1	Cdkn1a	Tnip3	Ltpb1	Pkib	Mt2A
Cldn4	Ccnd1	Ndrg1	Gdf15	Mmp7	Plaur	Plaur	Ctse	Ctse	Ftl
Clu	Aqp5	Sprr1a	Mmp7	Ptgs2	Tnip3	Prss23	Itga2	Slc22A31	Rtf1
AW112010	Emp2	Tnip3	Sox4	Cdkn2a	Tnfrsf12a	AW112010	Fblim1	Spink1	Srsf2
Krt19	Nfkbia	Mif	Tacstd2	Cdkn2b	Edn1	Cyr61	Ankrd1	Vstm2L	Phlda3
Krt18	Fn1	Pold4	Sfn	Hmga2	Tmp2	Fn1	Palld	Myo1G	Pnisr
Anxa1	Gadd45b	Mboat1	Ociad2	Epcam	S100a6	Tpm2	Pdlim7	Slco2A1	Ccdc12
Krt8	Serpine1	Hif1a	Mdk	Vim	S100a10	Bok	Pmepa1	Kcnq3	Ppp2R2A
Tpm2	Ctgf	Pdk4	S100a2	Fn1	Anxa1	Camp	Plekha2	Serpina1	Mrps26
Krt7	Pdgfb	Cxx1b	Itgb6	Col1a1	Prkcdbp	Clu	Ctgf	Prss1	Amd1
Serpinb9	Tgif1	Lrrc26	Tm4sf1	Cdh2	S100a14	Itgb6	Grasp	Vsig1	Vps4B
Anxa2	Itgb6	Cdkn2a	Krt8	Tnc	Lgals3	Ngp	Lgals1	Tnnc1	Srsf11
Ifitm3	Nfib	Mdm2	Krt18	Vcan	Cyr6l	F3	Itpr3	Rab27B	Imp3
Epcam	Itgav	Ccnd1	Tpm1	Pcp4	Atf3	Prkcdbp	Tubb2b	Rhof	Hnrnpa0
Lgals3	Tgfb2	Gdf15	Ceacam6	Cux2	Krt18	Sfn	Ptgs2	Napsa	Arglu1
Tnip3	Tgfbi	Trp53	Krt19	Prss2	Cryab	Anxa1	Slc4a4	Hopx	Prrc2C
Sox4		Bax	Pcsk1n	Cpa6	Sfn	Ly6a	Elk3	Wfdc3	Srsf3
Anxa5		Infgr1	Ptgs2	Ctse	Rap2b	Fxyd3	Flnb	Lmo7	Jund
Cyr61		Ly6a	Ctse	Mdk	Myl12a	Tubb2b	Mcam	Nkx2-1	Srsf5
Cavin3		Irf7	Hopx	Gdf15	Rps27l	Areg	Sox4	Cda	Golgb1
Tuba1a		Cxcl16	Col1a1	Slco2a1	Tpm1	Krt19	Bcr	C6Orf223	Srsf7
S100a11		Timp1	C8orf4	Ephb2	Anxa3	Tnfrsf12a	Basp1	Sftpb	Tes
Serpinb1a			Gprc5a	Itgb8	S100a11	Gsto1		Sfta2	Ddx17
Crip1			Pon2	Itgav	Krt8	S100a14		Dpysl2	Ifi16
Lgals1			Lamb3	Itgb6	Anxa5	Krt7		Abcc3	Sypl1
Ccl20			Cldn4	Tgfbi	Anxa2	Tuba1a		Mgll	Sinhcaf
Actn1			Tram1	Kcnn4	Cd81	Malt1		Msln	Net1
Mfge8			Epcam	Kcnq5	Cstb	Ceacam1		Arhgap29	Mgst2
Ubd			Fhl2	Kcns3	Krt7	Emp3		Tmem139	Ccdc186
Tubb5			Itga2	Cdkn1a	Ubb	Eno1		Icam1	Ppig
Ywhah			Ccnd2	Ccnd1	Epcam	Krt18		Ftl	Nfkbiz

S100a10			Tagln	Ccnd2	Hspb1	Krt8		Lpin2	Lima1
Hspb1			Phlda2	Mdm2	Ccng1	Hbegf		Mrpl14	Ifngr1
2200002<br>D01Rik			Cst6	Hmga2	Msn	S100a6		Tmc5	Hnrnpf
Nfkbia			Ccnd1	Ociad2	Rpl19	Mfge8		Chi3L1	Rab14
Cyba			Tnc	Ptchd4	Rps5	Cks2		Adgrf5	Tsc22D<br>1
F3			Sdc1		Rplp0	S100a8		Cited2	Arf6
Cd24a			Cdkn2a		Sqstm1	Cxcl2		Fbp1	Wasf2
Cd81			C19orf33		Igfbp7	St3gal4		Faxdc2	Clint1
Lurap1l			Lamc2		Calm2	Scgb3a2		S100A9	Zmynd<br>8
Myl12a			Pcp4		Eef1a1	Atf3		Dpp4	Klf3
S100a14			Lbh		Rps19	Ddx39		Sfta3	Vapa
Tpm1			Pmepa1		Pkm	Thbs1		Arl14	Ptp4A1
Igfbp7			Zfp36l1		B2m	Ier5		Hpcal1	H1Fx
Marcksl1			Cdh1		Arpc2	Ly6d		Pros1	Socs3
Cfl1			Icam1		Esd	Slc26a4		Ifi27	Irf2Bp2
			Tnfrsf12a		Hsp90a<br>b1			Agr3	Ugcg
			Cd24		Cystm1			Gls	Baz1A
					Clu				
					Psmd8				

Supplemental Table 2: Antibodies and Primer Sequences	
Antibodies	
AMPK alpha (D5A2) Rabbit mAb	Cell Signaling
Rabbit Anti-AMPK-alpha, phospho (Thr172) Monoclonal Antibody, Unconjugated, Clone 40H9	Cell Signaling
Rabbit Anti-Beta Actin	Proteintech
Rabbit Anti-GAPDH	Proteintech
Phospho-PGC1 alpha	Novus
Rabbit Anti-PGC1 alpha Polyclonal, Unconjugated	Novus
Rabbit Anti-HK1	Proteintech
Rabbit LDHA-Specific Antibody, Polyclonal	Proteintech

Rabbit LDHB-Specific antibody, Polyclonal	Proteintech
Rabbit Anti-MDH2, Polyclonal	Proteintech
Rabbit Anti-SQSTM1/p62, Polyclonal	Cell Signaling
TOM20	Proteintech
DRP1 (D8H5) Rabbit mAb	Cell Signaling
Phospho-DRP1 (Ser616) (D9A1) Rabbit mAb	Cell Signaling
MFF (E5W4M) XP® Rabbit mAB	Cell Signaling
Phospho-MFF (Ser146) Antibody	Cell Signaling
Mouse Anti-PINK1 Monoclonal Antibody	Abcam
Rabbit Anti-PARKIN Polyclonal	Proteintech
Rabbit Anti-Total ACC Monoclonal Antibody	Cell Signaling
Phospho-Acetyl-CoA Carboxylase (Ser79) (D7D11) Rabbit Antibody	Cell Signaling
Rabbit polyclonal to LC3B	Cell Signaling
Pro SP-C N terminal	Beers et al. 1994
Pro SP-B PT3	Beers et al. 1992
Rat Anti-KRT8	DSHB Iowa
Monoclonal Anti-Actin, α-Smooth Muscle	Sigma
Goat Anti-Mouse IgG (H+L), HRP conjugated	Biorad
Goat Anti-Rabbit IgG (H+L), HRP conjugated	Biorad
CD45 (30F-11) BB515	Biolegend
Epcam (G8.8) BV711	Biolegend
Epcam (G8.8) BV785	BD Biosciences
CD31 (MEC13.3) APC/Cy7	Biolegend
CD104 (346-11A) PE/Cy7	Biolegend
CD51 (RMV-7) PE	Biolegend
I-A/I-E,MHCII (M5/114.15.2) APC	Biolegend
CD140a/PDGFRa (APA5) APC	Biolegend

Primer Sequences	
Abca3 (Mm01299912_m1)	Thermofisher
Sftpb (Mm00455678_m1)	Thermofisher
Sftpc (Mm00488144_m1)	Thermofisher
Mouse_Cldn4_Forward (GTCCTGGGAATCTCCTTGGC)	Sigma
Mouse_Cldn4_Reverse (TCTGTGCCGTGACGATGTTG)	Sigma
Mouse_Lgals3_Forward (AGACAGCTTTTCGCTTAACGA)	Sigma
Mouse_Lgals3_Reverse (GGGTAGGCACTAGGAGGAGC)	Sigma
Mouse_Krt8_Forward (TCCATCAGGGTGACTCAGAAA)	Sigma
Mouse_Krt8_Reverse (CCAGCTTCAAGGGGCTCAA)	Sigma
Human_ND6-Forward(CCCCATGCCTCAGGATACTC)	Sigma
Human_ND6-Reverse(TTGTTAGCGGTGTGGTCGG)	Sigma
Human_ND2-Forward(AGCACCACGACCCTACTACT)	Sigma
Human_ND2-Reverse(TGGTGGGGATGATGAGGCTA)	Sigma
Human_ATP6-Forward(ACCACAAGGCACACCTACAC)	Sigma
Human_ATP6-Reverse(TATTGCTAGGGTGGCGCTTC)	Sigma
Human_SIRT1-Forward(TTGGCACAGATCCTCGAACAA)	Sigma
Human_SIRT1-Reverse(ATGAAACAGACACCCCAGCTC)	Sigma
Human_SIRT3-Forward(GAGGCGTCAAAGAGTGTGGG)	Sigma
Human_SIRT3-Reverse(CCCCGGCGATCTGAAGTC)	Sigma
Human_ND1-Forward(TCTGGCAGCTGCAGGAAACT)	Sigma
Human_ND1-Reverse(CGGACATCACTCCGGACATT)	Sigma
Human_PPARGC1a-Forward(TCTGCGGGATGATGGCTATG)	Sigma
Human_PPARGC1a-Reverse(TGTTACCTGCGCAAGCTTCT)	Sigma
Mouse_Pdpn_Forward (TTGTGACCCCAGGTACAGGA)	Sigma
Mouse_Pdpn_Reverse (TGGCAAGCCATCTCTATTGGG)	Sigma
Mouse_Ager_Forward (CTTGCTCTATGGGGAGCTGTA)	Sigma
Mouse_Ager_ Reverse (GGAGGATTTGAGCCACGCT)	Sigma
Mouse_Aqp5_Forward (TCTTGTGGGGATCTACTTCACC)	Sigma
Mouse_Aqp5_ Reverse (TGAGAGGGGCTGAACCGAT)	Sigma

Mouse_Scgb3a2_Forward (AGAAGTGTGTGGACGAGCTG)	Sigma
Mouse_Scgb3a2_ Reverse (CAGGTGTGAAAGAGCCTCAAATG)	Sigma
Mouse_Scgb1a1_Forward (AACATCATGAAGCTCACGGAGA)	Sigma
Mouse_Scgb1a1_ Reverse (AGGGCAGTGACAAGGCTTTA)	Sigma
Mouse_Foxj1_Forward (GGGAGGTGGGAGGAACTTCT)	Sigma
Mouse_Foxj1_ Reverse (CGAATGTGAGGCCTGGCT)	Sigma

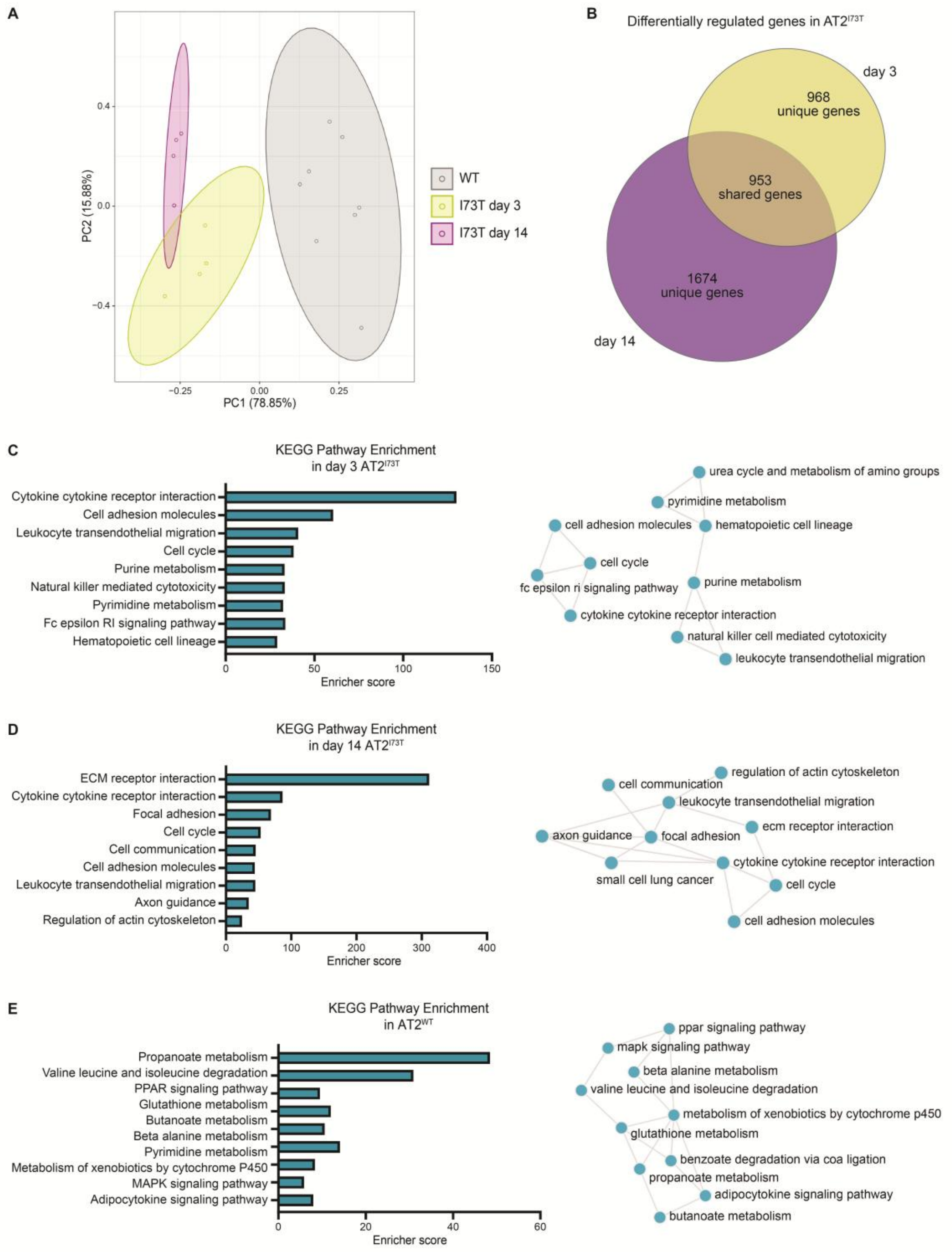
A
PC2 (15.88%)
PC1 (78.85%)
0.4
0.0
−0.4
−0.25
0.00
0.25
WT
I73T day 3
I73T day 14
B
Differentially regulated genes in AT2I73T
day 3
968
unique genes
953
shared genes
1674
unique genes
day 14
C
KEGG Pathway Enrichment
in day 3 AT2I73T
Cytokine cytokine receptor interaction
Cell adhesion molecules
Leukocyte transendothelial migration
Cell cycle
Purine metabolism
Natural killer mediated cytotoxicity
Pyrimidine metabolism
Fc epsilon RI signaling pathway
Hematopoietic cell lineage
0
50
100
150
Enricher score
urea cycle and metabolism of amino groups
pyrimidine metabolism
cell adhesion molecules
hematopoietic cell lineage
cell cycle
fc epsilon ri signaling pathway
purine metabolism
cytokine cytokine receptor interaction
natural killer cell mediated cytotoxicity
leukocyte transendothelial migration
D
KEGG Pathway Enrichment
in day 14 AT2I73T
ECM receptor interaction
Cytokine cytokine receptor interaction
Focal adhesion
Cell cycle
Cell communication
Cell adhesion molecules
Leukocyte transendothelial migration
Axon guidance
Regulation of actin cytoskeleton
0
100
200
300
400
Enricher score
regulation of actin cytoskeleton
cell communication
leukocyte transendothelial migration
ecm receptor interaction
axon guidance
focal adhesion
small cell lung cancer
cytokine cytokine receptor interaction
cell cycle
cell adhesion molecules
E
KEGG Pathway Enrichment
in AT2WT
Propanoate metabolism
Valine leucine and isoleucine degradation
PPAR signaling pathway
Glutathione metabolism
Butanoate metabolism
Beta alanine metabolism
Pyrimidine metabolism
Metabolism of xenobiotics by cytochrome P450
MAPK signaling pathway
Adipocytokine signaling pathway
0
20
40
60
Enricher score
ppar signaling pathway
mapk signaling pathway
beta alanine metabolism
valine leucine and isoleucine degradation
metabolism of xenobiotics by cytochrome p450
glutathione metabolism
benzoate degradation via coa ligation
propanoate metabolism
adipocytokine signaling pathway
butanoate metabolism

Figure S1. Population RNA sequencing (popRNA-seq) of AT2^{I73T} cells isolated 3 and 14 days after *in vivo* tamoxifen administration and AT2^{WT} cells. A) Principal component analysis of popRNA-seq data derived from AT2^{WT} and AT2^{I73T} cells, collected 3- and 14-days post tamoxifen induction. B) Venn diagram highlighting differentially expressed genes when comparing AT2^{I73T} at 3- and 14-days post induction to AT2^{WT}. C) KEGG pathway enrichment analysis of differentially expressed genes in day 3 AT2^{I73T} compared to AT2^{WT} cells. D) KEGG pathway enrichment analysis of differentially expressed genes in day 14 AT2^{I73T} compared to the AT2^{WT} cells. E) KEGG pathway enrichment analysis of differentially expressed genes in AT2^{WT} compared to AT2^{I73T} at 3- and 14-days post tamoxifen induction. C-E) Analysis was performed using Enrichr and presented as both bar graphs and network trees.

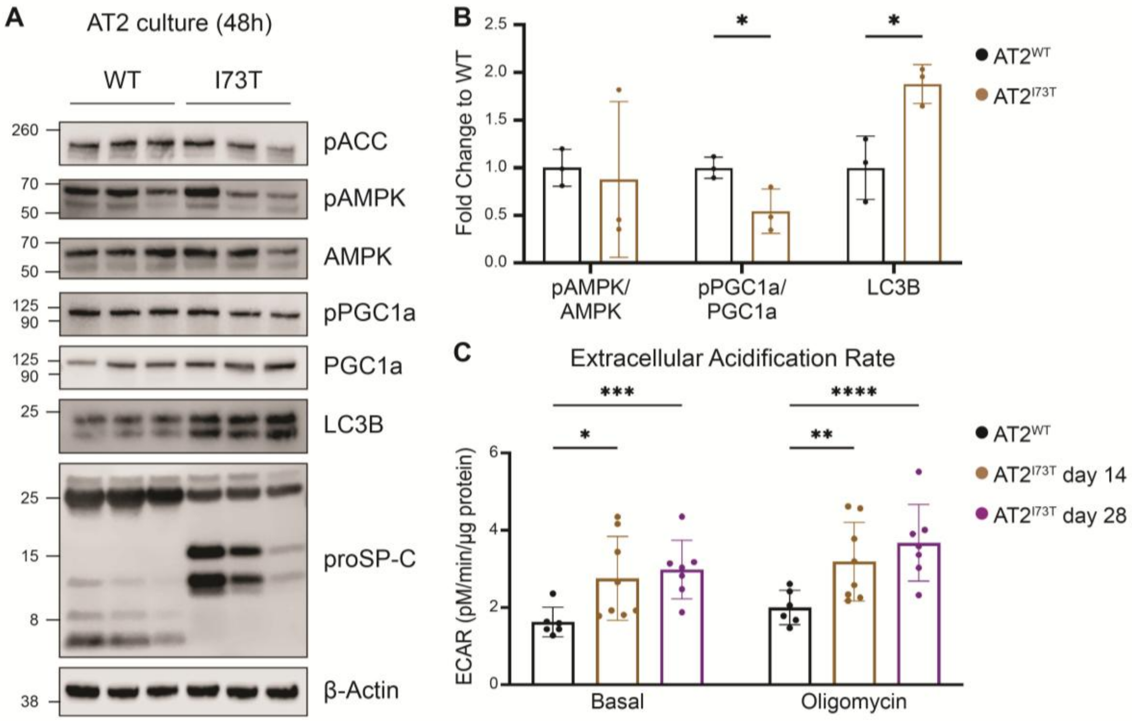

Figure S2. Forty-Eight Hour Culture of Primary Murine AT2 cells. A- B) Western blot analysis and densitometric quantification (mean ± SEM; n = 3 biological replicates) of whole cell lysates from 48-hour AT2 cell cultures, isolated 14 days post *in vivo* tamoxifen induction and cultured in 5% DMEM for 48 hours. Quantification confirms autophagy dysfunction, as evidenced by LC3B accumulation and continued accumulation of processing intermediates in $AT2^{I73T}$ cells. Additionally, sustained loss of mitochondrial biogenesis is observed through the 48-hour culture. C) Measurement of extracellular acidification rate (ECAR) shows an increase in $AT2^{I73T}$ cells isolated from mice 14 and 28 days post *in vivo* tamoxifen induction and cultured overnight (mean ± SEM; n = 6-7 mice per condition). *p < 0.05 ** p<0.005 *** p<0.0005 **** p<0.00005, by ordinary one-way ANOVA.

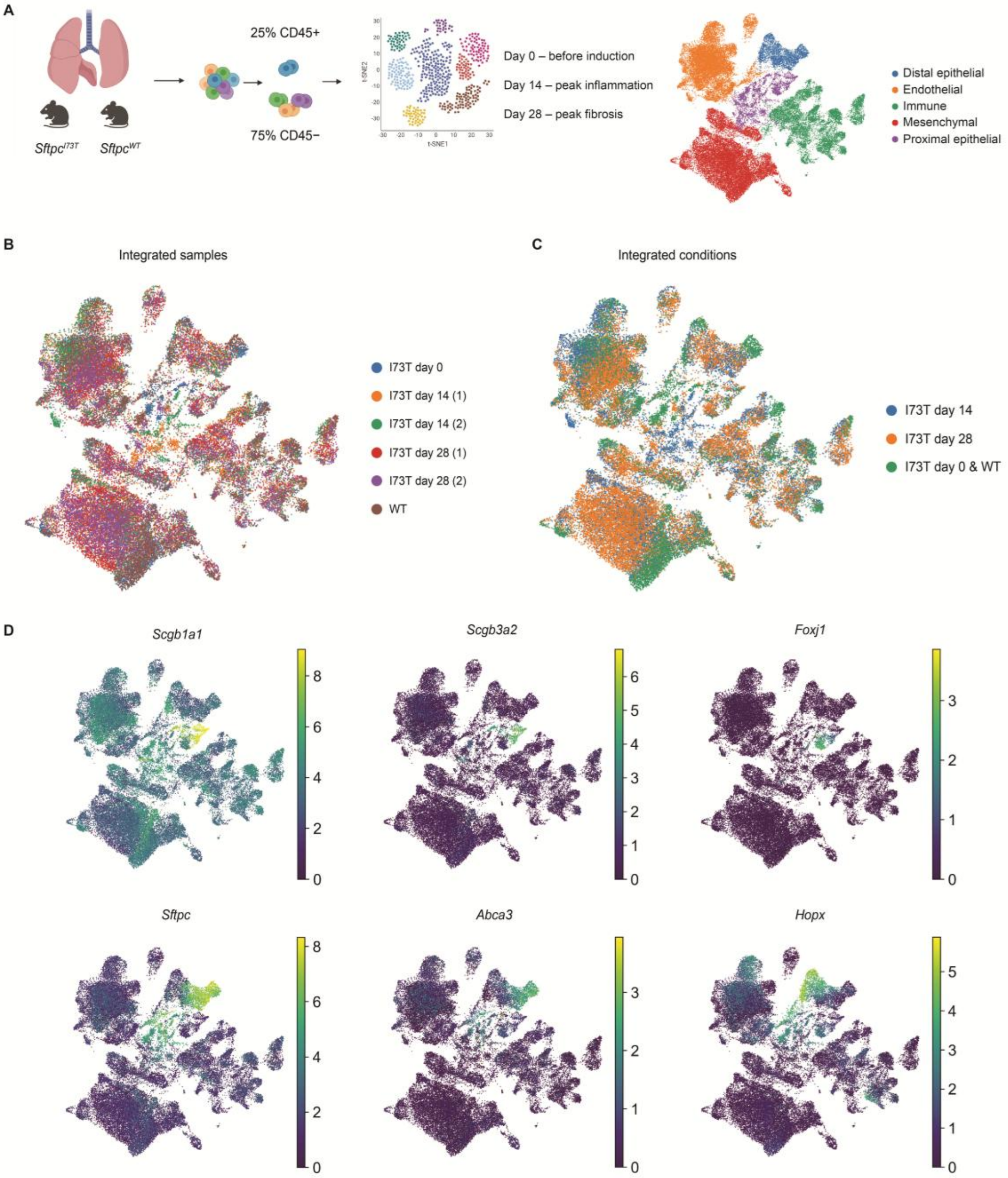
A
25% CD45+
75% CD45−
Sftpc I73T
Sftpc WT
Day 0 – before induction
Day 14 – peak inflammation
Day 28 – peak fibrosis
Distal epithelial
Endothelial
Immune
Mesenchymal
Proximal epithelial
B
Integrated samples
I73T day 0
I73T day 14 (1)
I73T day 14 (2)
I73T day 28 (1)
I73T day 28 (2)
WT
C
Integrated conditions
I73T day 14
I73T day 28
I73T day 0 & WT
D
Scgb1a1
Scgb3a2
Foxj1
Sftpc
Abca3
Hopx

Figure S3. Reanalysis of single cell RNA sequencing (scRNA-seq) profiles (GSE234604)(22)**) of *Sftpc*^{I73T} mouse lungs (14 and 28 days post tamoxifen induction) and WT mouse lungs.** A) Schematic representation of the scRNA-seq strategy used to generate the GSE234604(22) dataset, which involved depletion and "add-back" spiking of CD45+ cells to achieve a final ratio of 25% immune cells and 75% non-immune cells. UMAP projection of the integrated dataset highlighting five major compartments: distal epithelial, proximal epithelial, endothelial, immune, and mesenchymal. B) UMAP projection of the integrated dataset color-coded by individual samples. C) UMAP projection of the integrated dataset color-coded by the model timepoint. The day 0 *Sftpc*^{I73T} sample and *Sftpc*^{WT} sample cluster together and are marked with the same color. D) UMAP projection of the integrated dataset highlighting the expression of genes used to identify distal and proximal epithelial clusters.

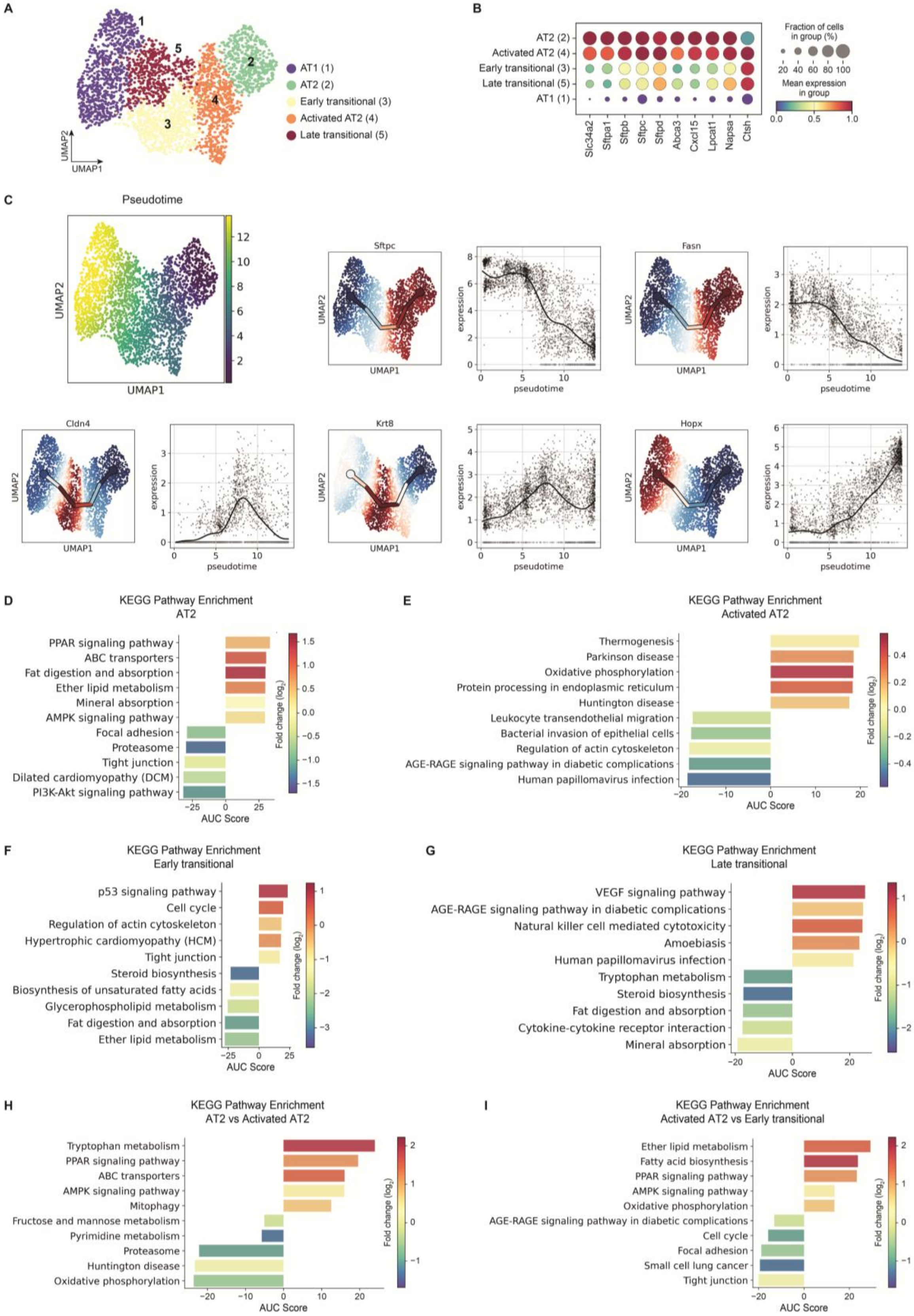
A
AT1 (1)
AT2 (2)
Early transitional (3)
Activated AT2 (4)
Late transitional (5)
UMAP1
UMAP2
B
AT2 (2)
Activated AT2 (4)
Early transitional (3)
Late transitional (5)
AT1 (1)
Slc34a2
Sftpa1
Sftpb
Sftpc
Sftpd
Abca3
Cxcl15
Lpcat1
Napsa
Ctsh
Fraction of cells in group (%)
20 40 60 80 100
Mean expression in group
0.0 0.5 1.0
C
Pseudotime
Sftpc
Fasn
Cldn4
Krt8
Hopx
expression
pseudotime
D
KEGG Pathway Enrichment
AT2
PPAR signaling pathway
ABC transporters
Fat digestion and absorption
Ether lipid metabolism
Mineral absorption
AMPK signaling pathway
Focal adhesion
Proteasome
Tight junction
Dilated cardiomyopathy (DCM)
PI3K-Akt signaling pathway
AUC Score
Fold change (log2)
E
KEGG Pathway Enrichment
Activated AT2
Thermogenesis
Parkinson disease
Oxidative phosphorylation
Protein processing in endoplasmic reticulum
Huntington disease
Leukocyte transendothelial migration
Bacterial invasion of epithelial cells
Regulation of actin cytoskeleton
AGE-RAGE signaling pathway in diabetic complications
Human papillomavirus infection
AUC Score
F
KEGG Pathway Enrichment
Early transitional
p53 signaling pathway
Cell cycle
Regulation of actin cytoskeleton
Hypertrophic cardiomyopathy (HCM)
Tight junction
Steroid biosynthesis
Biosynthesis of unsaturated fatty acids
Glycerophospholipid metabolism
Fat digestion and absorption
Ether lipid metabolism
AUC Score
G
KEGG Pathway Enrichment
Late transitional
VEGF signaling pathway
AGE-RAGE signaling pathway in diabetic complications
Natural killer cell mediated cytotoxicity
Amoebiasis
Human papillomavirus infection
Tryptophan metabolism
Steroid biosynthesis
Fat digestion and absorption
Cytokine-cytokine receptor interaction
Mineral absorption
AUC Score
H
KEGG Pathway Enrichment
AT2 vs Activated AT2
Tryptophan metabolism
PPAR signaling pathway
ABC transporters
AMPK signaling pathway
Mitophagy
Fructose and mannose metabolism
Pyrimidine metabolism
Proteasome
Huntington disease
Oxidative phosphorylation
AUC Score
I
KEGG Pathway Enrichment
Activated AT2 vs Early transitional
Ether lipid metabolism
Fatty acid biosynthesis
PPAR signaling pathway
AMPK signaling pathway
Oxidative phosphorylation
AGE-RAGE signaling pathway in diabetic complications
Cell cycle
Focal adhesion
Small cell lung cancer
Tight junction
AUC Score

Figure S4. Pathway enrichment analysis of *Sftpc*^{I73T} distal alveolar scRNA-seq. A) UMAP projection of the integrated distal alveolar dataset, identifying five clusters. B) Dot plot showing the expression of AT2-associated genes in the five clusters identified in the distal alveolar UMAP. C) Pseudotime analysis of the distal alveolar UMAP with individual gene pseudotime plots representing select key markers of the AT2-to-AT1 transition. D) KEGG pathway enrichment analysis identifying the top 5 pathways that are significantly enriched and decreased in the AT2 cell cluster (cluster 2) compared to the other 4 clusters. E) KEGG pathway enrichment analysis identifying the top 5 pathways that are significantly enriched and decreased in the activated AT2 cluster (cluster 4) compared to the other 4 clusters. F) KEGG pathway enrichment analysis identifying the top 5 pathways that are significantly enriched and decreased in the early transitional cluster (cluster 3) compared to the other 4 clusters. G) KEGG pathway enrichment analysis identifying the top 5 pathways that are significantly enriched and decreased in the late transitional cluster (cluster 5) compared to the other 4 clusters. (H) KEGG pathway enrichment analysis identifying the top 5 pathways that are significantly enriched and decreased in the AT2 cell cluster (cluster 2) compared to the activated AT2 cluster (cluster 4). (I) KEGG pathway enrichment analysis identifying the top 5 pathways that are significantly enriched and decreased in the activated AT2 cluster (cluster 4) compared to the early transitional cluster (cluster 3).

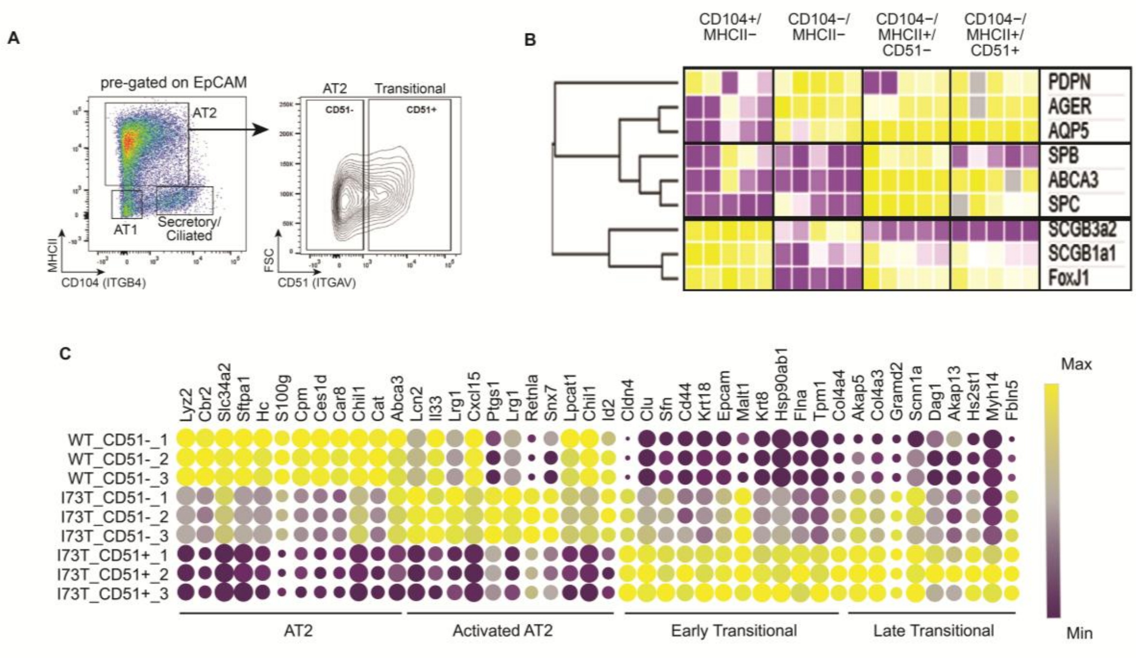

Figure S5. Flow cytometry gating strategy for the isolation of transitional AT2 cells. A) Flow cytometry gating strategy used to isolate transitional AT2 cells. Pre-gating for live cells that are CD45^{neg}, CD31^{neg}, and Epcam^{+} is not shown. B) Heatmap of qPCR-derived gene expression fold changes in distal alveolar identity genes across the various populations isolated using the transitional AT2 gating strategy. Gene expression is normalized to the mean expression of each gene across the four populations. Hierarchical clustering using Euclidean distance was performed on heatmap rows. C) Heatmap analysis of RNA-seq data from WT and I73T MHCII+ cells isolated using the transitional AT2 gating strategy. Select cluster genes derived from single cell analysis in Figure 4 were used to validate enrichment of early and late transitional cells in the CD51+ population.

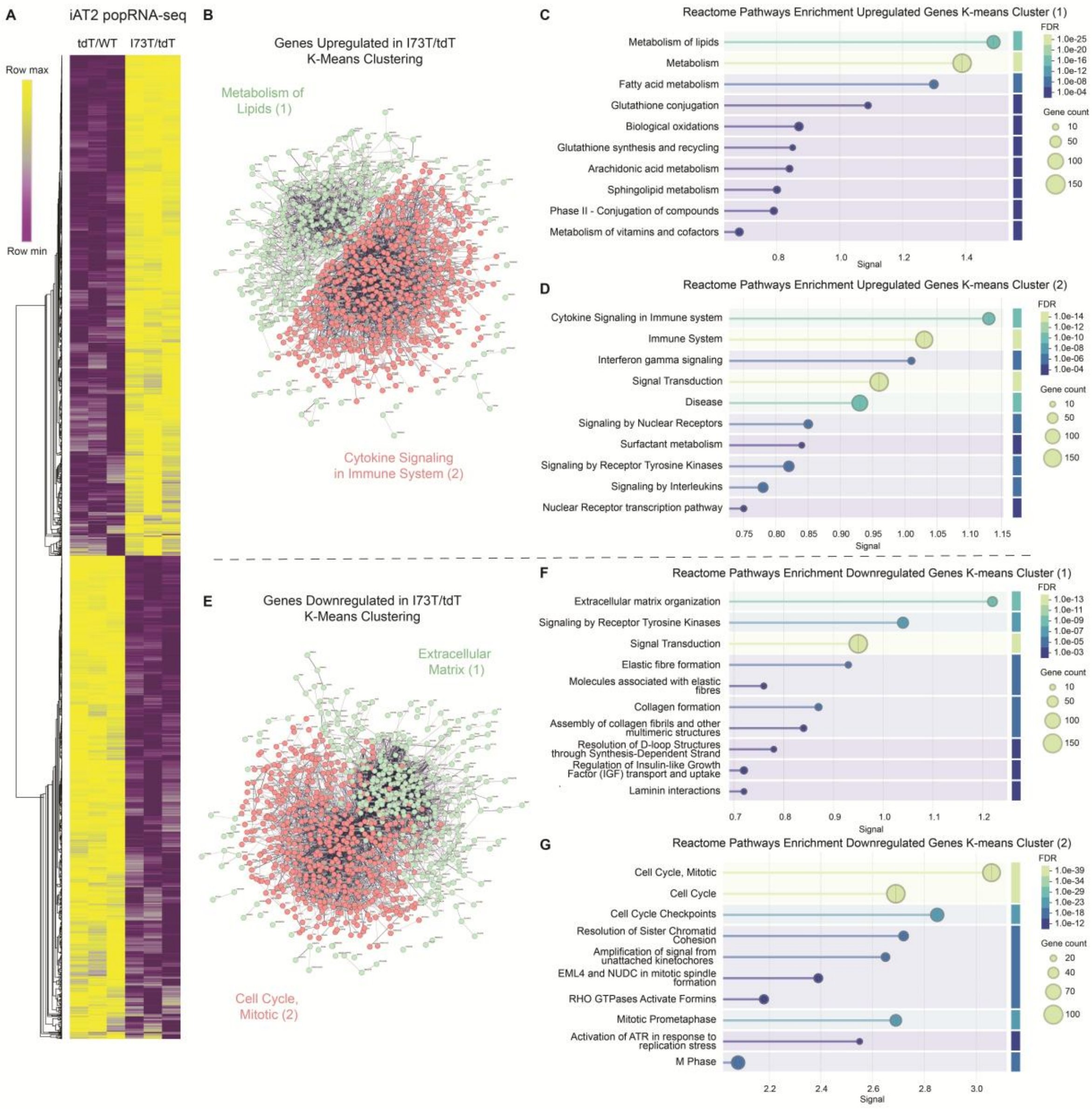

Figure S6. Population RNA-seq analysis of iAT2^{WT} and iAT2^{I73T} cells. A) Unsupervised hierarchical clustering (Euclidean distance) heatmap of differentially expressed genes (FDR <0.05) between iAT2^{WT} and iAT2^{I73T} cells (n = 3 per condition). B) Top Reactome terms (ranked by StringDB signal) in iAT2^{I73T} as compared to iAT2^{WT} using unsupervised K-means clustering (k = 2) are highlighted in StringDB network graph. C-D) Summary Reactome analysis of top 10 terms enriched in each K-means cluster. E-G) String DB analysis of downregulated genes iAT2^{I73T} as compared to iAT2^{WT} and Reactome clusters as described in B-D.

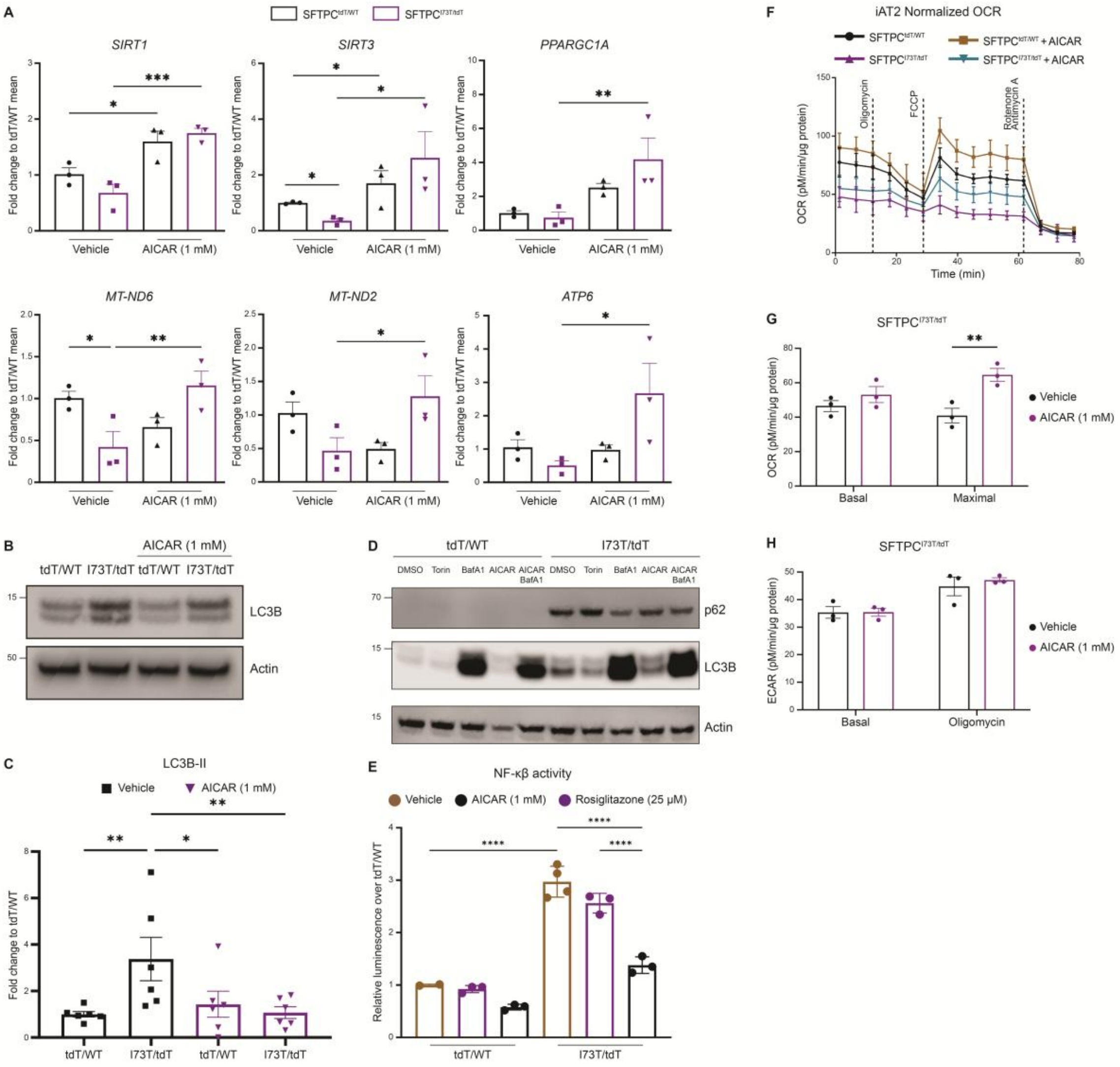
A
SFTPC tdT/WT
SFTPC I73T/tdT
SIRT1
SIRT3
PPARGC1A
MT-ND6
MT-ND2
ATP6
Fold change to tdT/WT mean
Vehicle
AICAR (1 mM)
B
AICAR (1 mM)
tdT/WT
I73T/tdT
LC3B
Actin
C
LC3B-II
Vehicle
Fold change to tdT/WT
D
DMSO
Torin
BafA1
AICAR
p62
E
NF-κβ activity
Rosiglitazone (25 μM)
Relative luminescence over tdT/WT
F
iAT2 Normalized OCR
SFTPC tdT/WT + AICAR
SFTPC I73T/tdT + AICAR
Oligomycin
FCCP
Rotenone
Antimycin A
OCR (pM/min/μg protein)
Time (min)
G
Basal
Maximal
H
ECAR (pM/min/μg protein)

Figure S7. AMPK agonism ameliorates impaired mitochondrial biogenesis, autophagy defect, and inflammatory activation in iAT2^{I73T} cells. A) RT-qPCR of PGC1α target genes demonstrates increased mitochondrial biogenesis in iAT2^{I73T} cells after treatment with AICAR (1 mM, 24 hours) (mean ± SEM; n = 3 biological replicates). B) Representative immunoblot of LC3B expression in iAT2^{WT} and iAT2^{I73T} cells after AICAR treatment (1 mM, 24 hours). C) Densitometric quantification (mean ± SEM; n = 6 biological replicates), normalized to loading control and presented as fold change over the corresponding iAT2^{WT} mean included in each immunoblot. D) Immunoblot of autophagy markers p62 and LC3B in cell lysates of iAT2^{WT} and iAT2^{I73T} cells following overnight treatment with Torin (5 μM), Bafilomycin A (50 nM), AICAR (1 mM), or a combination of Bafilomycin A and AICAR. E) Bioluminescence quantification of canonical NF-κB signaling activity in iAT2^{WT} and iAT2^{I73T} cells transduced with an NF-κB-luc-GFP lentiviral vector, followed by overnight treatment with AICAR (1 mM) or Rosiglitazone (25 mM). NF-κB activity was elevated in iAT2^{I73T} cells and significantly reduced following AMPK agonism (mean ± SD; n = 3 biological replicates). F-H) OCR and ECAR quantification in iAT2^{I73T} cells following AICAR treatment (1 mM, 24 hours) (mean ± SEM; n = 3 biological replicates). $^*p < 0.05$ ** $p<0.005$ *** $p<0.005$, by ordinary one-way ANOVA.

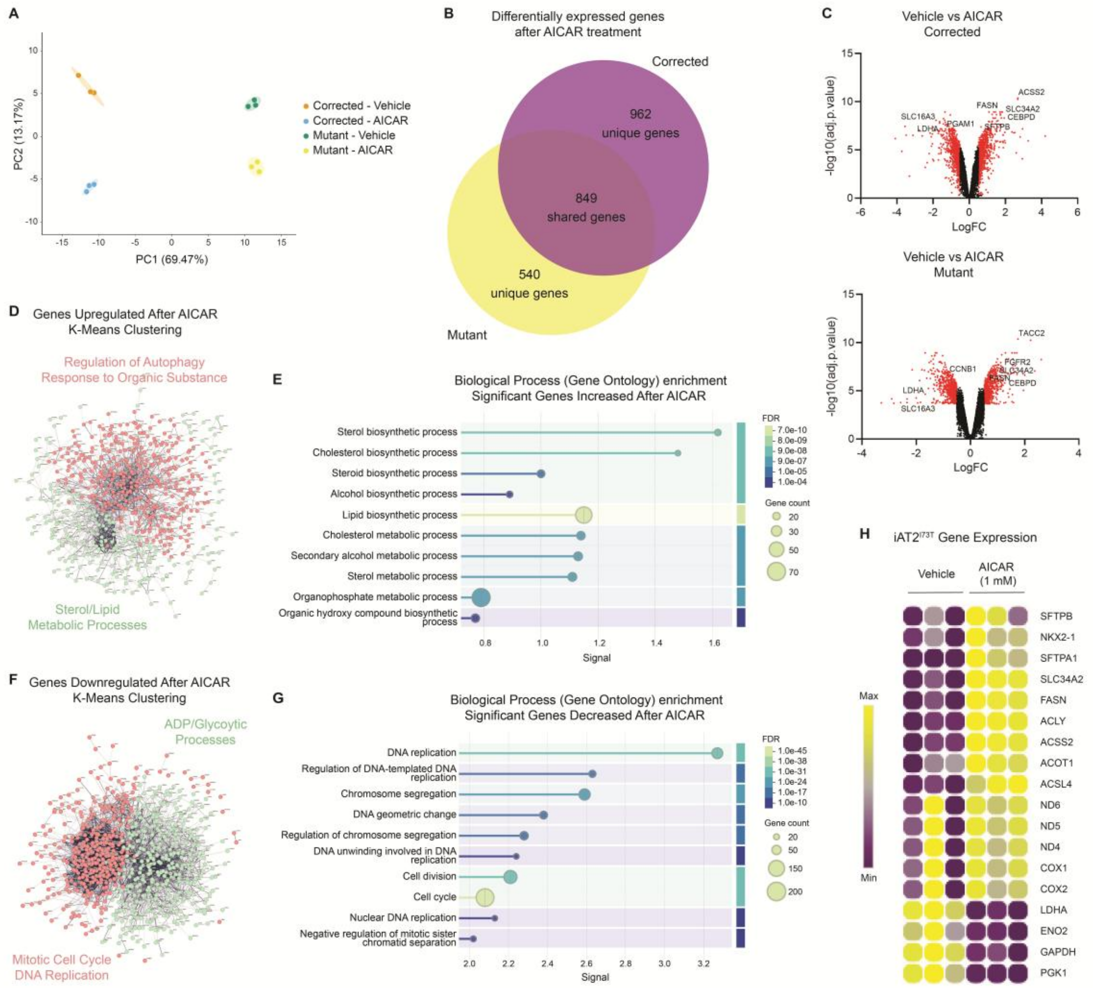

Figure S8. Population RNA-seq analysis of iAT2^{WT} and iAT2^{I73T} cells treated with vehicle or AICAR. A) Principal component analysis (PCA) of log2 normalized counts from iAT2^{WT} and iAT2^{I73T} cells following overnight treatment with AICAR (1 mM) or vehicle (n = 3 per condition). B) Number of differentially expressed genes ($log_2FC > 1.5$, FDR <0.05) in iAT2^{WT} and iAT2^{I73T} cells following overnight AICAR treatment. C) Volcano plot of differentially expressed genes ($log_2FC > 1.5$, FDR <0.05) in iAT2^{WT} and iAT2^{I73T} cells following AICAR treatment. D, E) String DB unsupervised K-means clustering (k = 2) of genes upregulated in both iAT2^{WT} and iAT2^{I73T} cells following AICAR treatment. D) Top Reactome terms (ranked by gene number) associated with each K-means cluster are highlighted E) Top 10 enriched GO terms, shown with FDR and total gene counts. F) Top Reactome terms (ranked by gene number) associated with each K-means cluster are highlighted. G) Top 10 enriched GO terms, shown with FDR and total gene counts. (H) Heatmap of select genes from Figure 6D associated with AT2 cell homeostasis, fatty acid synthesis, mitochondrial subunits, and glycolysis, highlighting changes in iAT2^{I73T} cells associated with AT2 cell homeostasis, fatty acid synthesis, mitochondrial subunits, and glycolysis.

-

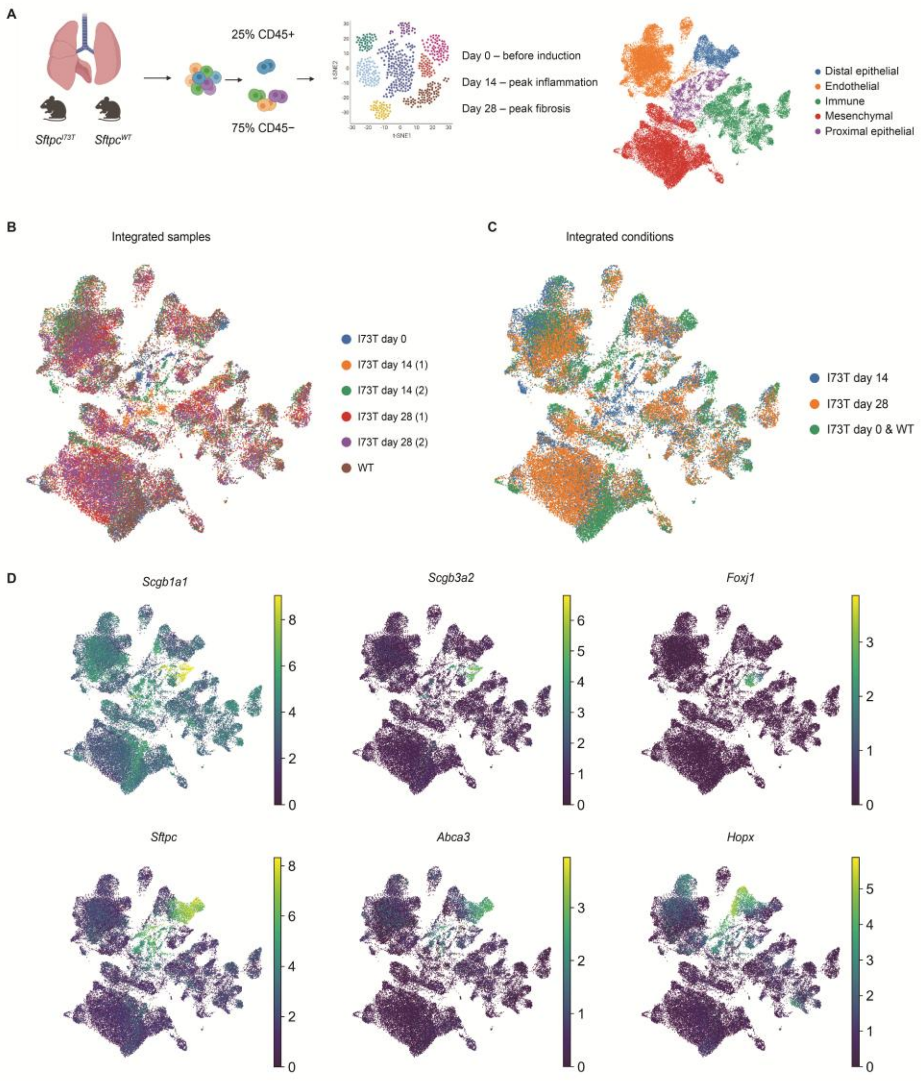

Figure S9. Cell Type annotation in scRNA-seq data from *Sftpc^{I73T}* mice at 28 days post tamoxifen induction, treated with either vehicle or metformin alongside WT controls. (A) UMAP projection of the integrated dataset identifies the four major cell compartments of the murine lungs. (B) UMAP projection with color-coded treatment groups. (C) UMAP projections highlighting genes used to annotate major compartment subsets.

REFERENCES

1. Nureki S-I, et al. Expression of mutant Sftpc in murine alveolar epithelia drives spontaneous lung fibrosis. *J Clin Invest*. 2018;128(9):4008–4024.

2. Alysandratos K-D, et al. Patient-specific iPSCs carrying an SFTPC mutation reveal the intrinsic alveolar epithelial dysfunction at the inception of interstitial lung disease. *Cell Reports*. 2021;36(9). https://doi.org/10.1016/j.celrep.2021.109636.

3. Jacob A, et al. Differentiation of Human Pluripotent Stem Cells into Functional Lung Alveolar Epithelial Cells. *Cell Stem Cell*. 2017;21(4):472-488.e10.

4. Jacob A, et al. Derivation of self-renewing lung alveolar epithelial type II cells from human pluripotent stem cells. *Nat Protoc*. 2019;14(12):3303–3332.

5. Hawkins F, et al. Prospective isolation of NKX2-1-expressing human lung progenitors derived from pluripotent stem cells. *J Clin Invest*. 2017;127(6):2277–2294.

6. Katzen J, et al. An SFTPC BRICHOS mutant links epithelial ER stress and spontaneous lung fibrosis. *JCI Insight*. 2019;4(6). https://doi.org/10.1172/jci.insight.126125.

7. Headley L, et al. Low-dose administration of bleomycin leads to early alterations in lung mechanics. *Experimental Physiology*. 2018;103(12):1692–1703.

8. STAR: ultrafast universal RNA-seq aligner | Bioinformatics | Oxford Academic [Internet]. https://academic.oup.com/bioinformatics/article/29/1/15/272537. Accessed October 14, 2023.

9. Law CW, et al. voom: precision weights unlock linear model analysis tools for RNA-seq read counts. *Genome Biology*. 2014;15(2):R29.

10. Liberzon A, et al. The Molecular Signatures Database Hallmark Gene Set Collection. *cels*. 2015;1(6):417–425.

11. Mootha VK, et al. PGC-1α-responsive genes involved in oxidative phosphorylation are coordinately downregulated in human diabetes. *Nat Genet*. 2003;34(3):267–273.

12. Subramanian A, et al. Gene set enrichment analysis: A knowledge-based approach for interpreting genome-wide expression profiles. *Proceedings of the National Academy of Sciences*. 2005;102(43):15545–15550.

13. Wolf FA, Angerer P, Theis FJ. SCANPY: large-scale single-cell gene expression data analysis. *Genome Biology*. 2018;19(1):15.

14. Gayoso A, et al. A Python library for probabilistic analysis of single-cell omics data. *Nat Biotechnol*. 2022;40(2):163–166.

15. Traag VA, Waltman L, van Eck NJ. From Louvain to Leiden: guaranteeing well-connected communities. *Sci Rep*. 2019;9(1):5233.

16. Faure L, et al. scFates: a scalable python package for advanced pseudotime and bifurcation analysis from single-cell data. *Bioinformatics*. 2022;39(1):btac746.

17. Fang Z, Liu X, Peltz G. GSEApy: a comprehensive package for performing gene set enrichment analysis in Python. *Bioinformatics*. 2022;39(1):btac757.

18. Katzen J, et al. Disruption of proteostasis causes IRE1 mediated reprogramming of alveolar epithelial cells. *Proceedings of the National Academy of Sciences*. 2022;119(43):e2123187119.

19. Rodriguez L, et al. Chronic Expression of a Clinical SFTPC Mutation Causes Murine Lung Fibrosis with IPF Features. *Am J Respir Cell Mol Biol*. [published online ahead of print: December 6, 2022]. https://doi.org/10.1165/rcmb.2022-0203MA.

20. Assali EA, et al. Nanoparticle-mediated lysosomal reacidification restores mitochondrial turnover and function in β cells under lipotoxicity. *FASEB J*. 2019;33(3):4154–4165.

21. Taddeo EP, et al. Mitochondrial Proton Leak Regulated by Cyclophilin D Elevates Insulin Secretion in Islets at Nonstimulatory Glucose Levels. *Diabetes*. 2020;69(2):131–145.

22. Rodriguez LR, et al. PGF2α signaling drives fibrotic remodeling and fibroblast population dynamics in mice. *JCI Insight*. [published online ahead of print: November 7, 2023]. https://doi.org/10.1172/jci.insight.172977.
