# Supplementary material for: Impaired AMPK control of alveolar epithelial cell metabolism promotes pulmonary fibrosis
Source: JCI Insight. 2025 Jul 1;10(15):e182578. doi: 10.1172/jci.insight.182578 (PMC12333953; doi:10.1172/jci.insight.182578)

Figure 1D LDHA

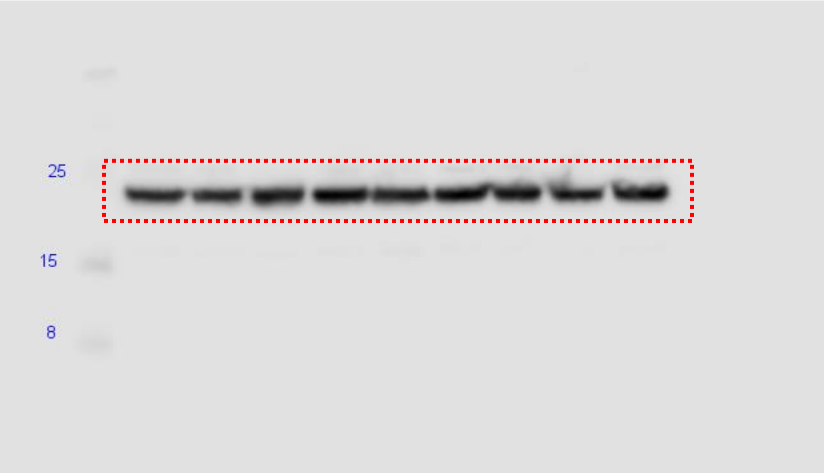

ß-Actin for LDHA

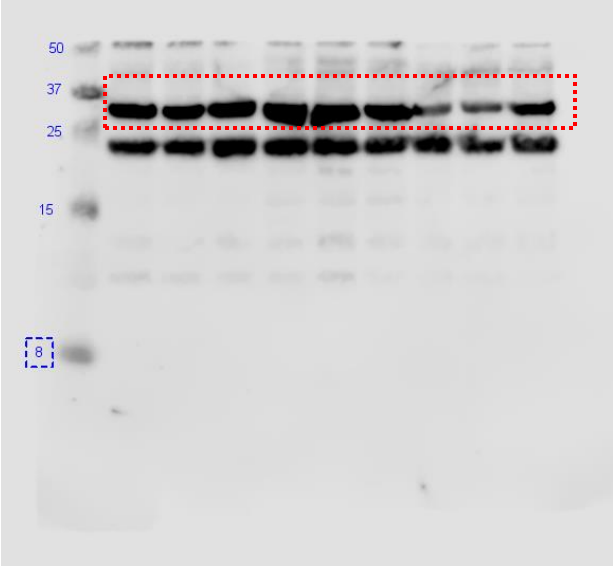

Figure 1D LDHB

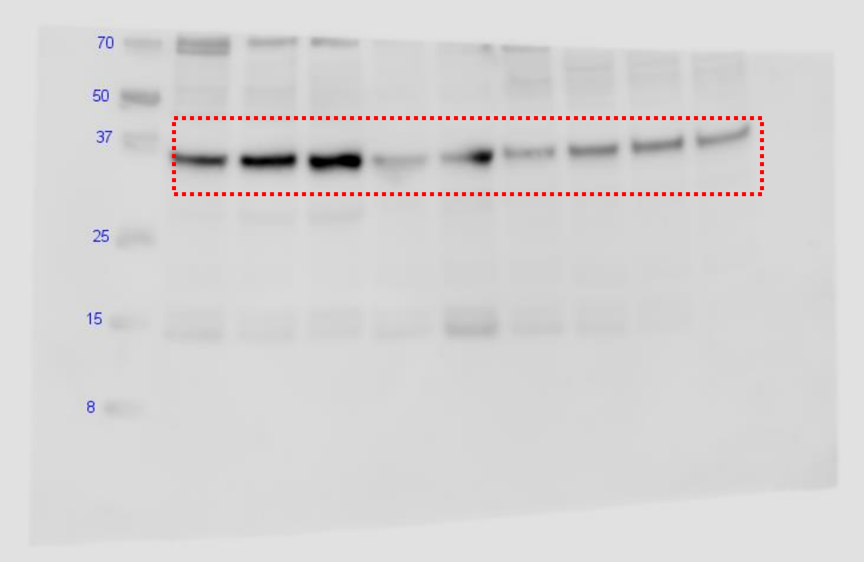

HK1 for LDHB

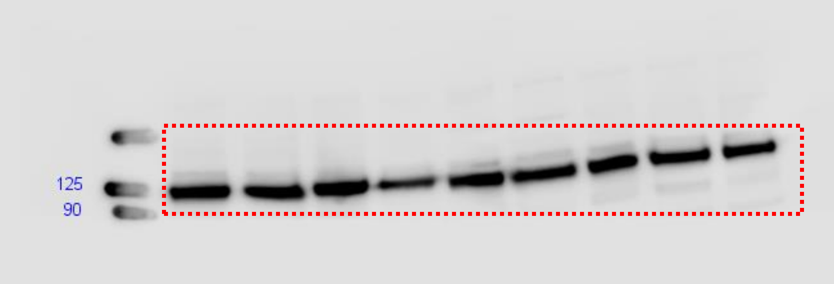

Figure 2J PINK1

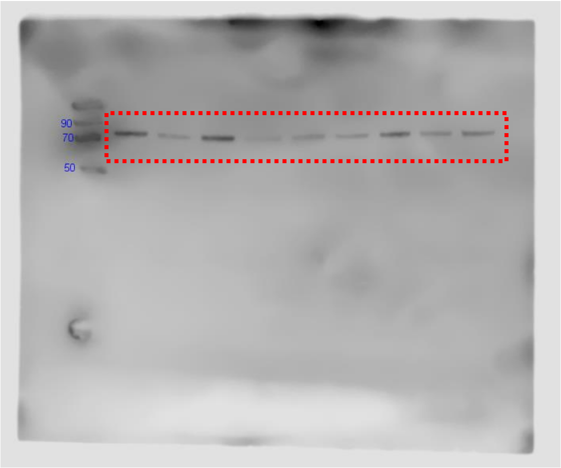

Figure 1D HK1

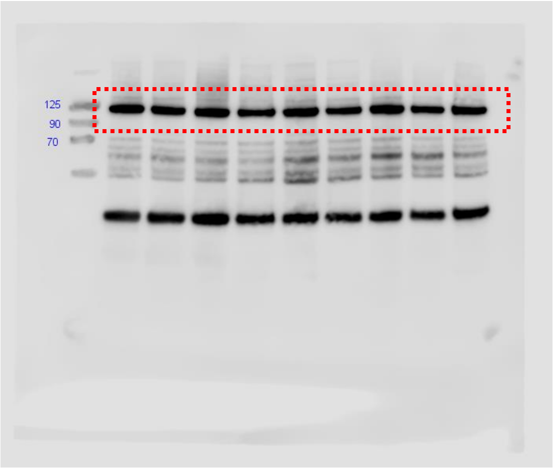

Figure 2J PARKIN

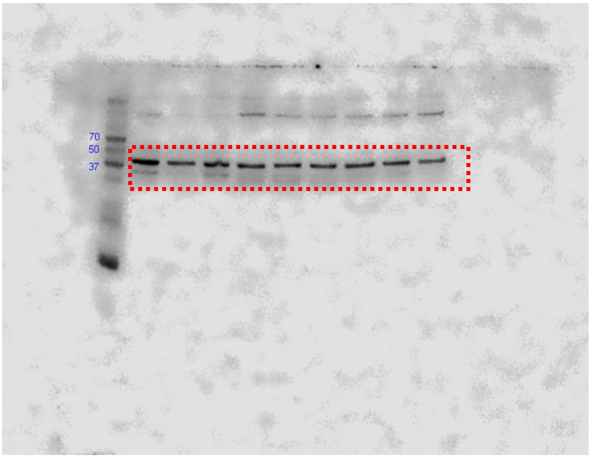

Figure 1D MDH2

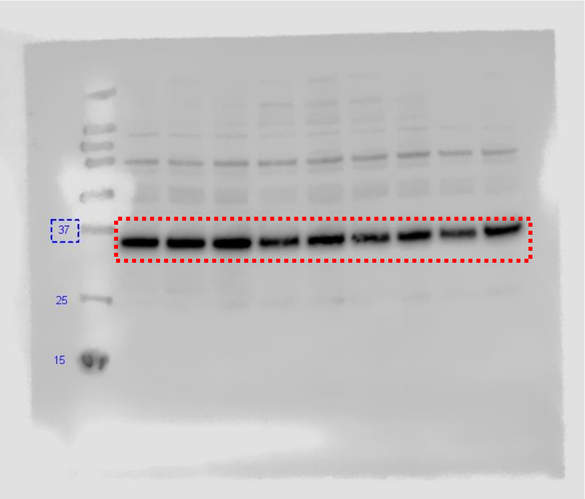

Figure 1D ß-ACTIN for PINK1, MDH2, HK1

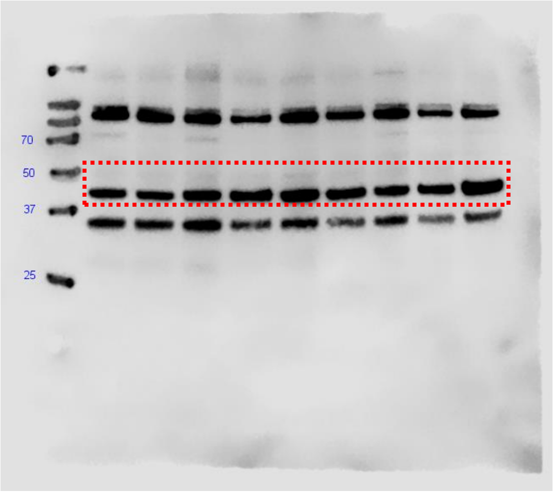

ß-ACTIN for PARKIN

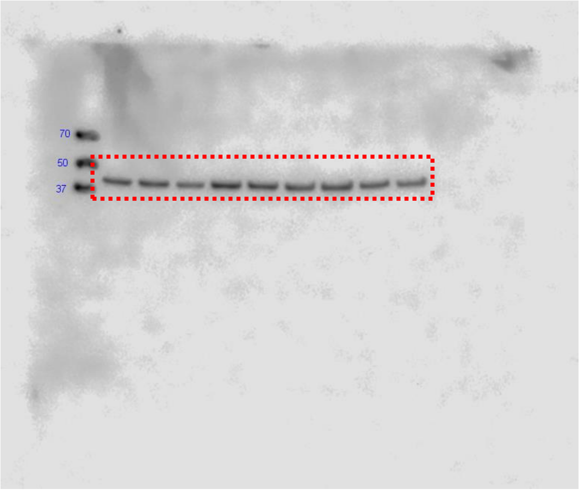

Figure 2J pDRP1

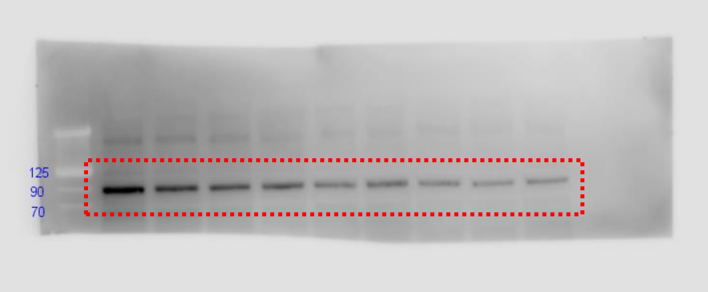

Figure 1J Total DRP1

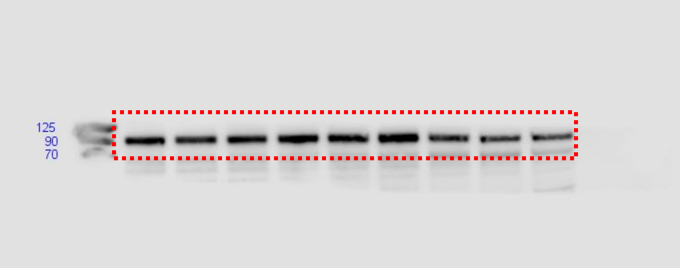

Figure 2J pMFF

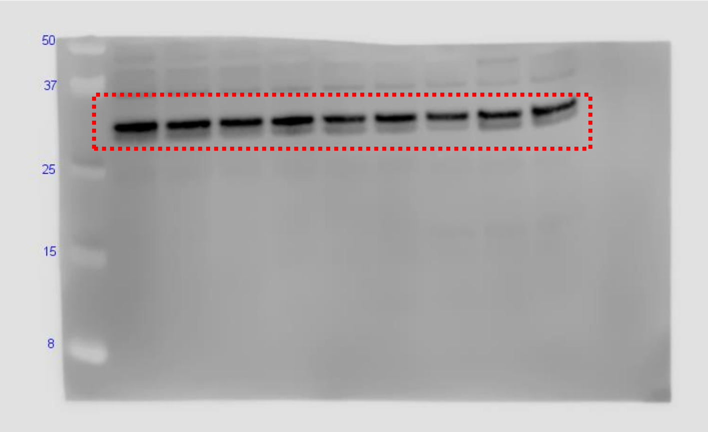

Figure 2J Total MFF

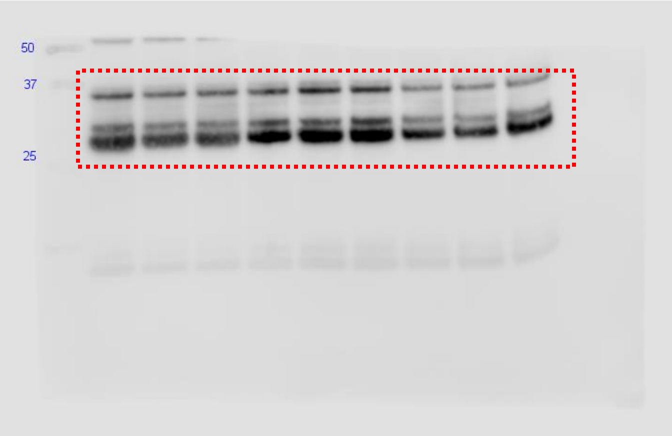

Figure 2J ß-Actin

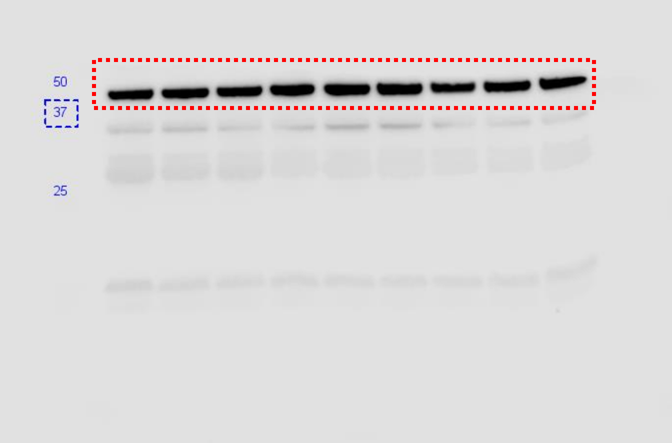

Figure 2B
pPGC1a

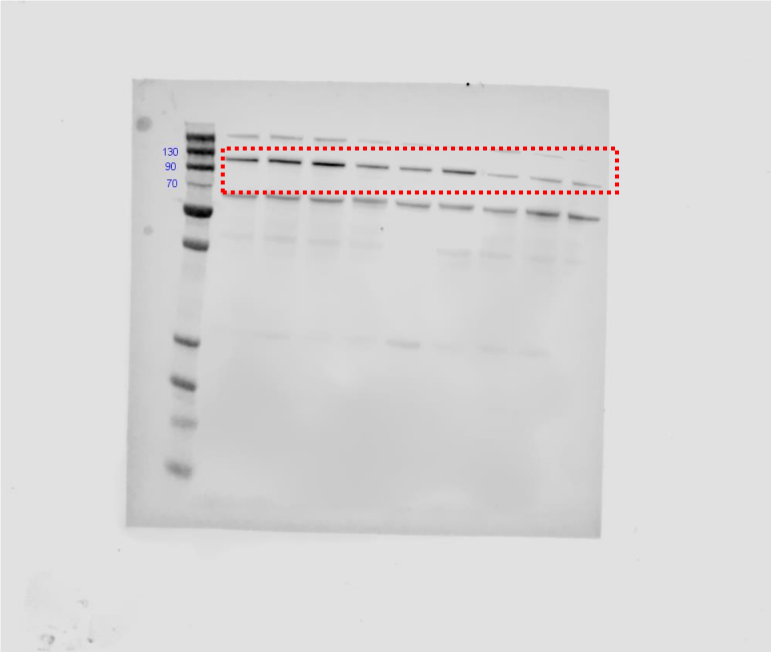

Figure 2B
PGC1a

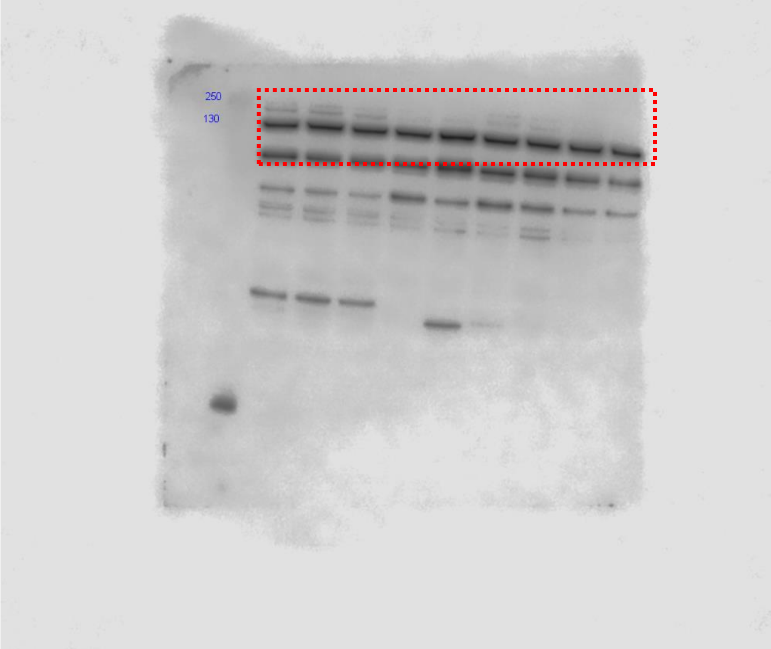

Figure 2B
Actin

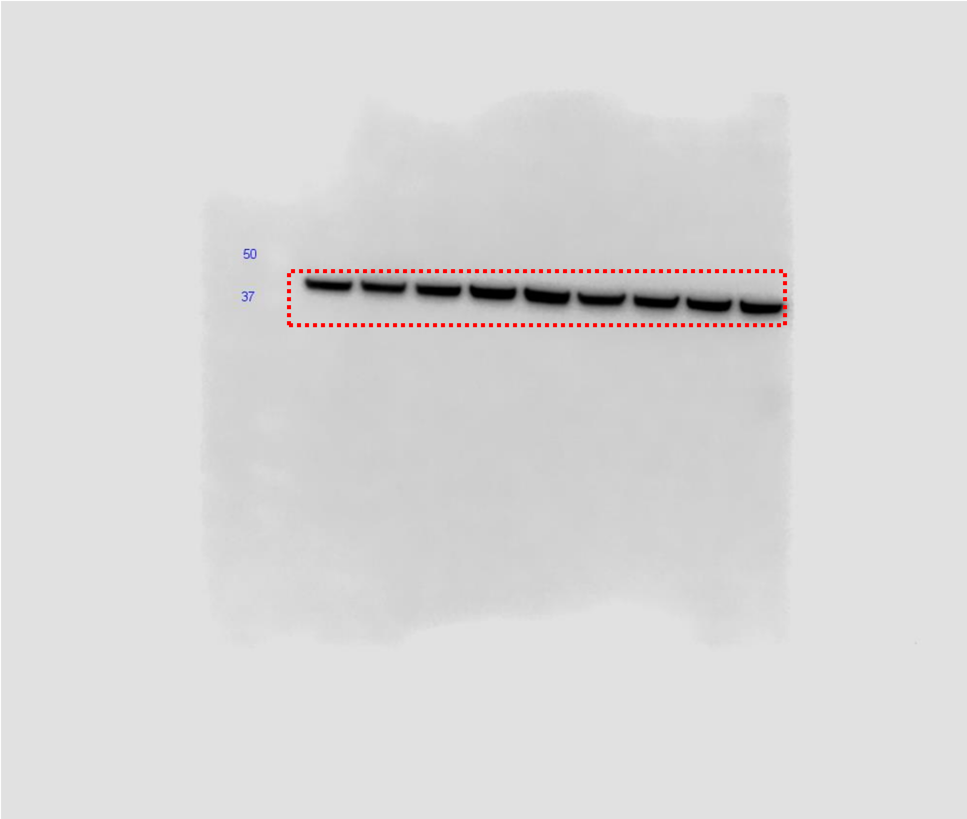

Figure 3C ACC

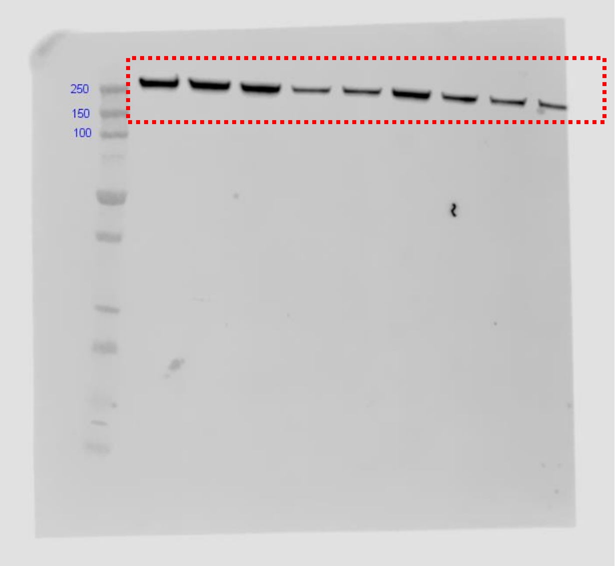

Figure 3C AMPK

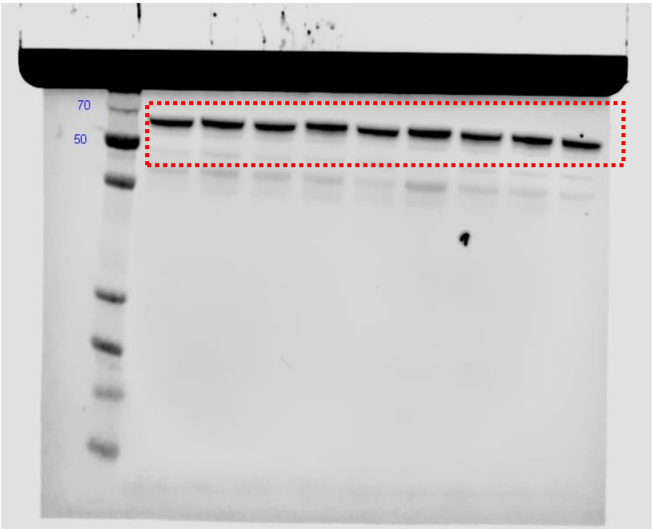

Figure 3C pAMPK

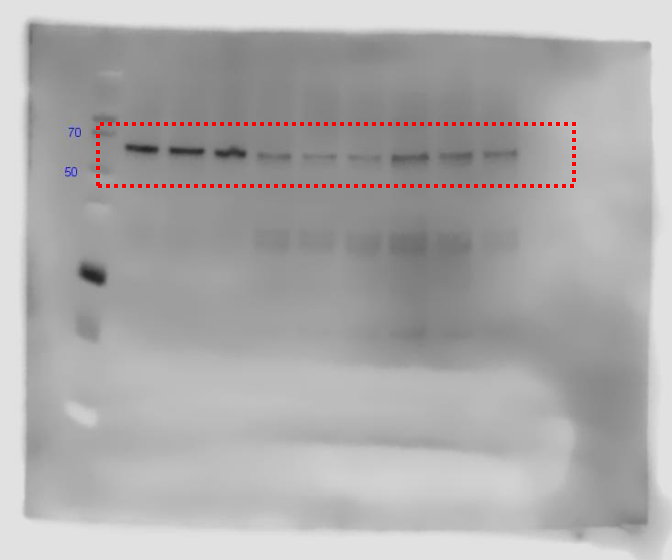

Figure 3C pACC

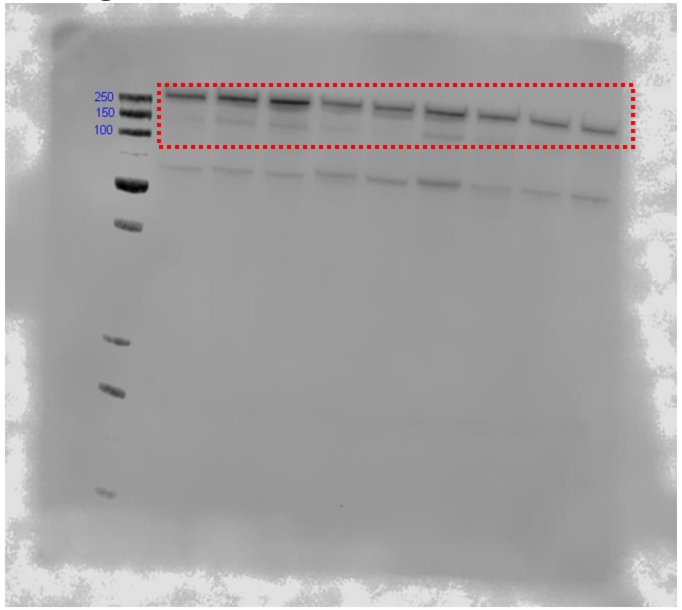

Figure 3C ß-Actin for ACC, pACC, AMPK

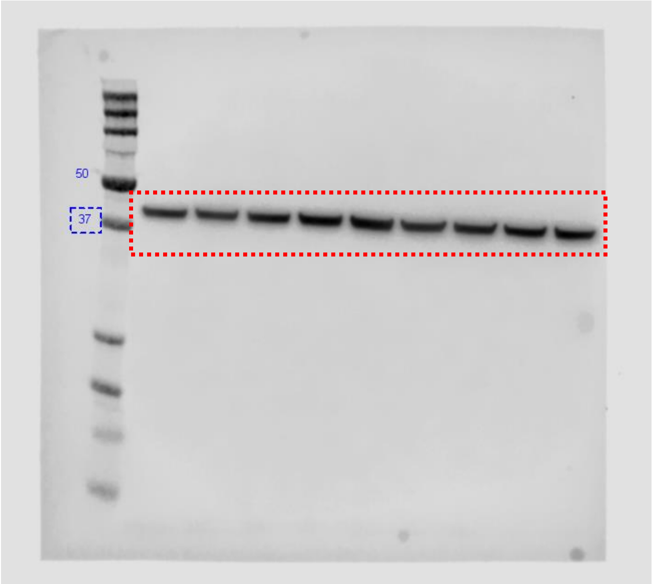

ß-Actin for pAMPK

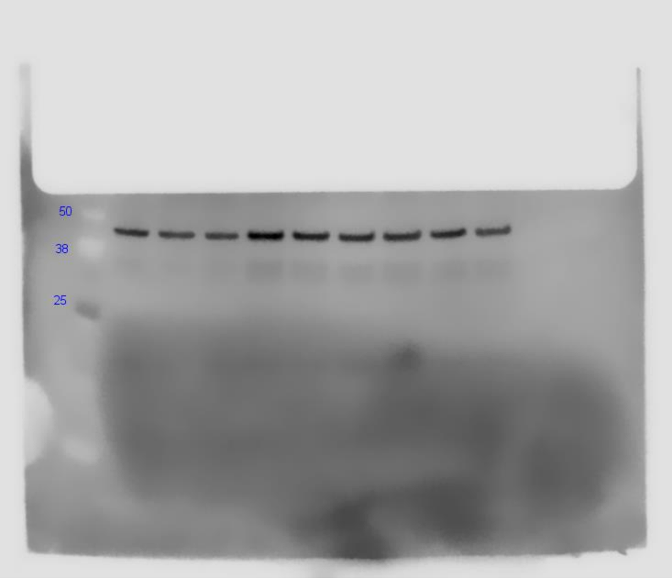

Figure 6A AMPK

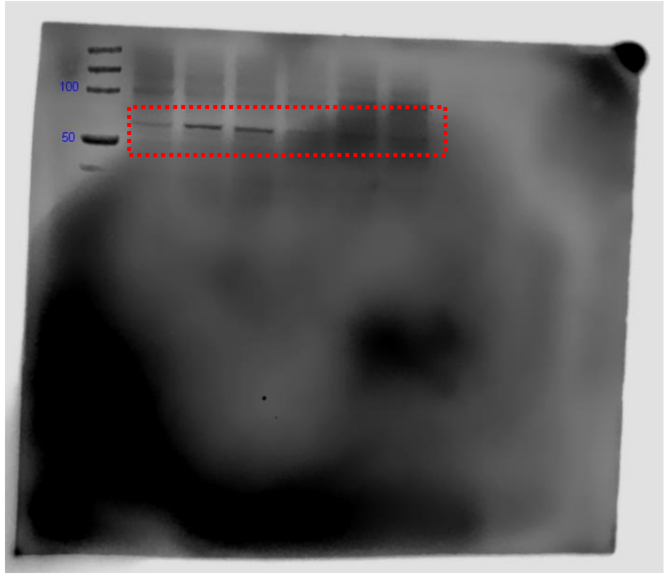

Figure 6A pAMPK

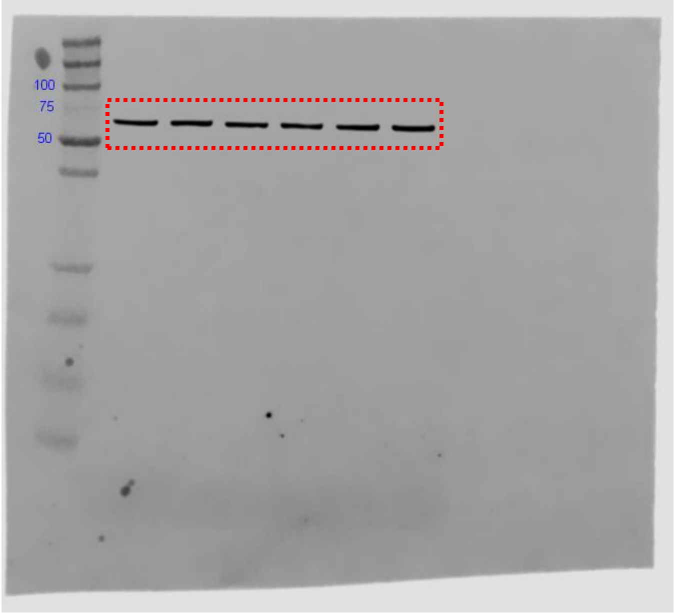

Actin

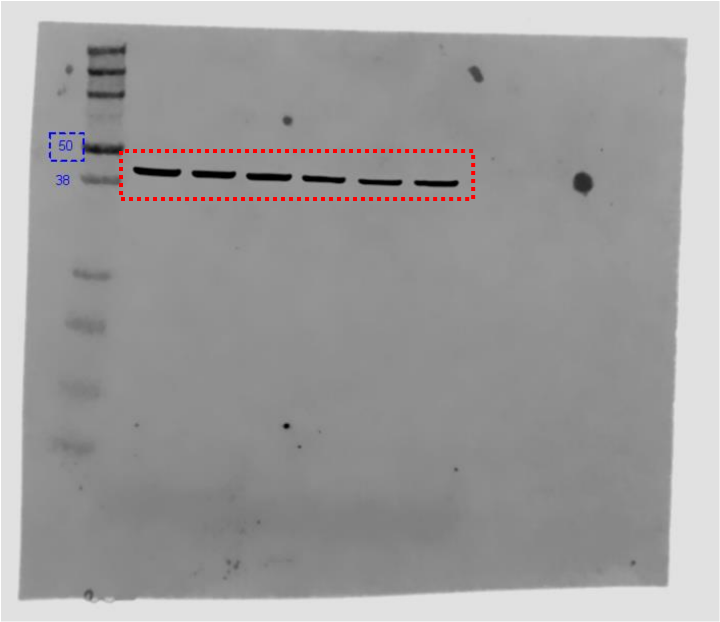

Figure 6A
LDHb

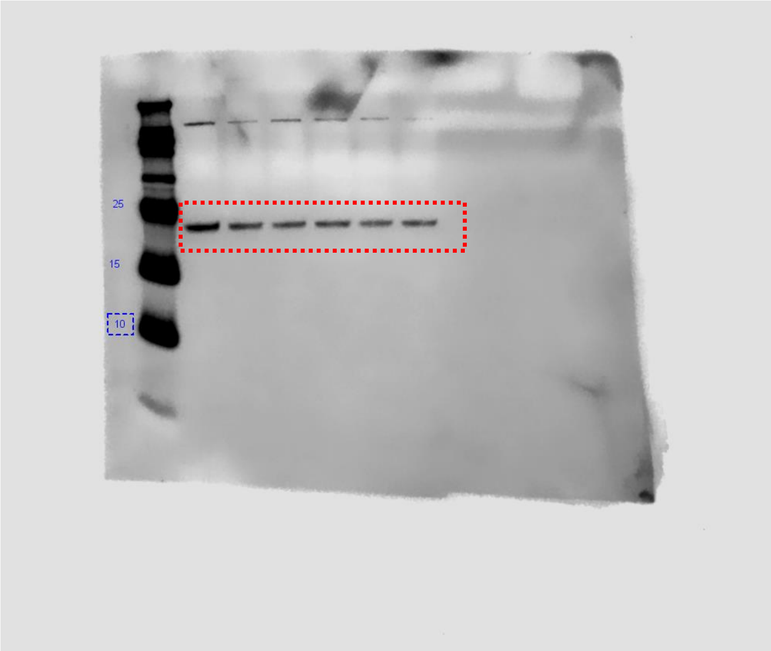

Figure 6A
LDHa

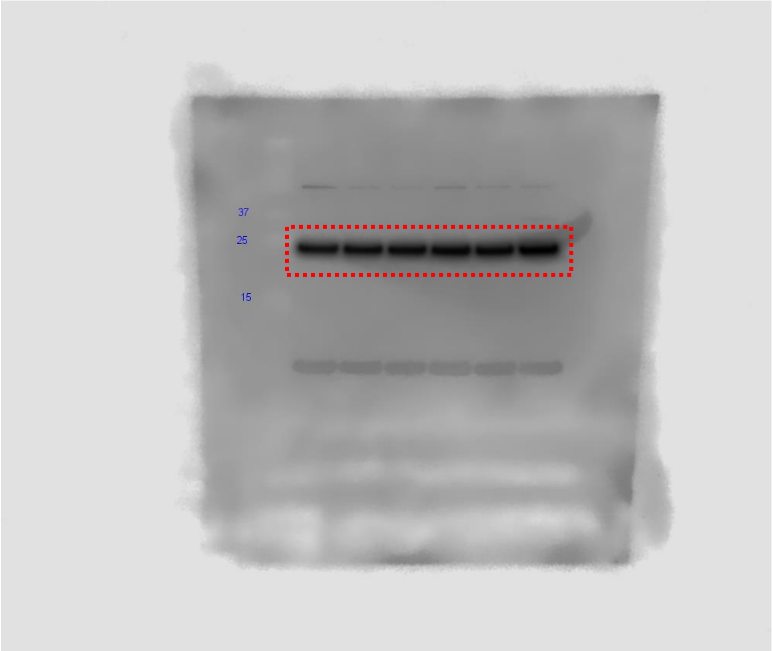

Figure 6A
Actin

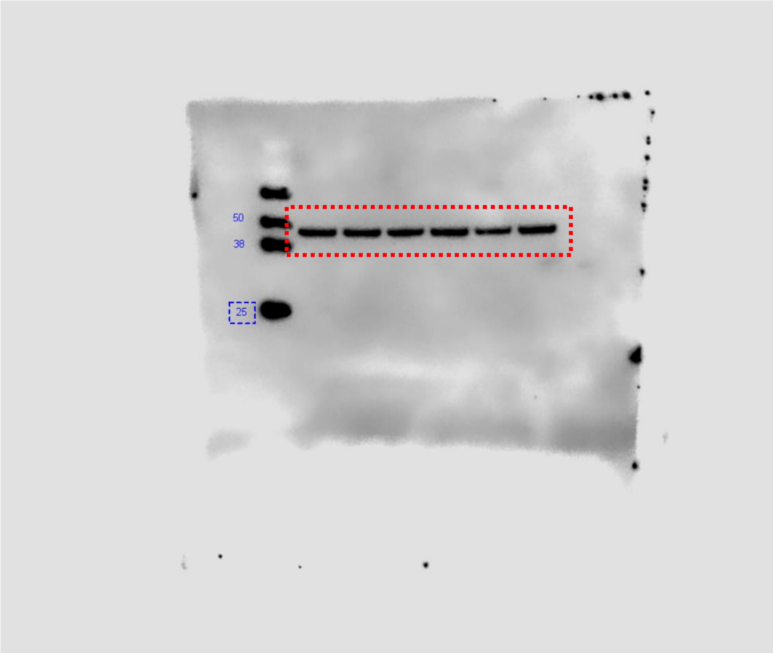

Figure 6A
Pro SPC

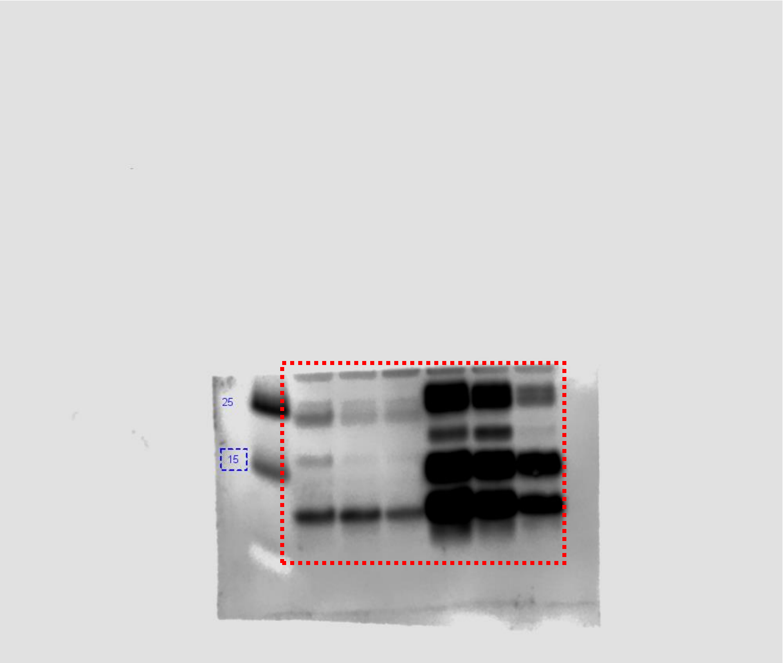

Figure 6E pACC

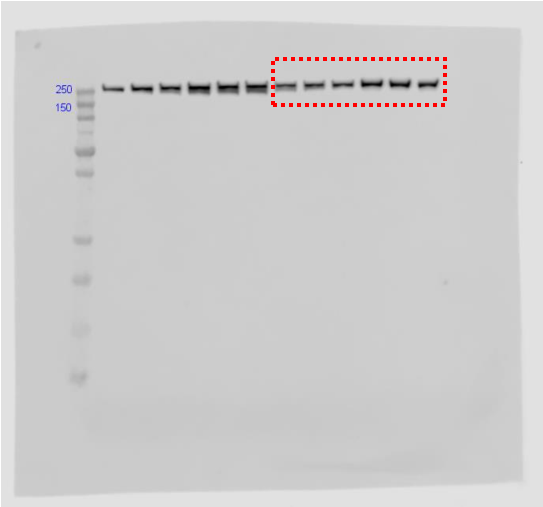

ß-Actin for pACC

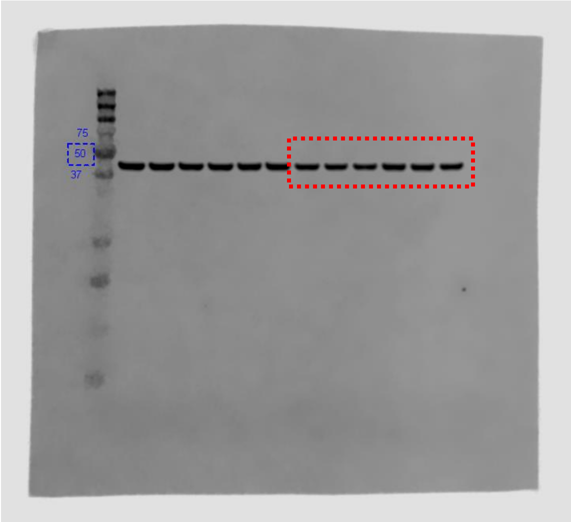

Figure 6E AMPK

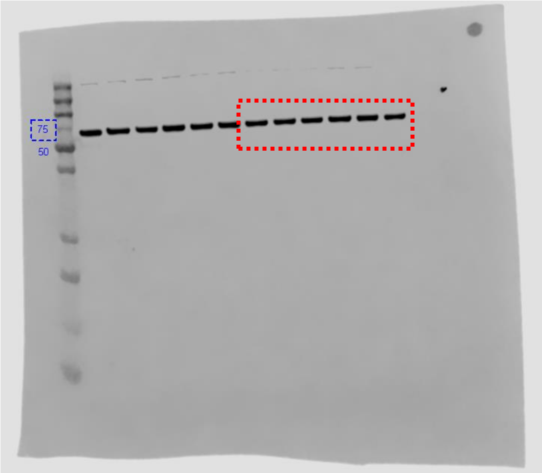

Figure 6E PGC1a

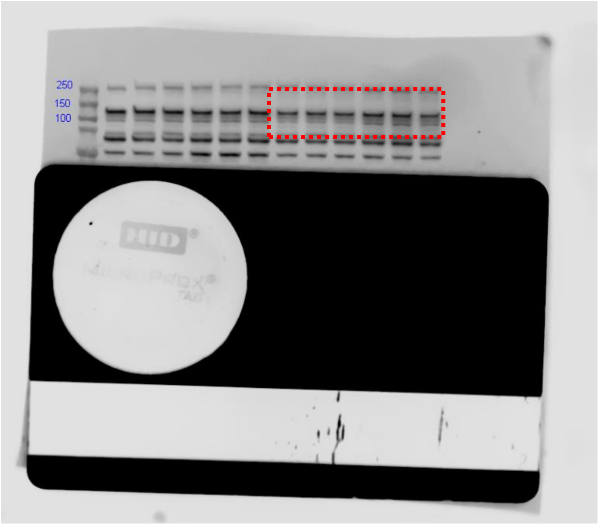

Figure 6E pAMPK

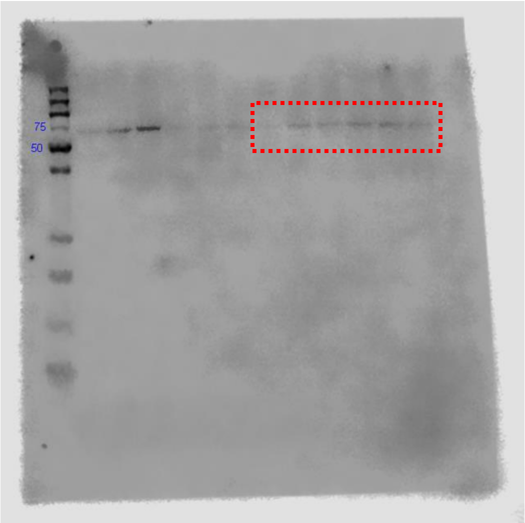

Figure 6E Actin for AMPK, PGC1a, pAMPK

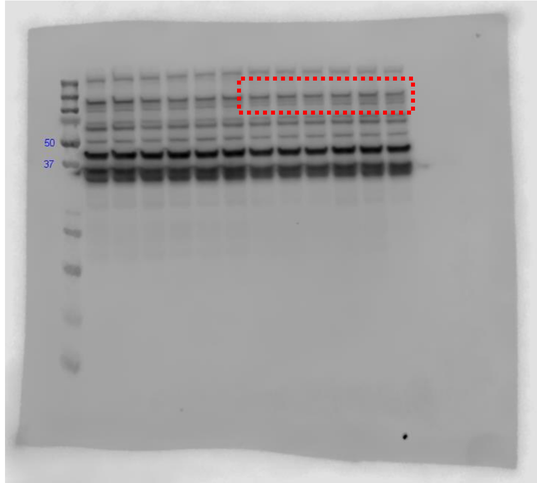

Figure 6E

pPGC1a

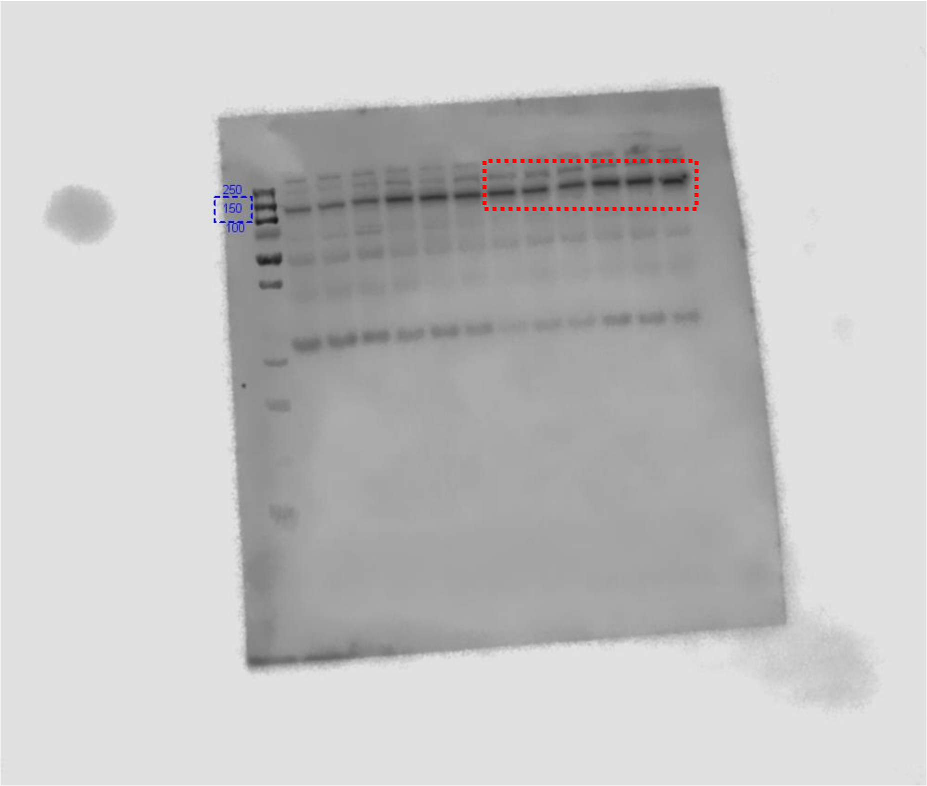

GAPDH

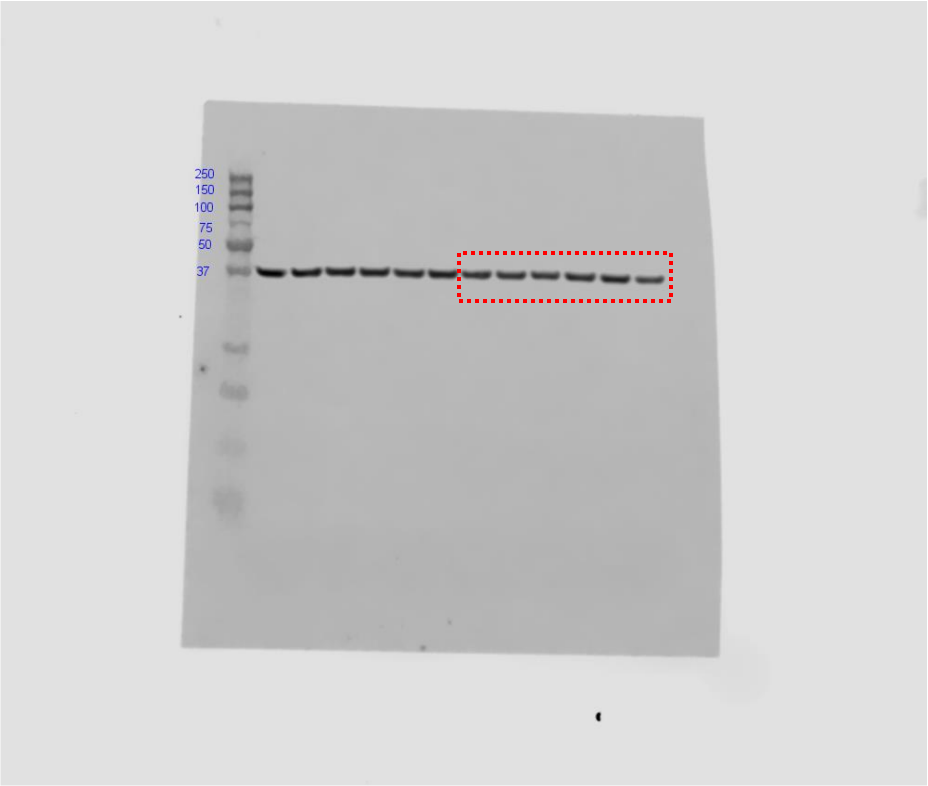

Supplement: Unedited blot and gel images [file jciinsight-10-182578-s056.pdf]
